# Supplementary material for: Commensal gut bacteria employ de-chelatase HmuS to harvest iron from heme
Source: EMBO J. 2025 Sep 12;44(21):6226–52. doi: 10.1038/s44318-025-00563-5 (PMC12583661; doi:10.1038/s44318-025-00563-5)
Supplement: Supplementary file 1 — Appendix [file 44318_2025_563_MOESM1_ESM.pdf]

APPENDIX

Commensal gut bacteria employ de-chelatase HmuS to harvest iron from heme

Arnab Kumar Nath[1], Ronivaldo Rodrigues da Silva[1], Colin C. Gauvin[1], Emmanuel Akpoto[1], Mensur Dlakić[2], C. Martin Lawrence[1,*], Jennifer L. DuBois[1,**]

[1]Department of Chemistry and Biochemistry, Montana State University, Bozeman, Montana 59717 USA
[2]Department of Microbiology and Cell Biology, Montana State University, Bozeman, Montana 59717 USA

Table of Contents:

		Pages
**Appendix Figure S1.**	Validating extraction and analysis methods for heme and PPIX.	2
**Appendix Figure S2.**	Schematic illustrating methods for growing, extracting, and quantifying metabolites from *B. theta* cells.	3
**Appendix Figure S3.**	Representative HPLC data illustrating quantification of heme and PPIX extracted from *B. theta* cells grown in minimal medium with increasing concentrations of BPS.	4
**Appendix Figure S4.**	Composition of the *hmu* operon from B theta VPI-5482 and locations of transposon insertions.	5
**Appendix Figure S5.**	Representative HPLC data illustrating the production of PPIX by *B. theta* cell fractions incubated with heme and NADH or ATP.	6
**Appendix Figure S6.**	Annotated protein sequence of HmuS and AlphaFold2-predicted structure used in designing the expression construct; Annotated DNA sequence of 9373 nucleotide expression construct used to generate HmuS.	7-10
**Appendix Figure S7.**	Mass spectrometric analysis of heterologously expressed HmuS confirms its identity and integrity and shows its major contaminant is principally composed of HmuS-derived peptides.	11
**Appendix Figure S8.**	Steps in the purification of recombinant HmuS illustrated by SDS-PAGE, UV/visible absorbance, and fraction color.	12
**Appendix Figure S9.**	Single particle workflow.	13
**Appendix Figure S10.**	Resolution and Orientation Diagnostics.	14
**Appendix Figure S11.**	Secondary structure description.	15-16
**Appendix Figure S12.**	Structure-based sequence alignment of CobN (6yt0), HmuS (this work) and CobN (7c6o).	17-18
**Appendix Figure S13.**	Length distribution of MRIs from the operonic set of HmuS sequences.	19
**Appendix Figure S14.**	UV/visible absorption spectroscopy illustrating heme titration to the HmuS H538A mutant.	20
**Appendix Table S1.**	Substrate and product analyses for heme-PPIX conversion reactions using *B theta* cellular fractions. These data are plotted as a bar chart in Figure 2.	21
**Appendix Table S2.**	Typical outcomes for recombinant HmuS expression and purification.	22
**Appendix Table S3.**	Cryo-EM data collection, processing, model refinement and validation.	23-24

SUPPLEMENTARY FIGURES

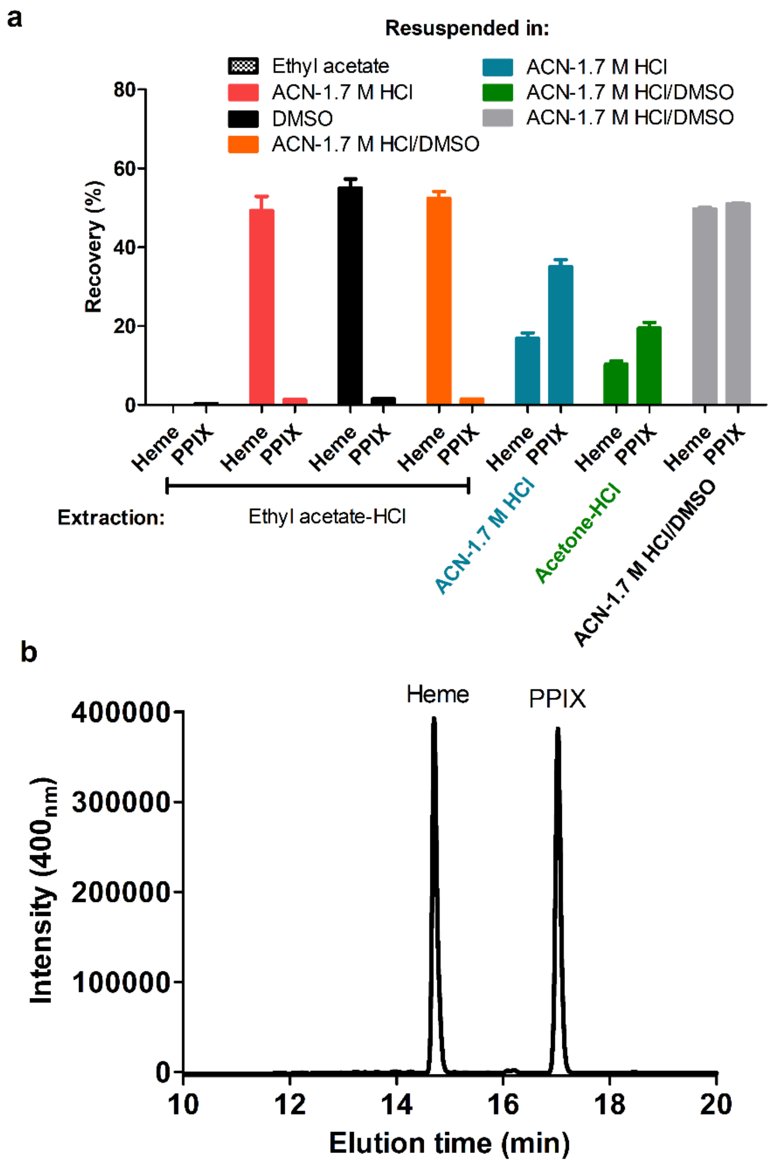

Appendix Figure S1. Validating extraction and analysis methods for heme and PPIX. (A) Efficiency of heme and PPIX recovery (plotted as % recovery = [compound measured]/(100 µM + [concentration of compound in no-standard-added control sample]). Solvents used to extract heme and PPIX from the *Escherichia coli* cell pellet are listed in the legend, where ACN = acetonitrile, HCl = 12M hydrochloric acid, and DMSO = dimethylsulfoxide (ethyl acetate, acetone, ACN:1.7 M HCl (82:18, v/v), DMSO and ACN:1.7 M HCl:DMSO (41:9:50, v/v/v). The solvents used for the extraction of heme/PPIX from *E. coli* cells are indicated on the X axis and the solvents used to resuspend the dried samples are indicated with colored bars. (B) Typical HPLC trace illustrating appearance of heme and PPIX (10 µM) and their elution time at 14.7 and 17 min, respectively, wavelength at 400 nm. The chromatography was carried out using a Hypersil GOLD™ column (Thermo Scientific™, 4.6 mm x 250 mm, 5 µm particle size), linear gradient with the solution A (ultrapure water + 0.1% trifluoroacetic acid, TFA) and solution B (Acetonitrile + 0.1% TFA), flow rate of 1 mL min^{-1}, oven temperature at 25 °C.

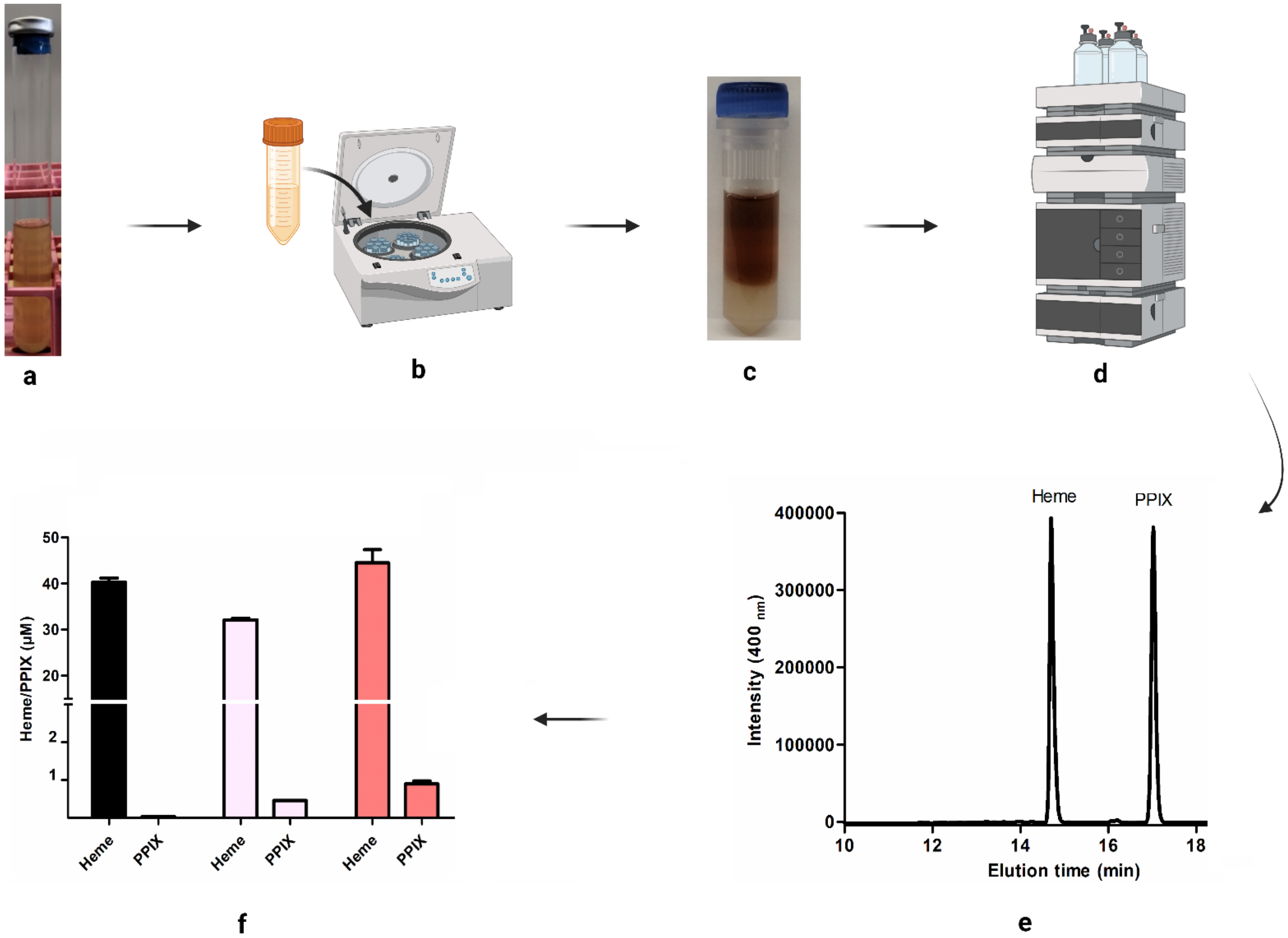

Appendix Figure S2. Schematic illustrating methods for growing, extracting, and quantifying metabolites from *B. theta* cells. (a) Cultivation of *B. thetaiotaomicron* in 10 mL Balch type, crimp-topped tubes containing minimal medium and 15 µM hemin, ±BPS, under an atmosphere composed of 2.5% H_2 and 97.5% N_2, and incubated at 37 °C, 150 rpm. (b) Bacterial cells were pelleted by centrifugation (11,900 x g, 4 °C, 15 min), washed twice in ultrapure water, and resuspended (0.12 g mL^{-1}) in the extraction solvent ACN:HCl:DMSO (41:9:50, v/v/v). (c) The cell suspensions were then transferred into FastPrep Lysis B-matrix tubes and lysed using a FastPrep 24 5g instrument (2 cycles: 6.0 meters/second for 40 sec). (d) Samples were centrifuged (9,600 x g, 25 °C, 15 min) and quantified by peak integration using HPLC. The chromatography was carried out using a Hypersil GOLD™ column (Thermo Scientific™, 4.6 mm x 250 mm, 5 µm particle size) with a linear gradient using the solution A (ultrapure water + 0.1% TFA) and solution B (Acetonitrile + 0.1% TFA), flow rate of 1 mL min^{-1}, and oven temperature at 25 °C. (e) Heme and PPIX eluted at 14.7 and 17 min, respectively. (f) Quantification of heme and PPIX was performed using a standard curve (generated by plotting standard concentrations against peak areas at 400 nm) and results were presented as bar graphs.

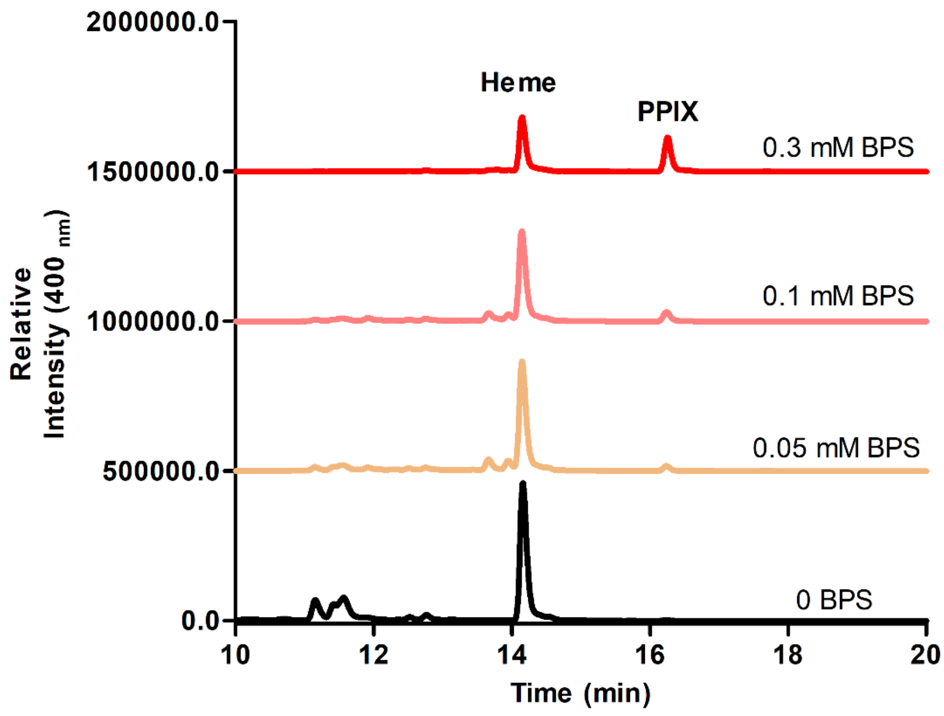

Appendix Figure S3. Representative HPLC data illustrating quantification of heme and PPIX extracted from *B. theta* cells grown in minimal medium with increasing concentrations of BPS. Representative HPLC data used in generating the data plotted in bar chart in Figure 2a are shown. Numerical data were measured from 3 biological replicates, and values are given in Table S1.

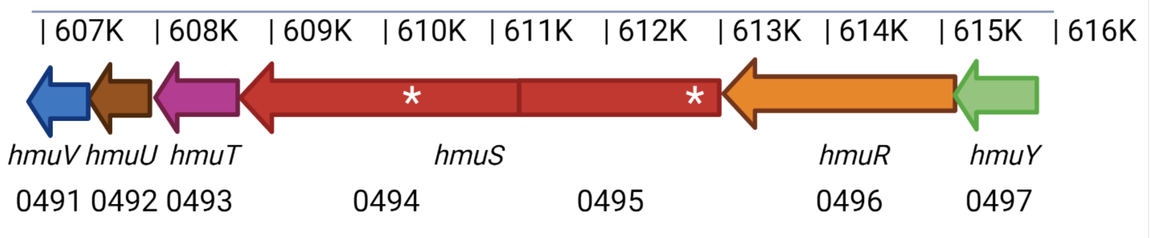

Appendix Figure S4. Composition of the *hmu* operon from B theta VPI-5482 and locations of transposon insertions. The magnified region of the complete genome between nucleotides 607K-616K (GenBank accession AE015928.1) is shown. Arrows indicate each of the *hmuYRSTUV* genes encoded on the antisense strand, relative to the nucleotide numbering shown along the top of the figure. BT loci numbers are given beneath the gene names. (Note: the *hmuS* gene was erroneously assigned two locus numbers in this genome because of a frameshift, which was later corrected.) Two *hmuS* transposon insertion mutants were generated, mapping to nucleotide positions 610,214 (*hmuS* 2, BT0495 P295-G04) and 612,897 (*hmuS* 1, BT0494 P289-C05), respectively. Approximate locations of transposon insertion are marked with a white asterisk. The transposon sequence and details of its use to generate a genomic library of mutants is described by Arjes et al[1].

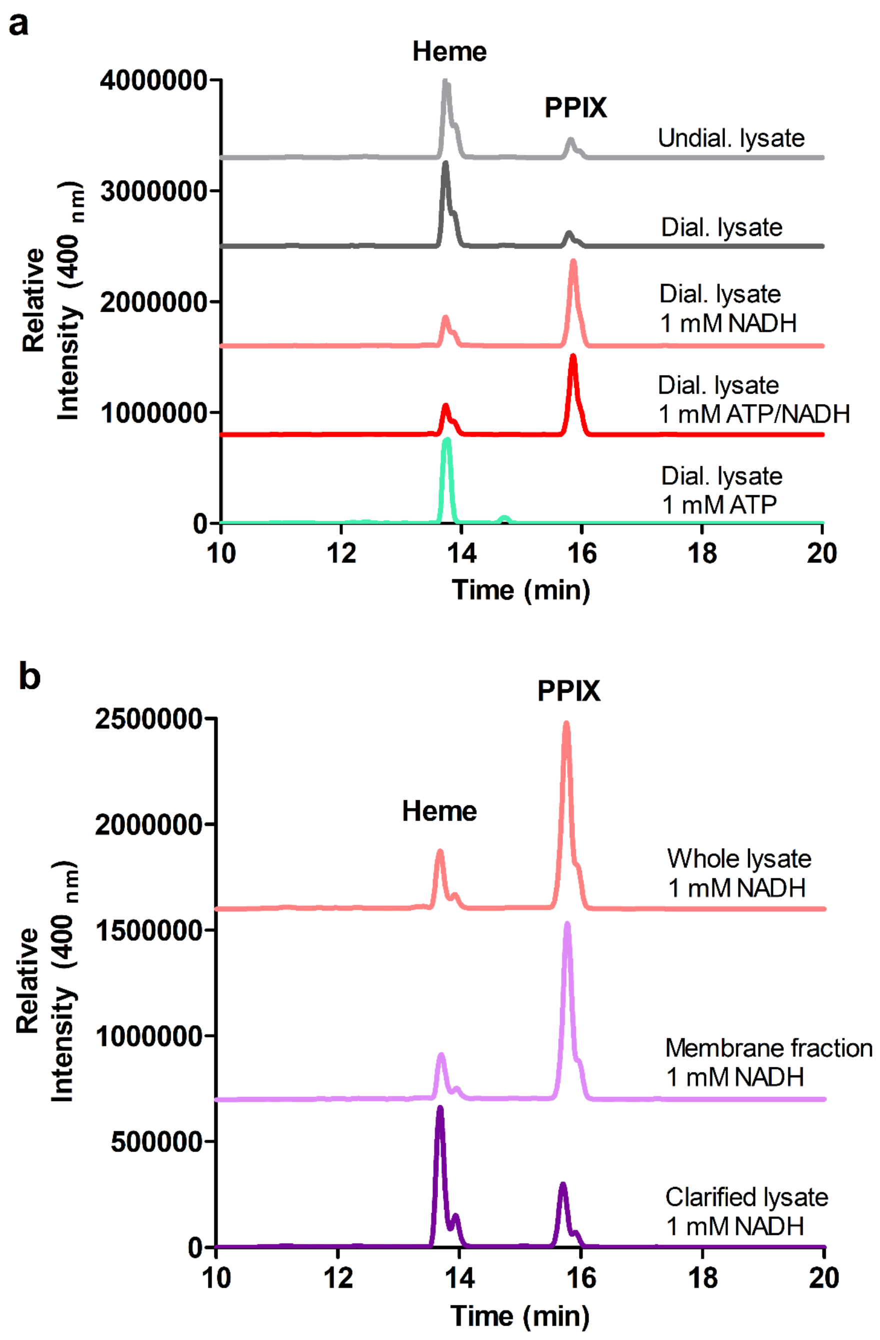

Appendix Figure S5. Representative HPLC data illustrating the production of PPIX by *B. theta* cell fractions incubated with heme and NADH or ATP. Representative HPLC data used in generating the data plotted in bar charts in Figure 2 are shown. Numerical data were measured from 3 biological replicates, and numerical values are given in Table S1.

(A) N-
MKKKSKIILGGCIVVAALIGLSVWNTWFS***ATKIAFVNFQTIQQGSISKANDNSFIKLSEVSLDNLDRLTSYDMVFING***
MGLRIVEEQRQQIQQAADKGIPVYTSMATNPANNICNLDSIQQNLIRGYLSNGGKTNYRNMLNYIRKAIDGKASA
VPEVEDPIERPSDMLYHAGISNPDDEQEFLTVADYEKFMQENNLYKEGARKIMITGQMADATDLIKALENAGYN
VYPVQSMTRFMSFIEEVQPDAVINMAHGRMGDKMVDYLKRNILLFAPLTINSLVDEWENDPMGMSGGFMSQSI
VTPEIDGAIRPFALFAQYEDKEGLRHSYAVPERLKTFVSTIDNYLNLKTKPNFEKKVAIYYYKGPGQNALTAAGM
EVVPSLYNLLLRMKQEGYNISGLPANAQELGKMIQAQGAVFNAYAEGAFNDFMQNGHPELITKEQYESWVKES
LRPEKYQEVVDAFGEFPGNYMVTPDGKLGIARLQFGNVVLLPQNAAGSGDNSFQVVHGTDMAPPHTYIASYLW
MQHGFKADALIHFGTHGSLEFTPRKQVALCSNDWPDRLVGAVPHYYLYSIGNVGEGMMAKRRSYATLQSYLT
PPFLESSVRGIYRELMEKIKIYNNSQKANKDQESLAVKTLTVKMGIHRDLGLDSMANKPYTEDEIARVENFAEEL
ATEKITGQLYTMGVPYEPERITSSVYAMATEPIAYSLFALDKQRGKATESAEKHRSVFTQQYLMPARLLVERLM
ANPSLATDELICHTAGITPQELAKARQIEAERNAPKGMMAMMMAAAAKKDQADNEPSGNGHPASAKMEKGP
HGKMPAGMKEAMKKMGANMDPEKAMEMAKSMGASPEALKKMEASMKANKDTSTDASGKPAMAGKTEKPQ
GMSAMMAAMGKAPKEYSKEEVEFALAVAEVERTIKNVGNYKNALLTSPEEELSSLMNALKGGYTAPTPGGDPI
ANPNTLPTGRNMYAINAEATPTEAWEKGIALAKQTIDRYKQRHNDSIPRKVSYTLWSSEFIETGGATIAQVLYML
GVEPVRDAFGRVSDLKLIPSTELGRPRIDVVVQTSGQLRDLAASRLFLINRAVEMAAAAKDDKYENQVASSVIE
AERVLTEKGLSPKDAREISTFRVFGGANGMYGTGIQEMVESGDRWENESEIADTYLNNMGAYYGSEKNWEVF
QKFAFEAALTRTDVVVQPRQSNTWGALSLDHVYEFMGGMNLAVRNVTGKDPDAYLSDYRNRNHMKMQELKE
AVGVESRTTILNPTYIKEKMKGGASSASEFAEVITNTYGWNVMKPAAIDKELWDNIYNVYVKDELNLGVKQYFE
QQNPAALEEMTAVMLESARKGLWQASEEQVAELSKLHTEIVNTYRPSCSGFVCDNAKLRDFIASKADAQTATQ
YKENISKIRAKASGSNKGVVMKKEEMNQTAENQTNTLSNVAVGIAVIIVILALILFVRKRRKSSQM-C

(B)

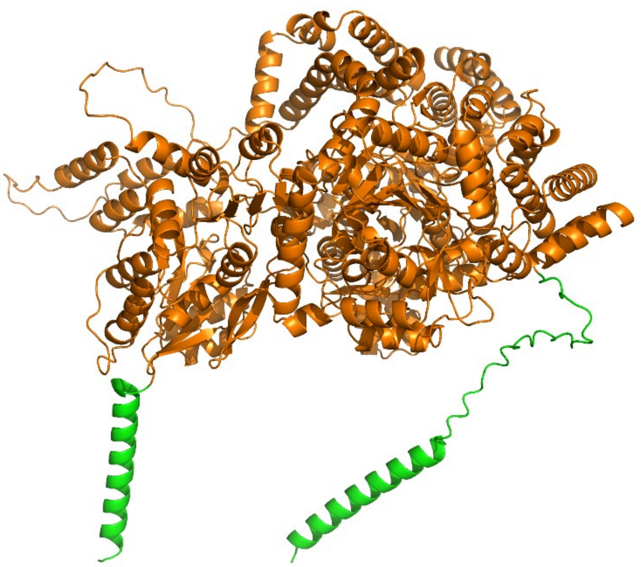

(C)

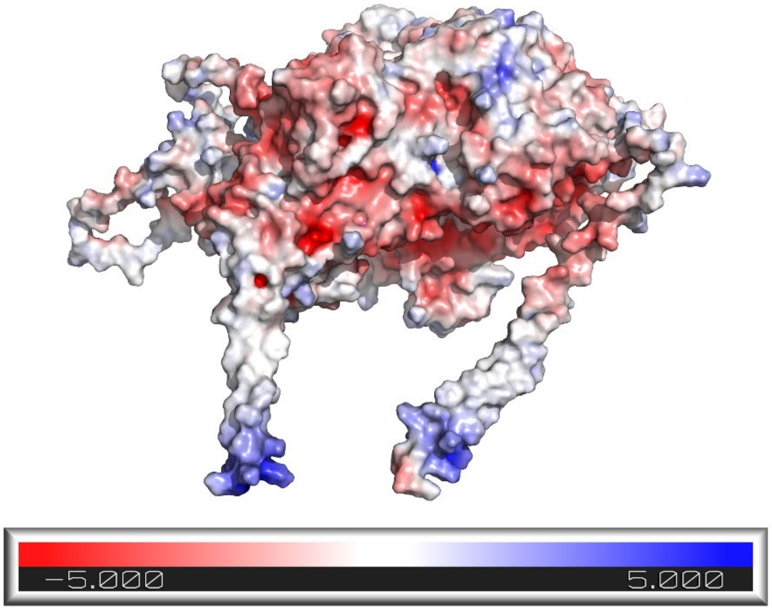

(D) 5’-
tggcgaatgggacgcgccctgtagcggcgcattaagcgcggcgggtgtggtggttacgcgcagcgtgaccgctacacttgccagcgccctagcgcccgctcctttcgctttc
ttcccttcctttctcgccacgttcgccggctttccccgtcaagctctaaatcgggggctccctttagggttccgatttagtgctttacggcacctcgaccccaaaaaacttgattagg
gtgatggttcacgtagtgggccatcgccctgatagacggtttttcgccctttgacgttggagtccacgttctttaatagtggactcttgttccaaactggaacaacactcaaccctat
ctcggtctattcttttgatttataagggattttgccgatttcggcctattggttaaaaaatgagctgatttaacaaaaatttaacgcgaattttaacaaaatattaacgtttacaatttca
ggtggcacttttcggggaaatgtgcgcggaacccctatttgtttatttttctaaatacattcaaatatgtatccgctcatgaattaattcttagaaaaactcatcgagcatcaaatga
aactgcaatttattcatatcaggattatcaataccatatttttgaaaaagccgtttctgtaatgaaggagaaaactcaccgaggcagttccataggatggcaagatcctggtatc
ggtctgcgattccgactcgtccaacatcaatacaacctattaatttcccctcgtcaaaaataaggttatcaagtgagaaatcaccatgagtgacgactgaatccggtgagaat
ggcaaaagtttatgcatttctttccagacttgttcaacaggccagccattacgctcgtcatcaaaatcactcgcatcaaccaaaccgttattcattcgtgattgcgcctgagcgag
acgaaatacgcgatcgctgttaaaaggacaattacaaacaggaatcgaatgcaaccggcgcaggaacactgccagcgcatcaacaatattttcacctgaatcaggatatt
cttctaatacctggaatgctgttttcccggggatcgcagtggtgagtaaccatgcatcatcaggagtacggataaaatgcttgatggtcggaagaggcataaattccgtcagcc
agtttagtctgaccatctcatctgtaacatcattggcaacgctacctttgccatgtttcagaaacaactctggcgcatcgggcttcccatacaatcgatagattgtcgcacctgatt
gcccgacattatcgcgagcccatttatacccatataaatcagcatccatgttggaatttaatcgcggcctagagcaagacgtttcccgttgaatatggctcataacaccccttgt
attactgtttatgtaagcagacagttttattgttcatgaccaaaatcccttaacgtgagttttcgttccactgagcgtcagaccccgtagaaaagatcaaaggatcttcttgagatcc
tttttttctgcgcgtaatctgctgcttgcaaacaaaaaaaccaccgctaccagcggtggtttgtttgccggatcaagagctaccaactctttttccgaaggtaactggcttcagcag
agcgcagataccaaatactgtccttctagtgtagccgtagttaggccaccacttcaagaactctgtagcaccgcctacatacctcgctctgctaatcctgttaccagtggctgct
gccagtggcgataagtcgtgtcttaccgggttggactcaagacgatagttaccggataaggcgcagcggtcgggctgaacggggggttcgtgcacacagcccagcttgga
gcgaacgacctacaccgaactgagatacctacagcgtgagctatgagaaagcgccacgcttcccgaagggagaaaggcggacaggtatccggtaagcggcagggtc
ggaacaggagagcgcacgagggagcttccagggggaaacgcctggtatctttatagtcctgtcgggtttcgccacctctgacttgagcgtcgatttttgtgatgctcgtcaggg
gggcggagcctatggaaaaacgccagcaacgcggcctttttacggttcctggccttttgctggccttttgctcacatgttctttcctgcgttatcccctgattctgtggataaccgtat
taccgcctttgagtgagctgataccgctcgccgcagccgaacgaccgagcgcagcgagtcagtgagcgaggaagcggaagagcgcctgatgcggtattttctccttacgc
atctgtgcggtatttcacaccgcatatatggtgcactctcagtacaatctgctctgatgccgcatagttaagccagtatacactccgctatcgctacgtgactgggtcatggctgc
gccccgacacccgccaacacccgctgacgcgccctgacgggcttgtctgctcccggcatccgcttacagacaagctgtgaccgtctccgggagctgcatgtgtcagaggtt
ttcaccgtcatcaccgaaacgcgcgaggcagctgcggtaaagctcatcagcgtggtcgtgaagcgattcacagatgtctgcctgttcatccgcgtccagctcgttgagtttctc
cagaagcgttaatgtctggcttctgataaagcgggccatgttaagggcggttttttcctgtttggtcactgatgcctccgtgtaagggggatttctgttcatgggggtaatgataccg
atgaaacgagagaggatgctcacgatacgggttactgatgatgaacatgcccggttactggaacgttgtgagggtaaacaactggcggtatggatgcggcgggaccaga
gaaaaatcactcagggtcaatgccagcgcttcgttaatacagatgtaggtgttccacagggtagccagcagcatcctgcgatgcagatccggaacataatggtgcagggc
gctgacttccgcgtttccagactttacgaaacacggaaaccgaagaccattcatgttgttgctcaggtcgcagacgttttgcagcagcagtcgcttcacgttcgctcgcgtatcg
gtgattcattctgctaaccagtaaggcaaccccgccagcctagccgggtcctcaacgacaggagcacgatcatgcgcacccgtgggccgccatgccggcgataatggc
ctgcttctcgccgaaacgtttggtggcgggaccagtgacgaaggcttgagcgagggcgtgcaagattccgaataccgcaagcgacaggccgatcatcgtcgcgctccag
cgaaagcggtcctcgccgaaaatgacccagagcgctgccggcacctgtcctacgagttgcatgataaagaagacagtcataagtgcggcgacgatagtcatgccccgc
gcccaccggaaggagctgactgggttgaaggctctcaagggcatcggtcgagatcccggtgcctaatgagtgagctaacttacattaattgcgttgcgctcactgcccgcttt
ccagtcgggaaacctgtcgtgccagctgcattaatgaatcggccaacgcgcggggagaggcggtttgcgtattgggcgccagggtggtttttcttttcaccagtgagacggg

caacagctgattgcccttcaccgcctggccctgagagagttgcagcaagcggtccacgctggtttgccccagcaggcgaaaatcctgtttgatggtggttaacggcgggata
taacatgagctgtcttcggtatcgtcgtatcccactaccgagatatccgcaccaacgcgcagcccggactcggtaatggcgcgcattgcgcccagcgccatctgatcgttgg
caaccagcatcgcagtgggaacgatgccctcattcagcatttgcatggtttgttgaaaaccggacatggcactccagtcgccttcccgttccgctatcggctgaatttgattgcg
agtgagatatttatgccagccagccagacgcagacgcgccgagacagaacttaatgggcccgctaacagcgcgatttgctggtgacccaatgcgaccagatgctccacg
cccagtcgcgtaccgtcttcatgggagaaaataatactgttgatgggtgtctggtcagagacatcaagaaataacgccggaacattagtgcaggcagcttccacagcaatg
gcatcctggtcatccagcggatagttaatgatcagcccactgacgcgttgcgcgagaagattgtgcaccgccgctttacaggcttcgacgccgcttcgttctaccatcgacac
caccacgctggcacccagttgatcggcgcgagatttaatcgccgcgacaatttgcgacggcgcgtgcagggccagactggaggtggcaacgccaatcagcaacgactg
tttgcccgccagttgttgtgccacgcggttgggaatgtaattcagctccgccatcgccgcttccactttttcccgcgttttcgcagaaacgtggctggcctggttcaccacgcggg
aaacggtctgataagagacaccggcatactctgcgacatcgtataacgttactggtttcacattcaccaccctgaattgactctcttccgggcgctatcatgccataccgcgaa
aggttttgcgccattcgatggtgtccgggatctcgacgctctcccttatgcgactcctgcattaggaagcagcccagtagtaggttgaggccgttgagcaccgccgccgcaag
gaatggtgcatgcaaggagatggcgcccaacagtcccccggccacggggcctgccaccatacccacgccgaaacaagcgctcatgagcccgaagtggcgagcccg
atcttccccatcggtgatgtcggcgatataggcgccagcaaccgcacctgtggcgccggtgatgccggccacgatgcgtccggcgtagaggatcgagatctcgatcccgc
gaaattaatacgactcactataggggaattgtgagcggataacaattcccctctagaaataattttgtttaactttaaga**aggaga**tatacccat**atggcaacaaaaatagct**
ttcgtaaattttcaaacgatccagcaaggttctatcagcaaagccaacgataatagctttatcaagctgagcgaagtttctctcgataacttagaccgtctgacga
gctatgatatggtgttcatcaatggcatgggcttgcgtattgtggaagaacagcgtcaacaaatccaacaggccgccgacaagggtattccggtttacaccag
catggcgactaatccggcgaacaatatttgcaatctggatagcatccagcagaaccttatccgtggttacctgtccaacggcggcaagaccaactaccgtaac
atgctcaactatatccgtaaagcaatcgatggtaaggcctctgccgtgccggaggtagaggacccgattgaacgtccgtccgatatgttgtaccatgcaggca
tcagcaacccggatgatgagcaagagttcctgaccgttgcggactacgaaaagtttatgcaggaaaacaatctgtacaaagagggtgcgagaaagattatga
ttaccggccagatggcagacgcgaccgatcttattaaagcacttgagaacgcaggttataacgtctatccggtccaaagcatgacccgttttatgtccttcattga
agaggtgcagccggacgcggtgatcaatatggcacatggtcggatgggggataagatggtcgattatttgaaagcgcgcaatatcctgttgttcgcaccgctg
accattaacagtctggtggatgagtgggaaaacgatccgatgggtatgagcggcggttttatgagccagagcatcgttactccggagatcgacggcgcaatc
cgtccgtttgcactgttcgcgcagtatgaggataaagagggtctacgccacagctacgcggtgccggaacgtttgaaaacttttgtcagcaccattgacaacta
cttgaacttgaagaccaagccgaactttgaaaagaaagttgcgatttattactacaagggtccgggtcaaaatgctctgacggcagcgggcatggaggttgta
ccgagcctctacaacctgttgctgcgtatgaagcaggaaggttataacattagcggactgcctgcaaatgcgcaagaactgggtaagatgattcaggctcagg
gcgctgtgtttaacgcatacgcggaaggtgcgttcaacgacttcatgcagaatggccacccagagctgattaccaaagagcagtatgagtcttgggttaaaga
gagcttgcgcccagagaagtaccaggaagttgttgatgcgtttggagaatttccgggtaactacatggtgacaccagacggcaaattaggcatcgcacgtctg
caatttggcaacgtggtgctgctgccgcaaaacgcggccggttcgggcgacaacagcttccaggttgttcatggtacagacatggctccgccacacacctac
attgcatcgtacctgtggatgcagcatggcttcaaagcggacgcgctgatacactttggtacccatggcagcctcgagttcaccccgagaaagcaagttgcac
tttgcagcaacgactggccggatcgtttggttggggcagtcccgcactattacctttacagcatcggtaatgttggtgaaggcatgatggccaagcgccgttcgt
acgcgaccctgcagagctacttgaccccgccttttctggagagcagcgtgcgtggtatctatcgggaattaatggaaaaaatcaaaatttataacaactcccag
aaagcgaacaaagatcaagaaagcttggcggtcaagaccctgacggttaaaatgggtattcaccgtgatctaggtctggattctatggctaacaaaccgtata
ccgaagacgaaatcgctcgcgttgagaacttcgcggaagaattggcgaccgagaagatcaccggtcaattgtacaccatgggcgtgccgtacgaaccgga
gcgcattacgtcgagcgtgtatgccatggcgaccgagccgattgcgtacagcctcttcgcactggataagcagcgtggcaaagcgaccgaatcggccgaa
aagcaccgctctgtttttacccagcaatacctgatgccggcacgcctcttggttgagcgtctgatggctaacccgagcctcgccacggacgaactgatatgcc
ataccgctggcattaccccgcaggagctggcgaaagcccgtcaaatcgaagctgagcgcaatgcgccaaaaggaatgatggcgatgatgatggccgcgg
cagcgaaaaaggaccaggcagataacgagccgagcggcaacggtcacccggcgtccgcgaagatggaaaaaggccctcacggcaagatgccggccg
gcatgaaggaggcaatgaagaaaatgggcgctaatatggatccggaaaaggcgatggaaatggccaaaagcatgggtgctagcccagaagcgttgaaaa
aaatggaagccagcatgaaagcgaataaggacacctccactgatgcgtccggcaagccggctatggctggcaaaacggagaagccacaaggtatgtctg
caatgatggcggctatgggtaaggcgccaaaagaatattccaaagaagaggtggagttcgctctggctgtggccgaggttgaacgtaccattaaaaatgttgg
taattacaagaatgcgttgctgacgagcccggaggaggaattgtcctctctgatgaatgctcttaagggcggctacacggcgccgaccccaggtggtgaccc
tatcgcgaacccgaacaccctgccgaccggtagaaacatgtacgcgatcaacgccgaagcgacccccaccgagtcagcgtgggaaaagggcattgccct
ggcgaagcaaaccattgaccgctataagcaacgtcacaatgatagcatcccgcgcaaggtgagctacaccctgtggagctcggagttcatcgagacaggc
ggtgcaacgatcgcccaggttctgtacatgctgggggtggagccggttcgtgatgcgttcggccgtgtgtccgatctgaaactgattccgtctaccgagctgg
gtcgtccgagaattgacgtggtggtccagactagcggacagctgcgtgacctggccgcgtctcgtttatttctgatcaaccgtgcagtggaaatggcagctgc
ggcgaaggacgacaaatacgaaaaccaggtggcgagcagcgtcatcgaggctgaacgtgtcctgaccgaaaaaggcctgtccccgaaagatgcgcgcg
aaatttccacctttcgtgttttcggtggtgcgaacggtatgtatggtaccggtatccaagagatggttgagtccggcgaccgctgggaaaatgagagcgaaatc
gccgacacatacctgaacaacatgggtgcatattacggcagcgagaagaactgggaagtgtttcagaagttcgccttcgaagcggcgctgactcgcacgga
cgttgtggtgcaaccgcgtcaatctaatacctggggtgcgctgagcctggaccatgtgtacgagttcatgggtggaatgaacttggcggtgcgcaatgtcacc
ggcaaggacccggatgcctatttaagcgactatagaaaccgcaaccacatgaagatgcaagagcttaaagaggcagttggcgttgagtcccgtaccaccatt
ctgaatccgacctatatcaaagagaagatgaaaggtggtgcgtcctctgcatcagaattcgctgaggttattacgaacacgtatggctggaatgttatgaaacc
ggctgcaatcgataaagagctgtgggataacatctataacgtgtatgtgaaagacgagttaaaccttggcgttaaacaatacttcgagcagcagaacccggct
gctttggaggaaatgaccgcggtgatgctggaaagcgcccgtaaaggtctgtggcaggcctccgaggaacaagtggcagagctgagcaaactgcataccg
aaatcgttaatacctatcgtccgtcttgtagcggcttcgtttgtgataatgcgaaactgcgtgactttatcgccagcaaggcggacgctcagactgcgacgcagt
acaaggaaaacatctcgaagattagataatgactcgagcaccaccaccaccaccactgagatccggctgctaacaaagcccgaaaggaagctgagttggctgctgc
caccgctgagcaataactagcataaccccttggggcctctaaacgggtcttgaggggttttttgctgaaaggaggaactatatccggat-3’
(E)
MATKIAFVNFQTIQQGSISKANDNSFIKLSEVSLDNLDRLTSYDMVFINGMGLRIVEEQRQQIQQAADKGIPVYTSMATNPA
NNICNLDSIQQNLIRGYLSNGGKTNYRNMLNYIRKAIDGKASAVPEVEDPIERPSDMLYHAGISNPDDEQEFLTVADYEKFM

QENNLYKEGARKIMITGQMADATDLIKALENAGYNVYPVQSMTRFMSFIEEVQPDAVINMAHGRMGDKMVDYLKARNILLF
APLTINSLVDEWENDPMGMSGGFMSQSIVTPEIDGAIRPFALFAQYEDKEGLRHSYAVPERLKTFVSTIDNYLNLKTKPNFE
KKVAIYYYKGPGQNALTAAGMEVVPSLYNLLLRMKQEGYNISGLPANAQELGKMIQAQGAVFNAYAEGAFNDFMQNGHP
ELITKEQYESWVKESLRPEKYQEVVDAFGEFPGNYMVTPDGKLGIARLQFGNVVLLPQNAAGSGDNSFQVVHGTDMAPP
HTYIASYLWMQHGFKADALIHFGTHGSLEFTPRKQVALCSNDWPDRLVGAVPHYYLYSIGNVGEGMMAKRRSYATLQSYL
TPPFLESSVRGIYRELMEKIKIYNNSQKANKDQESLAVKTLTVKMGIHRDLGLDSMANKPYTEDEIARVENFAEELATEKITG
QLYTMGVPYEPERITSSVYAMATEPIAYSLFALDKQRGKATESAEKHRSVFTQQYLMPARLLVERLMANPSLATDELICHTA
GITPQELAKARQIEAERNAPKGMMAMMMAAAAKKDQADNEPSGNGHPASAKMEKGPHGKMPAGMKEAMKKMGANMD
PEKAMEMAKSMGASPEALKKMEASMKANKDTSTDASGKPAMAGKTEKPQGMSAMMAAMGKAPKEYSKEEVEFALAVA
EVERTIKNVGNYKNALLTSPEEELSSLMNALKGGYTAPTPGGDPIANPNTLPTGRNMYAINAEATPTESAWEKGIALAKQTI
DRYKQRHNDSIPRKVSYTLWSSEFIETGGATIAQVLYMLGVEPVRDAFGRVSDLKLIPSTELGRPRIDVVVQTSGQLRDLAA
SRLFLINRAVEMAAAAKDDKYENQVASSVIEAERVLTEKGLSPKDAREISTFRVFGGANGMYGTGIQEMVESGDRWENES
EIADTYLNNMGAYYGSEKNWEVFQKFAFEAALTRTDVVVQPRQSNTWGALSLDHVYEFMGGMNLAVRNVTGKDPDAYL
SDYRNRNHMKMQELKEAVGVESRTTILNPTYIKEKMKGGASSASEFAEVITNTYGWNVMKPAAIDKELWDNIYNVYVKDEL
NLGVKQYFEQQNPAALEEMTAVMLESARKGLWQASEEQVAELSKLHTEIVNTYRPSCSGFVCDNAKLRDFIASKADAQTA
TQYKENISKIR

Appendix Figure S6. Annotated protein sequence of HmuS and AlphaFold2-predicted structure used in designing the expression construct; Annotated DNA sequence of 9373 nucleotide expression construct used to generate HmuS. (A) The full-length 1463 coding sequence (RefSeq: WP_008765019.1) was truncated to eliminate predicted single-transmembrane spanning alpha helices (prediction using Phobius, https://phobius.sbc.su.se/cgi-bin/predict.pl) at the N- and C-termini (residues 1-29 and 1406-1463, highlighted in green; N- and C-termini are labeled). The remaining 1377 amino acid sequence (orange, underlined/bold-italics) was reverse-translated by GenScript and used to generate a synthetic gene for expression in *E. coli* strain BL21DE3.

(B) AlphaFold2 structure of the full-length native HmuS, color-coded to match the expressed/unexpressed regions delineated in (A). The N-terminal His tag is not shown. A series of residues predicted to form a poorly structured coil were omitted at the C-terminus to avoid aggregation of the expressed protein.

(C) Electrostatic potential surface of the AlphaFold structure was generated using the Adaptive Poisson-Boltzmann Solver (APBS) tool in Pymol. Negative surfaces are in red, positive in blue, and neutral/nonpolar in white. Contour levels are +/- 5 kT/e. Note the pair of hydrophobic helices predicted to span the membrane, and the large stretch of negative charge on the protein's surface between them.

(D) The full-length gene encoding the HmuS protein from *Bacteroides thetaiotaomicron* (strain VPI 5482, ATCC 29148, locus numbers BT0495-BT0494, protein accession: WP_022471467.1) was codon-optimized for expression in *E. coli* and synthetically introduced between the NdeI/XhoI sites of the sense strand of vector pET28a(+) by GenScript. The ribosome binding site (RBS) is shown in red. The expressed gene sequence encoding HmuS is shown in **orange**. The sequence in **violet** at the 3' end of the gene corresponds to two STOP codons inserted into the construct. Sequences encoding the Lac repressor protein and an enzyme providing kanamycin resistance (KanR) are encoded in the opposite direction from the *hmuS* gene, on the minus strand (not shown).

(E) FASTA sequence of the expressed protein (1377 amino acids), translated from part (D). A methionine was added at the N-terminus, corresponding to the start codon.

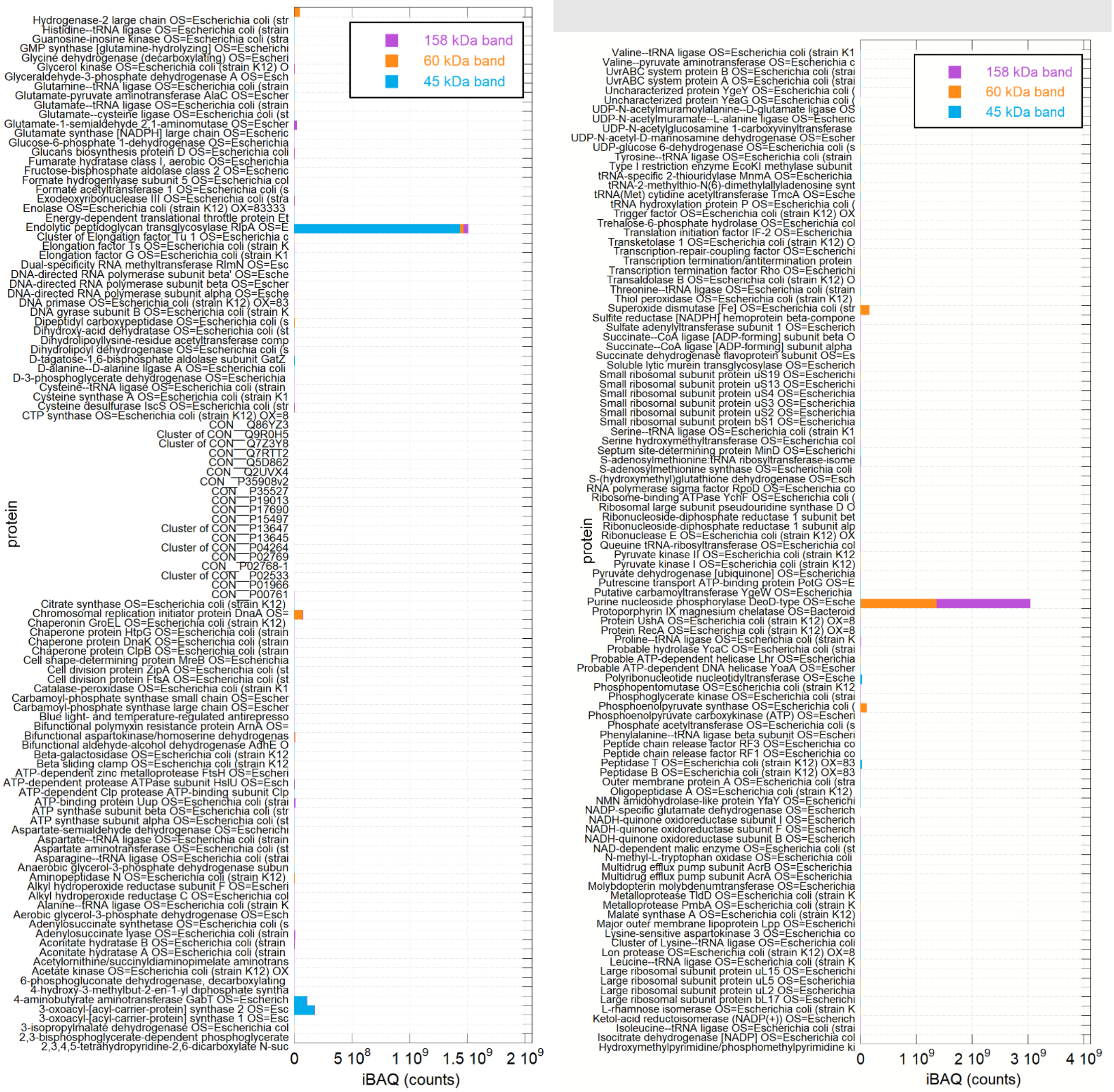

Appendix Figure S7. Mass spectrometric analysis of heterologously expressed HmuS confirms its identity and integrity and shows its major contaminant is principally composed of HmuS-derived peptides. HmuS was expressed and purified by IEC as described in the text, and the IEC-purified protein analyzed by SDS-PAGE (12% acrylamide). Bands corresponding to purified HmuS (160 kDa), its major contaminant at 60 kDa, and a contaminant that was later successfully removed by SEC (45 kDa, control) were excised from the gel, proteolyzed with trypsin, and eluted from the gel matrix as described in the Supplementary Methods section above. The peptides from each band were injected onto a UPLC in-line with an Orbitrap mass spectrometer for MS identification of peptides and MS/MS analysis of their sequence. Peptides were mapped onto the *B theta* HmuS sequence or sequences from the *E. coli* host, and proteins were quantified using the iBAQ approach (plotted as superimposed columns above for the top 220 protein hits). Analysis of the 158 kDa band showed HmuS (“protoporphyrin IX Mg^{2+} chelatase,” indicated with an arrow on the plotted proteins and their relative amounts above) as the top hit. 91 peptides were detected in 1222 total spectra (4.7% of the total), representing 75% coverage (1073/1427 amino acids) of the HmuS sequence. Peptides were detected from across the entire sequence, with no systematic gaps. The iBAQ score relative to the summed iBAQ scores (riBAQ) indicated 73% of the band consisted of HmuS. (b) Analysis of the 60 kDa band indicated 84 HmuS peptides detected in 783 spectra, representing 70% coverage of the HmuS sequence. The riBAQ(HmuS) for this band was 37%. Complete proteomics data results are available in Dataset EV1.

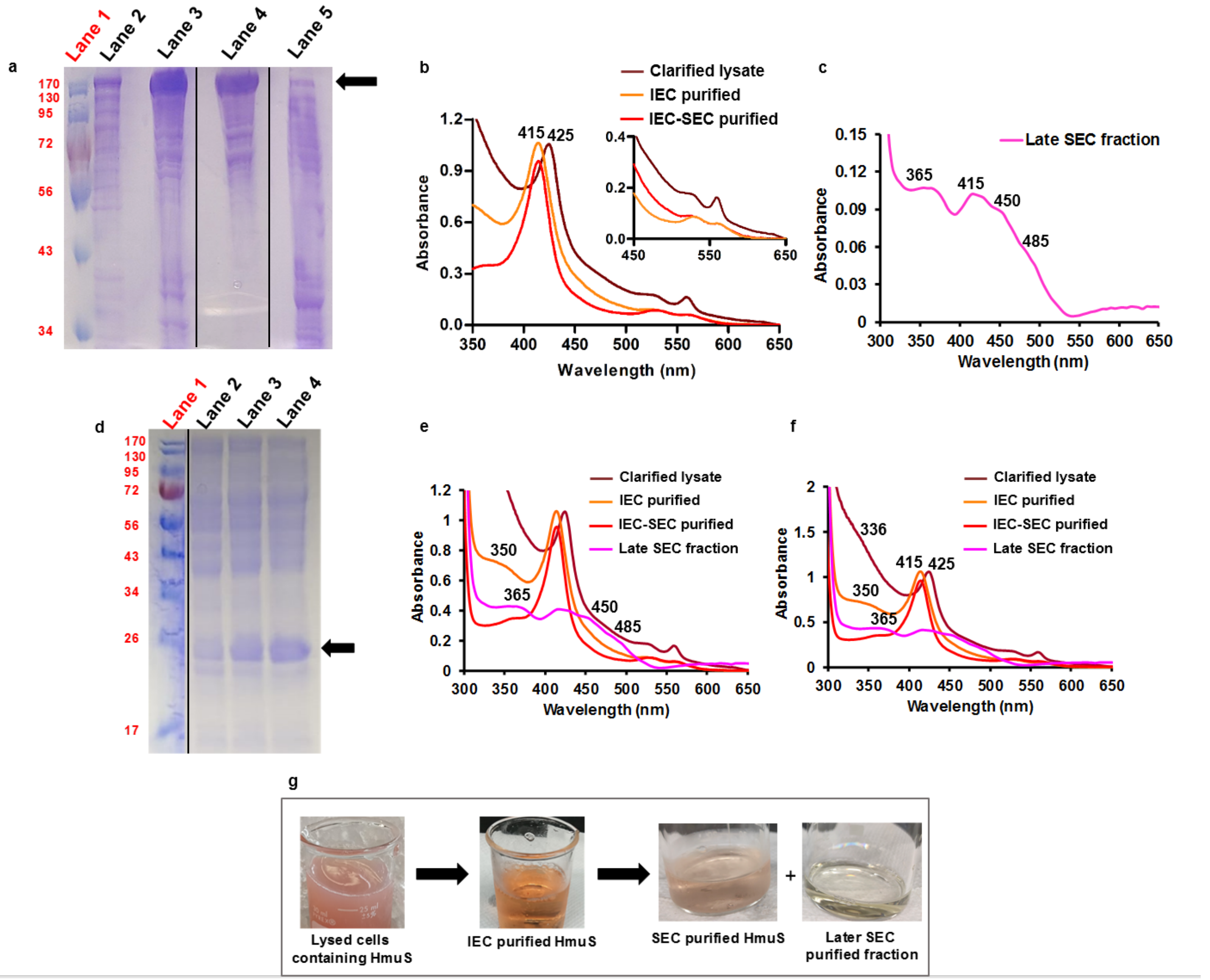

Appendix Figure S8. Steps in the purification of recombinant HmuS illustrated by SDS-PAGE, UV/visible absorbance, and fraction color. (a) 12% SDS-PAGE analyses of heterologously expressed HmuS in the soluble portion of the host/*E. coli* lysate (centrifuge-clarified lysate) (lane 2, 20 µg), following purification by ion exchange (lane 3, 30 µg), and after further purification by size exclusion chromatography (lane 4, 30 µg). [Note: this image is reproduced in part from Figure 3b. Lanes 1-4 correspond to lanes marked M, 1, 2, and 3, respectively.] Lane 5 contains pooled, low-molecular weight fractions that eluted after the HmuS (late SEC fraction) (90 µg total protein). The molecular weight size marker was loaded in lane 1. The arrow indicates the 160 kDa HmuS protein. Black lines indicate where pieces of the same gel were digitally excised in generating the image. (b) UV/visible absorbance spectra of heterologously expressed HmuS were measured for the same fractions as in (a), but with approximately equal [heme] in each fraction (7.5-9.5 µM as measured by the pyridine hemochromagen assay). The clarified *E. coli* lysate spectrum is shown in rust (22 mg mL^{-1} total protein, roughly 10% HmuS based on (a)); following purification by ion exchange (orange line, 15 mg mL^{-1} total protein, >30% HmuS); and after further purification by size exclusion chromatography (red line, 10 mg mL^{-1} total protein, ≥70% HmuS). As-isolated HmuS contains substoichiometric, reduced heme. The heme oxidized and partly dissociated from the protein during purification. (c) UV/visible absorbance spectrum and (d) 12% SDS-PAGE of the late-eluting, yellow SEC fraction (16 mg mL^{-1} total protein). The arrow indicates a prominent 26 kDa contaminant protein. The molecular marker was loaded in lane 1. Black line indicates where pieces of the same gel were digitally excised in generating the image. The total protein from lane 4 was analyzed using MS-proteomics (see main text and Dataset EV2.) Panels (e) and (f) show the same data as in panels (b) and (c) overlaid. The spectrum from panel (c) is shown on an expanded scale (x 4) to illustrate its peak positions. Panel (g) shows digital images of purification fractions, illustrating their colors.

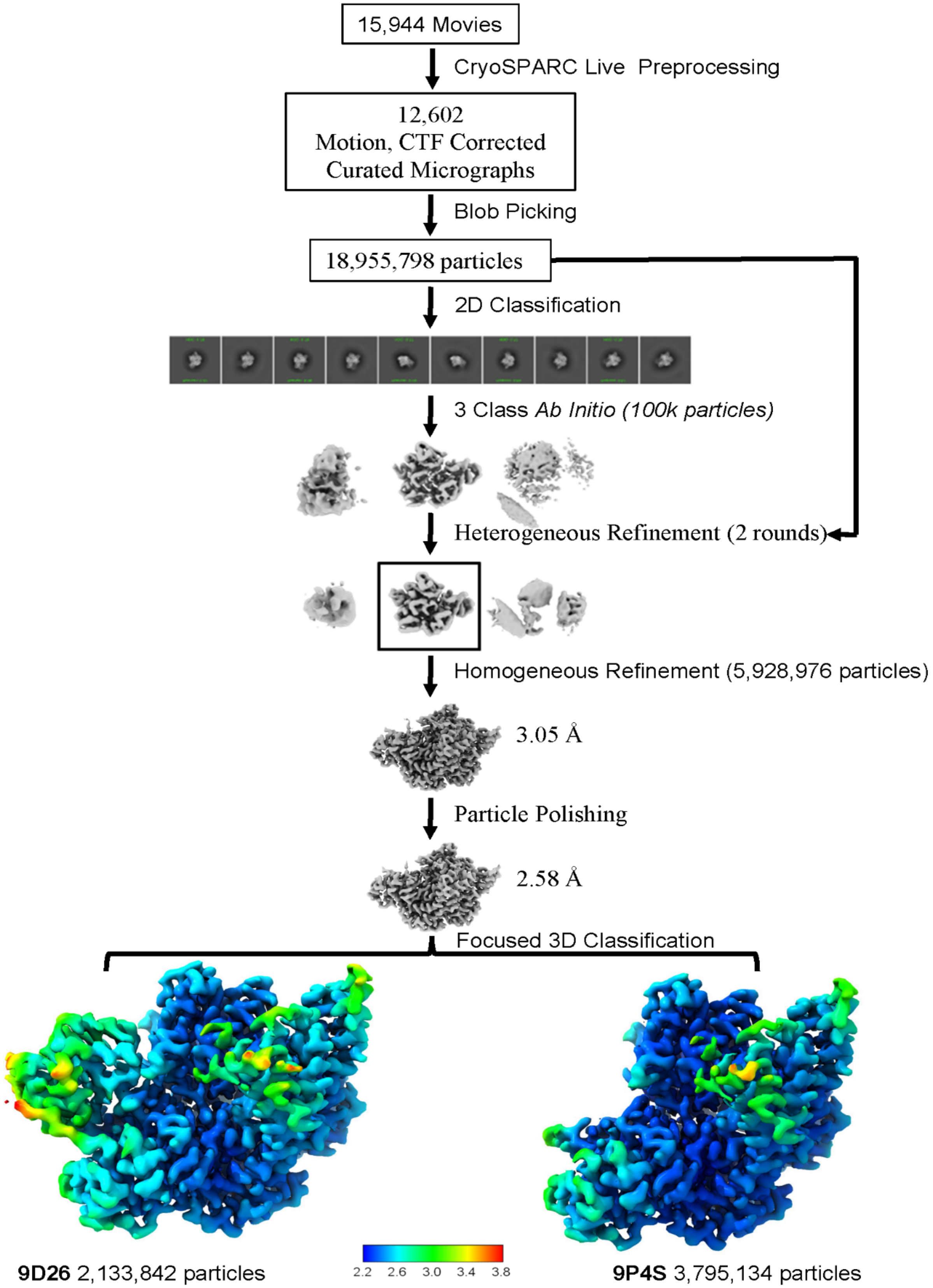

Appendix Figure S9. Single particle workflow. See Methods for details. Local resolution maps are color contoured from 2.2 Å (dark blue) to 3.8 Å (red) resolution. Particles are oriented with the head domain in 9D26 on the far left, and absent from 9P4S.

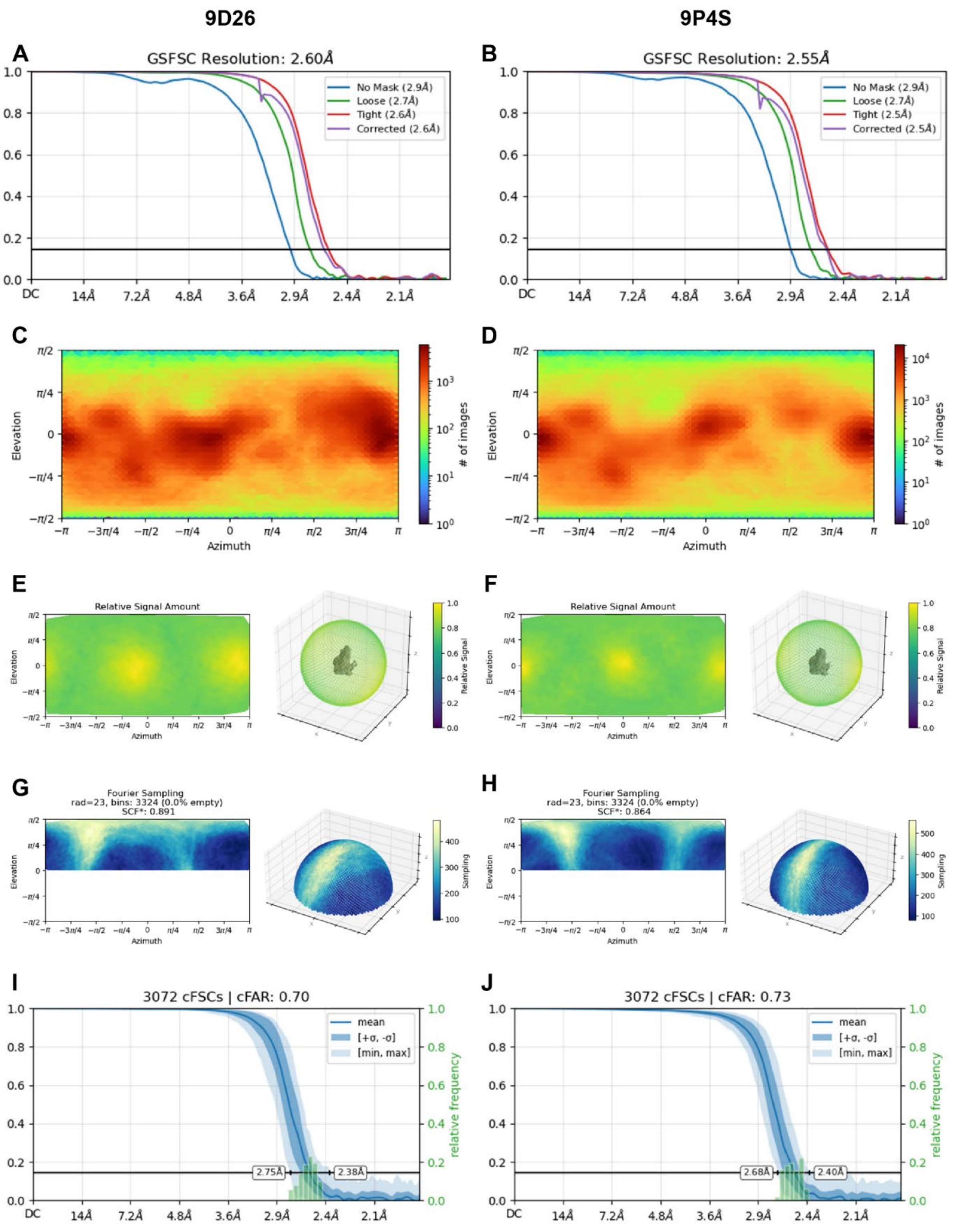

Appendix Figure S10. Resolution and Orientation Diagnostics. A and B) Gold Standard FSC curves for 9D26 (left column) and 9D4S (right column). C and D) The CryoSPARC Viewing Direction Distribution plot, showing nearly complete coverage along the azimuthal direction. E and F) Relative signal diagnostics show signal between 0.75 (lime green) and 1 (yellow) in all directions. G and H) The Sampling Compensation Factor (SCF) for the 9D26 and 9D4S data sets are 0.891 and 0.864, respectively. Values above 0.81 generally indicate good, though not necessarily isotropic signal content (Baldwin and Lyumkis 2021). I and J) The conical FSC area ratio for 9D26 and 9D4S are 0.70 and 0.73, respectively, where 0 indicates severe orientation bias, and 1 indicates no bias. CryoSPARC documentation suggests a cFAR of 0.5 "serves as a reasonable threshold for the presence, or lack thereof, of preferred orientation".

β I-1 α I-1 β I-2

1 MKKKSKIILGGCIVVAALIGLSVWNTWFSATKIAFVNFQTIQQGSISKANDNSFIKLSEVSLDNLDRLTS 70

β I-3 α I-2 α I-3 β I-4 α I-4 α I-5

71 YDMVFINGMGLRIVEEQRQQIQQAADKGIPVYTSMATNPANNICNLDSIQQNLIRGYLSNGGKTNYRNML 140

α I-6 β II-1 β II-2 α II-1 β II-3

141 NYIRKAIDGKASAVPEVEDPIERPSDMLYHAGISNPDDEQEFLTVADYEKFMQENNLYKEGARKIMITGQ 210

α II-2 β II-4 α II-3 β II-5 α II-4 β II-6 β II-7

211 MADATDLIKALENAGYNVYPVQSMTRFMSFIEEVQPDAVINMAHGRMGDKMVDYLKARNILLFAPLTINS 280

α II-5 α II-6 β II-8 β II-9 α II-7

281 LVDEWENDPMGMSGGFMSQSIVTPEIDGAIRPFALFAQYEDKEGLRHSYAVPERLKTFVSTIDNYLNLKT 350

β III-1 α III-1 α III-2

351 KPNFEKKVAIYYYKGPGQNALTAAGMEVVPSLYNLLLRMKQEGYNISGLPANAQELGKMIQAQGAVFNAY 420

α III-3 βIII-2 α III-4 α III-5 βIII-3 βIII-4 βIII-5

421 AEGAFNDFMQNGHPELITKEQYESWVKESLRPEKYQEVVDAFGEFPGNYMVTPDGKLGIARLQFGNVVLL 490

α III-6 βIII-6 α III-7

491 PQNAAGSGDNSFQVVHGTDMAPPHTYIASYLWMQHGFKADALIHFGTHGSLEFTPRKQVALCSNDWPDRL 560

β III-7 α III-8 βIII-8 βIII-9 α IV-1 α IV-2

561 VGAVPHYYLYSIGNVGEGMMAKRRSYATLQSYLTPPFLESSVRGIYRELMEKIKIYNNSQKANKDQESLA 630

α IV-2 α IV-3 α IV-4 βIV-1 α IV-5

631 VKTLTVKMGIHRDLGLDSMANKPYTEDEIARVENFAEELATEKITGQLYTMGVPYEPERITSSVYAMATE 700

α IV-6 α IV-7 α IV-8 α IV-9 α IV-10 α IV-11

701 PIAYSLFALDKQRGKATESAEKHRSVFTQQYLMPARLLVERLMANPSLATDELICHTAGITPQELAKARQ 770

α IV-11

771 IEAERNAPKGMMAMMMAAAAKKDQADNEPSGNGHPASAKMEKGPHGKMPAGMKEAMKKMGANMDPEKAME 840

α IV-12

841 MAKSMGASPEALKKMEASMKANKDTSTDASGKPAMAGKTEKPQGMSAMMAAMGKAPKEYSKEEVEFALAV 910

α IV-12 α IV-13, α III-9

911 AEVERTIKNVGNYKNALLTSPEEELSSLMNALKGGYTAPTPGGDPIANPNTLPTGRNMYAINAEATPTES 980

α V-1 β V-1 α V-2 α V-3 β V-2 β V-3

981 AWEKGIALAKQTIDRYKQRHNDSIPRKVSYTLWSSEFIETGGATIAQVLYMLGVEPVRDAFGRVSDLKLI 1050

α V-4 β V-4 α V-5 α V-6 α V-7 αV-8

1051 PSTELGRPRIDVVVQTSGQLRDLAASRLFLINRAVEMAAAAKDDKYENQVASSVIEAERVLTEKGLSPKD 1120

α V-8 β V-5 α V-9 α V-10 α V-11

1121 AREISTFRVFGGANGMYGTGIQEMVESGDRWENESEIADTYLNNMGAYYGSEKNWEVFQKFAFEAALTRT 1190

β V-6 α V-12 β V-7 β V-8 α VI-1

1191 DVVVQPRQSNTWGALSLDHVYEFMGGMNLAVRNVTGKDPDAYLSDYRNRNHMKMQELKEAVGVESRTTIL 1260

α VI-2 α VI-3 α VI-4 α VI-5 α VI-6

1261 NPTYIKEKMKGGASSASEFAEVITNTYGWNVMKPAAIDKELWDNIYNVYVKDELNLGVKQYFEQQNPAAL 1330

α VI-6 α VI-7 α VI-8 α VI-9

1331 EEMTAVMLESARKGLWQASEEQVAELSKLHTEIVNTYRPSCSGFVCDNAKLRDFIASKADAQTATQYKEN 1400

1401 ISKIREAKASGSNKGVVMKKEEMNQTAENQTNTLSNVAVGIAVIIVILALILFVRKRRKSSQM 1463

Appendix Figure S11. Secondary structure description. The unexpressed sequences at the N- and C-termini are highlighted in gray, and the MRI is highlighted in green. Secondary structure elements from the cryo-EM single particle structure are indicated above the *B. theta* HmuS sequence. Domain boundaries are: Domain I 30-162; Domain II 163-350; Domain III 351-594; Domain IV 595-779 and 899-930; MRI 780-898; Domain V 972-1245; Domain VI 1246-1405.

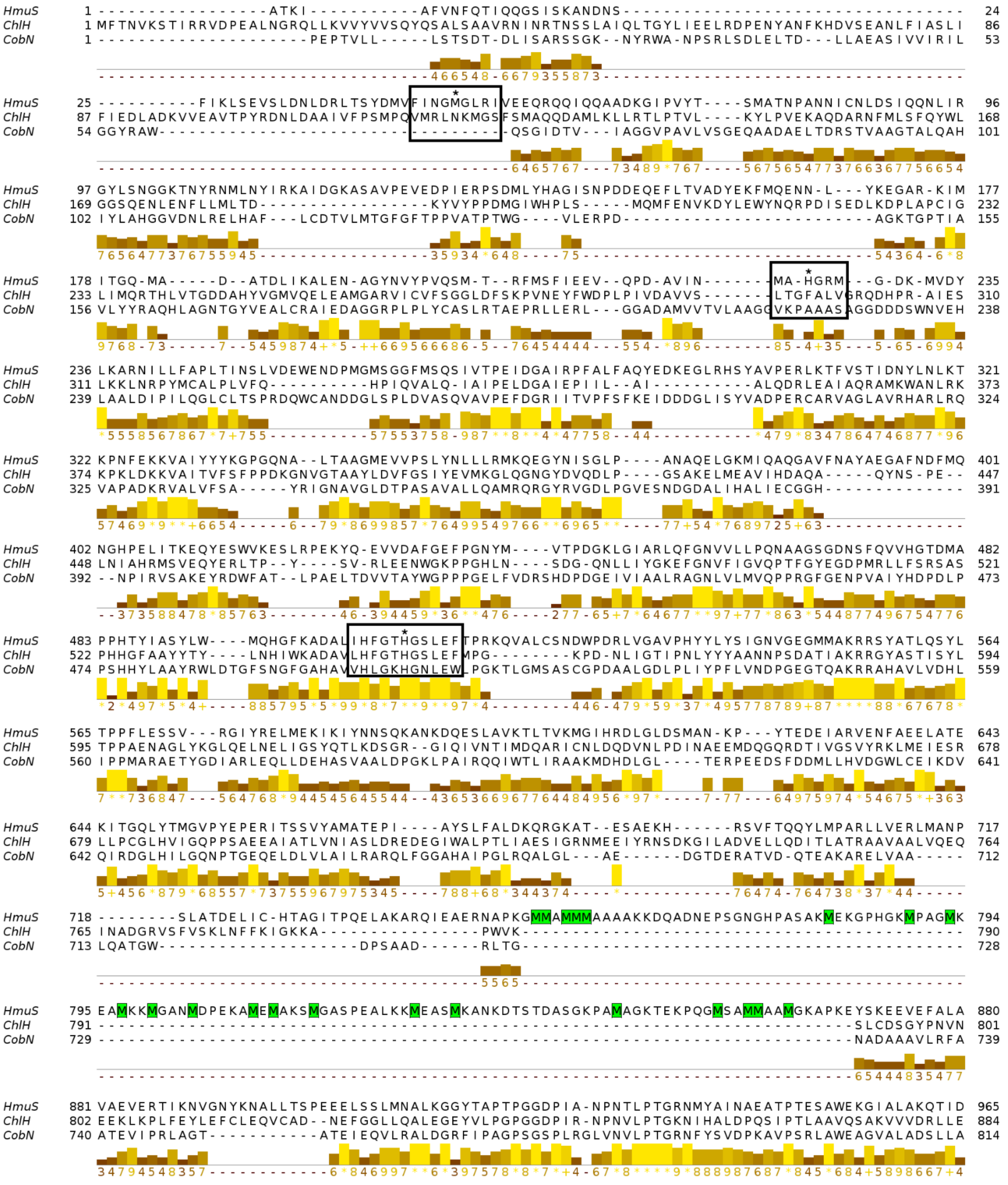
HmuS 1 -----------------ATKI-----------AFVNFQTIQQGSISKANDNS---------------------------------- 24
ChlH 1 MFTNVKSTIRRVDPEALNGRQLLKVVYVVSQYQSALSAAVRNINRTNSSLAIQLTGYLIEELRDPENYANFKHDVSEANLFIASLI 86
CobN 1 ---------------------PEPTVLL-----LSTSDT-DLISARSSGK--NYRWA-NPSRLSDLELTD---LLAEASIVVIRIL 53
---------------------------------466548-6679355873------------------------------------
HmuS 25 ----------FIKLSEVSLDNLDRLTSYDMVFINGMGLRIVEEQRQQIQQAADKGIPVYT----SMATNPANNICNLDSIQQNLIR 96
ChlH 87 FIEDLADKVVEAVTPYRDNLDAAIVFPSMPQVMRLNKMGSFSMAQQDAMLKLLRTLPTVL----KYLPVEKAQDARNFMLSFQYWL 168
CobN 54 GGYRAW-----------------------------------QSGIDTV---IAGGVPAVLVSGEQAADAELTDRSTVAAGTALQAH 101
---6465767---73489*767----5675654773766367756654
HmuS 97 GYLSNGGKTNYRNMLNYIRKAIDGKASAVPEVEDPIERPSDMLYHAGISNPDDEQEFLTVADYEKFMQENN-L---YKEGAR-KIM 177
ChlH 169 GGSQENLENFLLMLTD-----------------KYVYPPDMGIWHPLS-----MQMFENVKDYLEWYNQRPDISEDLKDPLAPCIG 232
CobN 102 IYLAHGGVDNLRELHAF---LCDTVLMTGFGFTPPVATPTWG----VLERPD-------------------------AGKTGPTIA 155
7656477376755945-----------------35934*648----75-----------------------------54364-6*8
HmuS 178 ITGQ-MA-----D--ATDLIKALEN-AGYNVYPVQSM-T--RFMSFIEEV--QPD-AVIN-------MA-HGRM---G-DK-MVDY 235
ChlH 233 LIMQRTHLVTGDDAHYVGMVQELEAMGARVICVFSGGLDFSKPVNEYFWDPLPIVDAVVS-------LTGFALVGRQDHPR-AIES 310
CobN 156 VLYYRAQHLAGNTGYVEALCRAIEDAGGRPLPLYCASLRTAEPRLLERL---GGADAMVVTVLAAGGVKPAAASAGGDDDSWNVEH 238
9768-73-----7--5459874+*5-++669566686-5--76454444---554-*896-------85-4+35---5-65-6994
HmuS 236 LKARNILLFAPLTINSLVDEWENDPMGMSGGFMSQSIVTPEIDGAIRPFALFAQYEDKEGLRHSYAVPERLKTFVSTIDNYLNLKT 321
ChlH 311 LKKLNRPYMCALPLVFQ----------HPIQVALQ-IAIPELDGAIEPIIL--AI----------ALQDRLEAIAQRAMKWANLRK 373
CobN 239 LAALDIPILQGLCLTSPRDQWCANDDGLSPLDVASQVAVPEFDGRIITVPFSFKEIDDDGLISYVADPERCARVAGLAVRHARLRQ 324
*5558567867*7+755----------57553758-987**8**4*47758--44----------*479*8347864746877*96
HmuS 322 KPNFEKKVAIYYYKGPGQNA--LTAAGMEVVPSLYNLLLRMKQEGYNISGLP----ANAQELGKMIQAQGAVFNAYAEGAFNDFMQ 401
ChlH 374 KPKLDKKVAITVFSFPPDKGNVGTAAYLDVFGSIYEVMKGLQGNGYDVQDLP----GSAKELMEAVIHDAQA-----QYNS-PE-- 447
CobN 325 VAPADKRVALVFSA-----YRIGNAVGLDTPASAVALLQAMRQRGYRVGDLPGVESNDGDALIHALIECGGH-------------- 391
57469*9**+6654-----6--79*8699857*76499549766**6965**----77+54*7689725+63--------------
HmuS 402 NGHPELITKEQYESWVKESLRPEKYQ-EVVDAFGEFPGNYM----VTPDGKLGIARLQFGNVVLLPQNAAGSGDNSFQVVHGTDMA 482
ChlH 448 LNIAHRMSVEQYERLTP--Y----SV-RLEENWGKPPGHLN----SDG-QNLLIYGKEFGNVFIGVQPTFGYEGDPMRLLFSRSAS 521
CobN 392 --NPIRVSAKEYRDWFAT--LPAELTDVVTAYWGPPPGELFVDRSHDPDGEIVIAALRAGNLVLMVQPPRGFGENPVAIYHDPDLP 473
--373588478*85763-------46-394459*36**476----277-65+7*64677**97+77*863*544877496754776
HmuS 483 PPHTYIASYLW---MQHGFKADALIHFGTHGSLEFTPRKQVALCSNDWPDRLVGAVPHYYLYSIGNVGEGMMAKRRSYATLQSYL 564
ChlH 522 PHHGFAAYYTY----LNHIWKADAVLHFGTHGSLEFMPG--------KPD-NLIGTIPNLYYYAANNPSDATIAKRRGYASTISYL 594
CobN 474 PSHHYLAAYRWLDTGFSNGFGAHAVVHLGKHGNLEWLPGKTLGMSASCGPDAALGDLPLIYPFLVNDPGEGTQAKRRAHAVLVDHL 559
*2*497*5*4+----885795*5*99*8*7**9**97*4--------446-479*59*37*495778789+87****88*67678*
HmuS 565 TPPFLESSV---RGIYRELMEKIKIYNNSQKANKDQESLAVKTLTVKMGIHRDLGLDSMAN-KP---YTEDEIARVENFAEELATE 643
ChlH 595 TPPAENAGLYKGLQELNELIGSYQTLKDSGR--GIQIVNTIMDQARICNLDQDVNLPDINAEEMDQGQRDTIVGSVYRKLMEIESR 678
CobN 560 IPPMARAETYGDIARLEQLLDEHASVAALDPGKLPAIRQQIWTLIRAAKMDHDLGL----TERPEEDSFDDMLLHVDGWLCEIKDV 641
7**736847---564768*944545645544--4365366967764484956*97*----7-77---64975974*54675*+363
HmuS 644 KITGQLYTMGVPYEPERITSSVYAMATEPI----AYSLFALDKQRGKAT--ESAEKH------RSVFTQQYLMPARLLVERLMANP 717
ChlH 679 LLPCGLHVIGQPPSAEEAIATLVNIASLDREDEGIWALPTLIAESIGRNMEEIYRNSDKGILADVELLQDITLATRAAVAALVQEQ 764
CobN 642 QIRDGLHILGQNPTGEQELDLVLAILRARQLFGGAHAIPGLRQALGL---AE------DGTDERATVD-QTEAKARELVAA----- 712
5+456*879*68557*73755967975345----788+68*344374----*-----------76474-764738*37*44-----
HmuS 718 --------SLATDELIC-HTAGITPQELAKARQIEAERNAPKGMMAMMMAAAAKKDQADNEPSGNGHPASAKMEKGPHGKMPAGMK 794
ChlH 765 INADGRVSFVSKLNFFKIGKKA----------------PWVK-- 790
CobN 713 LQATGW--------------------DPSAAD------RLTG-- 728
--------------------------------------5565--
HmuS 795 EAMKKMGANMDPEKAMEMAKSMGASPEALKKMEASMKANKDTSTDASGKPAMAGKTEKPQGMSAMMAAMGKAPKEYSKEEVEFALA 880
ChlH 791 ---SLCDSGYPNVN 801
CobN 729 ---NADAAAVLRFA 739
---65444835477
HmuS 881 VAEVERTIKNVGNYKNALLTSPEEELSSLMNALKGGYTAPTPGGDPIA-NPNTLPTGRNMYAINAEATPTESAWEKGIALAKQTID 965
ChlH 802 EEKLKPLFEYLEFCLEQVCAD--NEFGGLLQALEGEYVLPGPGGDPIR-NPNVLPTGKNIHALDPQSIPTLAAVQSAKVVVDRLLE 884
CobN 740 ATEVIPRLAGT-----------ATEIEQVLRALDGRFIPAGPSGSPLRGLVNVLPTGRNFYSVDPKAVPSRLAWEAGVALADSLLA 814
34794548357-----------6*846997**6*397578*8*7*+4-67*8****9*888987687*845*684+5898667+4

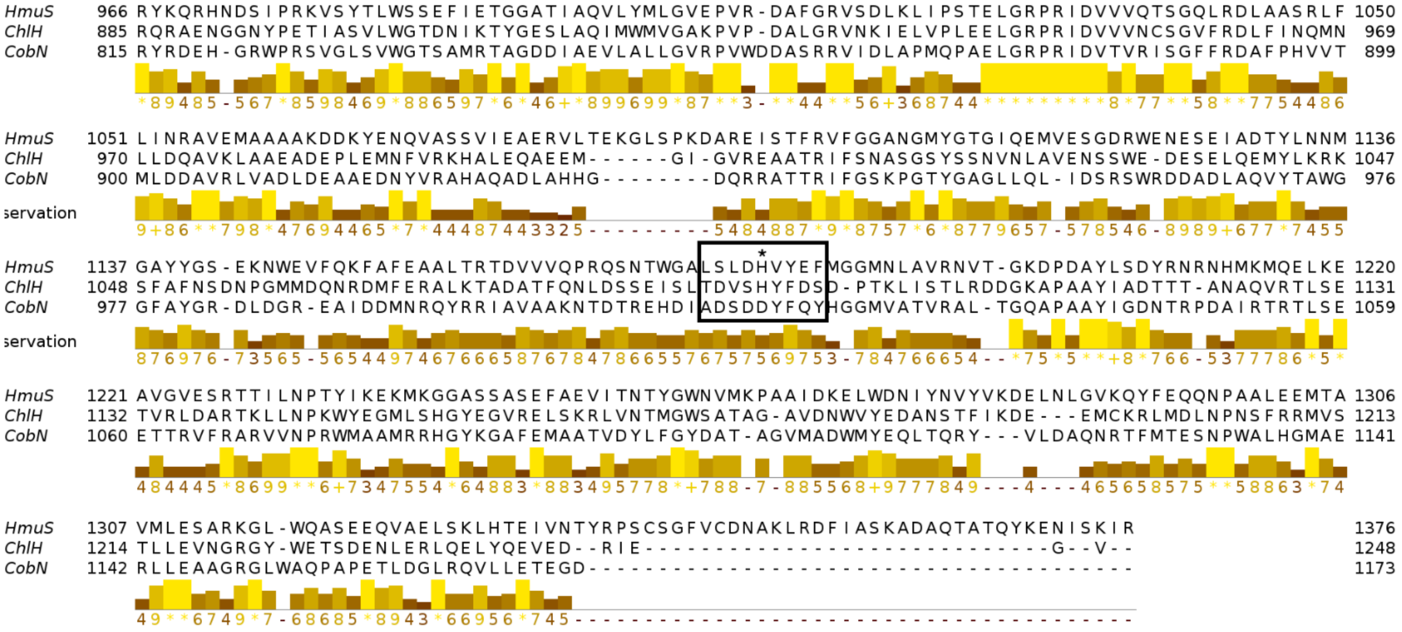

Appendix Figure S12. Structure-based sequence alignment of CobN (6yt0), HmuS (this work) and CobN (7c6o). Structures were aligned using default parameters in FoldMason (https://www.biorxiv.org/content/10.1101/2024.08.01.606130v2). High numbers and gold/yellow conservation block below the alignment indicate residues that are well-conserved in all members. Dark colors or the lack of colored blocks indicate low levels of conservation. Over-represented methionines in the MRI (missing in the structure) are highlighted in green. Black rectangles of arbitrary width are drawn to include the neighborhood around four residues strictly conserved in HmuS proteins: Met-79, His-254, His-538, and His-1209. The residues are marked by black asterisks. Met-79 and His-254 coordinate the heme molecule at the head/neck interface. These residues are not strictly conserved in other type 1 chelatase superfamily members. His-538 (third rectangle) is involved in coordinating the heme molecule in the putative reaction center and is strictly conserved in all chelatase superfamily members. His-1209 (fourth rectangle) is also predicted to coordinate the heme molecule in the putative reaction center, but it is not universally conserved.

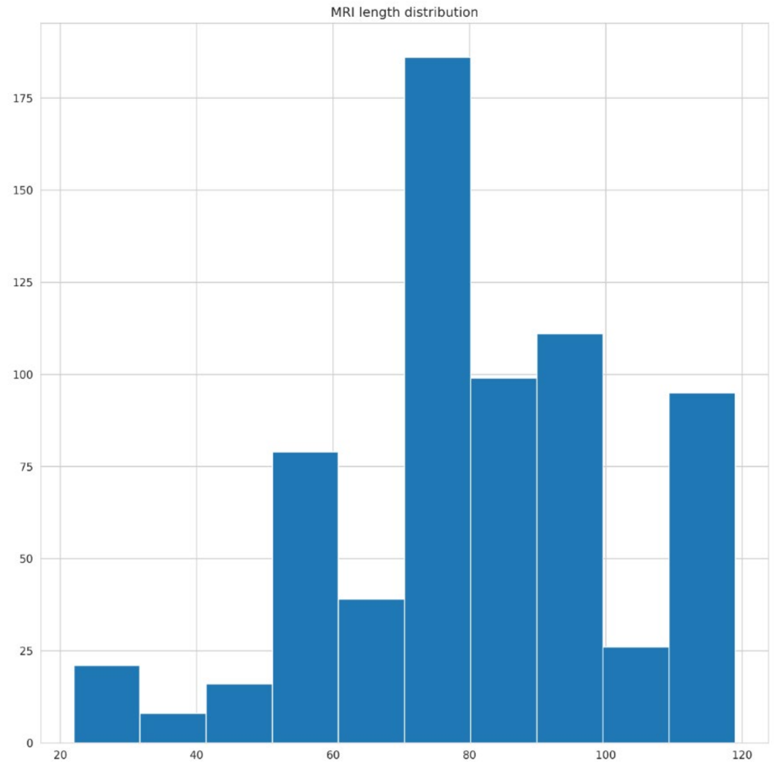

Appendix Figure S13. Length distribution of MRIs from the operonic set of HmuS sequences. A subset of HmuS sequences corresponding to *B. theta* HmuS residues 780-898 was excised from the multiple sequence alignment. Fragment lengths were determined for all sequences and their distribution is shown as a histogram.

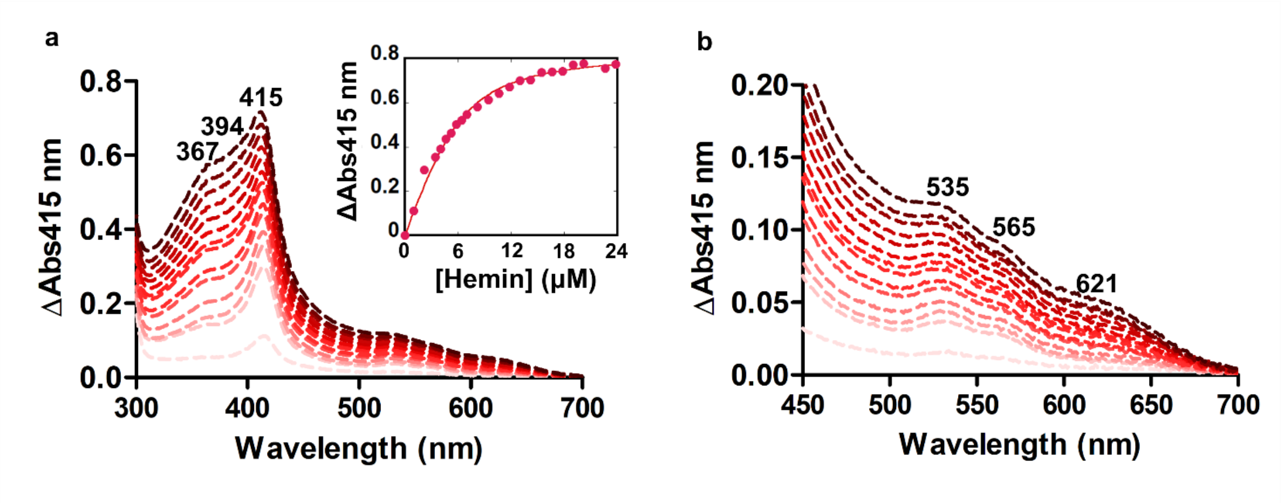

Appendix Figure S14. UV/visible absorption spectroscopy illustrating heme titration to the HmuS H538A mutant. HmuS (H538A) (6 μM, containing 0.35 μM HmuS-heme complex) was titrated in air with a basic hemin solution (pH 8, 0.6 μM aliquots). Changes to the UV/vis absorbance were monitored up to 24 μM of hemin added. A cuvette containing buffer alone was titrated in parallel and used as a blank. Difference spectra are shown. Inset: plot of absorbance at 415 nm versus [hemin], fit to a quadratic binding equation. (b) Q-band region from (a) is shown on an expanded scale.

APPENDIX TABLES

Appendix Table S1. Substrate and product analyses for heme-PPIX conversion reactions† using *B theta* cellular fractions. These data are plotted as a bar chart in Figure 2.

Sample #	Fraction assayed	Amount of fraction assayed	Assay additions	Unreacted [heme] post-incubation (μM)	[PPIX] product post-reaction (μM)	% turn-over: [PPIX] ([heme] + [PPIX])$^{-1}$ x 100%
1	*B theta* whole cell lysate	300 μL	None	30 ± 1.6	5.2 ± 1	15
2	*B theta* whole cell lysate, dialyzed	300 μL	None	32 ± 0.7	4.6 ± 0.4	13
3	*B theta* whole cell lysate, dialyzed + NADH	300 μL	1 mM NADH	12 ± 0.3	29 ± 0.9	71
4	*B theta* whole cell lysate, dialyzed + ATP	300 μL	1 mM ATP	30 ± 0.8	0	0
5	*B theta* whole cell lysate, dialyzed + NADH + ATP	300 μL	1 mM NADH, 1 mM ATP	13 ± 0.7	26 ± 0.3	67
6	*B theta* membranes	300 μL	1 mM NADH	8.3 ± 0.5	36 ± 0.3	81
7	*B theta* soluble lysate fraction	300 μL	1 mM NADH	23 ± 2	13 ± 2	36

† 1g *B. theta* cell pellet was resuspended in 15 mL of 20 mM Tris-HCl (pH 7.1). Dialysis against the same buffer was carried out using 10 kDa MWCO tubing (3 buffer exchanges after >3h each, 4 degrees C). Dialyzed cell lysate (4 mL) was pelleted and the supernatant used as the soluble cell fraction. The pellet was washed and resuspended in 4 mL reaction buffer and used as the membrane fraction.Standard reaction conditions: 100 μM hemin, 40 min room temperature, 330 μL final reaction volume, 20 mM Tris-HCl buffer, pH 7. Extraction conditions: 300 μL or 600 μL ACN + 12M HCl + DMSO (41:9:50). Extraction efficiencies (Figure S1) are near 50% for both heme and PPIX in this solvent. Analyte concentrations in the extracts were measured via HPLC peak integration and comparison with standard curves and corrected to reflect the concentration in the initial reaction volume. ≥3 reactions were analyzed for substrate-product turnover and analyte concentrations averaged.

Appendix Table S2. Typical outcomes for recombinant HmuS expression and purification†

Fraction	Volume (mL)	Protein concentration (mg mL^{-1})	Total protein (mg)	Yield after step (% by mass)	Total yield (% by mass)
HmuS-containing clarified lysate	100	23	2300	100	100
Anion exchange	40	15	600	26	26
Size exclusion chromatography	20	5	100	16	4.4
Centrifuge concentration (50 MWCO)	9	10	90	15	3.9

† For HmuS purification, 14 g cell pellets were generated per L of culture, frozen, and resuspended in 100 mL of 20 mM Tris-HCl, 250 mM NaCl, pH 7.1 buffer, lysed by sonication, and ultracentrifuged as described in the text. Protein concentrations were measured by Bradford analysis (BSA, 0.1 mg ml^{-1}, Bradford dye as the working dye, standard curve was constructed with triplicate data points at 595 nm).

Appendix Table S3. Cryo-EM data collection, processing, model refinement and validation.

**Data collection**		
Microscope	Talos Arctica	
Voltage (kV)	200	
Detector	K3 (Counting)	
Magnification (nominal/calibrated)	×45,000/×55,187	
Exposure navigation	Image shift to 25 holes	
Electron exposure (e^-/Å^2)	56 e^- Å^2	
Data acquisition software	SmartScope/SerialEM	
Total electron exposure (e^-/Å^2)	56	
Exposure rate (e-/pixel/sec)	18.3	
Frame length (ms)	60	
Number of frames per micrograph	51	
Pixel size (Å)	0.9061	
Defocus range (µm)	-0.6 to -1.5	
Micrographs collected (0° tilt)	7,800	
Micrographs collected (15° tilt)	8,194	
**Reconstruction**	**EMD-46483 PDB 9D26**	**EMD-71280 PDB 9P4S**
Image processing package	cryoSPARC	cryoSPARC
Total extracted particles (blob picks)	18,955,798	18,955,798
Final number particles	2,133,842	3,795,134
Symmetry imposed	C1	C1
Resolution (Å)		
FSC 0.143 (masked/unmasked)	2.60/2.9	2.55/2.9
**Model Composition (#)**		
Chains	1	1
Atoms	9855	8808
Residues	Protein: 1254	Protein: 1120
Water	95	95
Ligands	HEM: 1<br>NA: 2	NA: 2
**Model Refinement**		
Refinement Package	Phenix	Phenix
Bonds (RMSD)		
Length (Å) (# > 4σ)	0.003 (0)	0.002 (0)
Angles (°) (# > 4σ)	0.442 (0)	0.448 (0)
**Model Validation**		
MolProbity score	0.82	0.95
Clash score	1.12	1.43
Ramachandran (%)		
Outliers	0	0
Allowed	1.76	2.33
Favored	98.24	97.67
Rama-Z		
whole (N = 1254)	-1.06 (0.22)	1.07 (0.25)
helix (N = 650)	-0.5 (0.18)	2.15 (0.22)
sheet (N = 139)	1.42 (0.47)	0.67 (0.48)
loop (N = 576)	-1.51 (0.25)	-1.67 (0.26)
Outliers		
Rotamer outliers (%)	0.86	0.21
Cβ outliers (%)	0.00	0.00
Peptide Plane, Cis Pro/general (%)	5.6/0.2	6.1/0.2
Peptide Plane, Twisted Pro/general (%)	0.0/0.0	0.0/0.0

CaBLAM outliers (%)	1.44	1.53
ADP (B-factors)		
Iso/Aniso (#)	9995/0	8905/0
min/max/mean		
Protein	76.2/234.8/127.8	69.9/232.9/119.5
Ligand	108.6/177.1/127.8	101.6/111.6/106.6
Water	86.4/140.3/116.9	82.0/136.0/110.3
Occupancy		
Mean	1.0	1.0
occ = 1 (%)	100.0	100.0
0 < occ < 1 (%)	0.0	0.0
occ > 1 (%)	0.0	0.0
**Model vs. Data**		
Resolution (Å)	Masked Unmasked	Masked Unmasked
d FSC model (0.5)	2.7 2.8	2.7 2.8
CC (mask)	0.86	0.85
CC (box)	0.84	0.84
CC (peaks)	0.79	0.78
CC (volume)	0.86	0.85
Mean CC for ligands	0.47	0.47
