# Supplementary material for: Commensal gut bacteria employ de-chelatase HmuS to harvest iron from heme
Source: EMBO J. 2025 Sep 12;44(21):6226–52. doi: 10.1038/s44318-025-00563-5 (PMC12583661; doi:10.1038/s44318-025-00563-5)
Supplement: Supplementary file 7 — Source data Fig. 1 [file 44318_2025_563_MOESM7_ESM.zip › Fig. 1/README_Fig1.docx]

This figure is a schematic created using BioRender. The arrow diagram in (a) representing the hmu operon was drawn to scale from genomic data curated by the National Center for Bioinformatics (NCBI).
