# Supplementary figures and images for: Commensal gut bacteria employ de-chelatase HmuS to harvest iron from heme
Source: EMBO J. 2025 Sep 12;44(21):6226–52. doi: 10.1038/s44318-025-00563-5 (PMC12583661; doi:10.1038/s44318-025-00563-5)

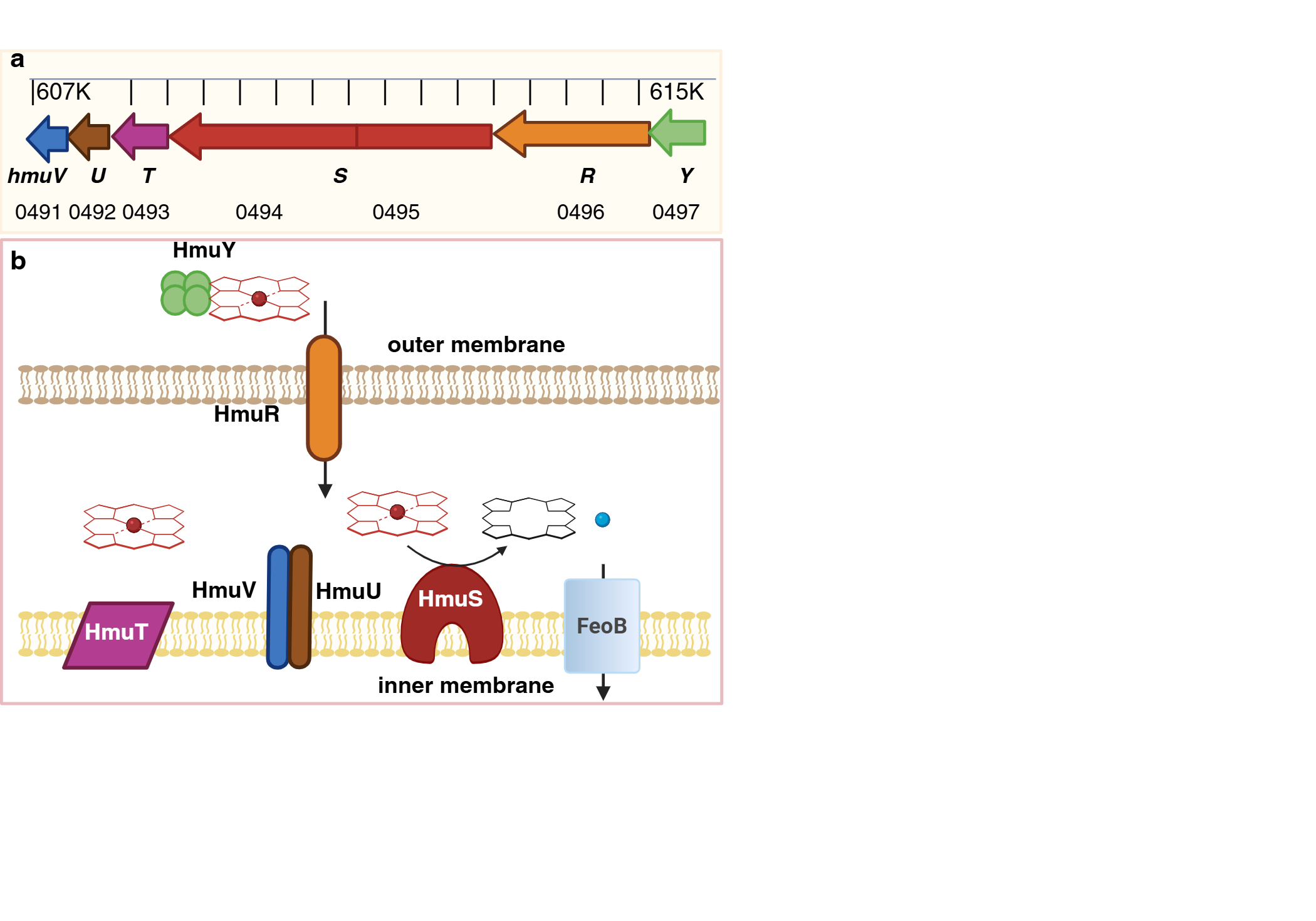

Supplement: Supplementary file 7 — Source data Fig. 1 [file 44318_2025_563_MOESM7_ESM.zip › Fig. 1/Figure 1 Hmu Operon.jpeg]

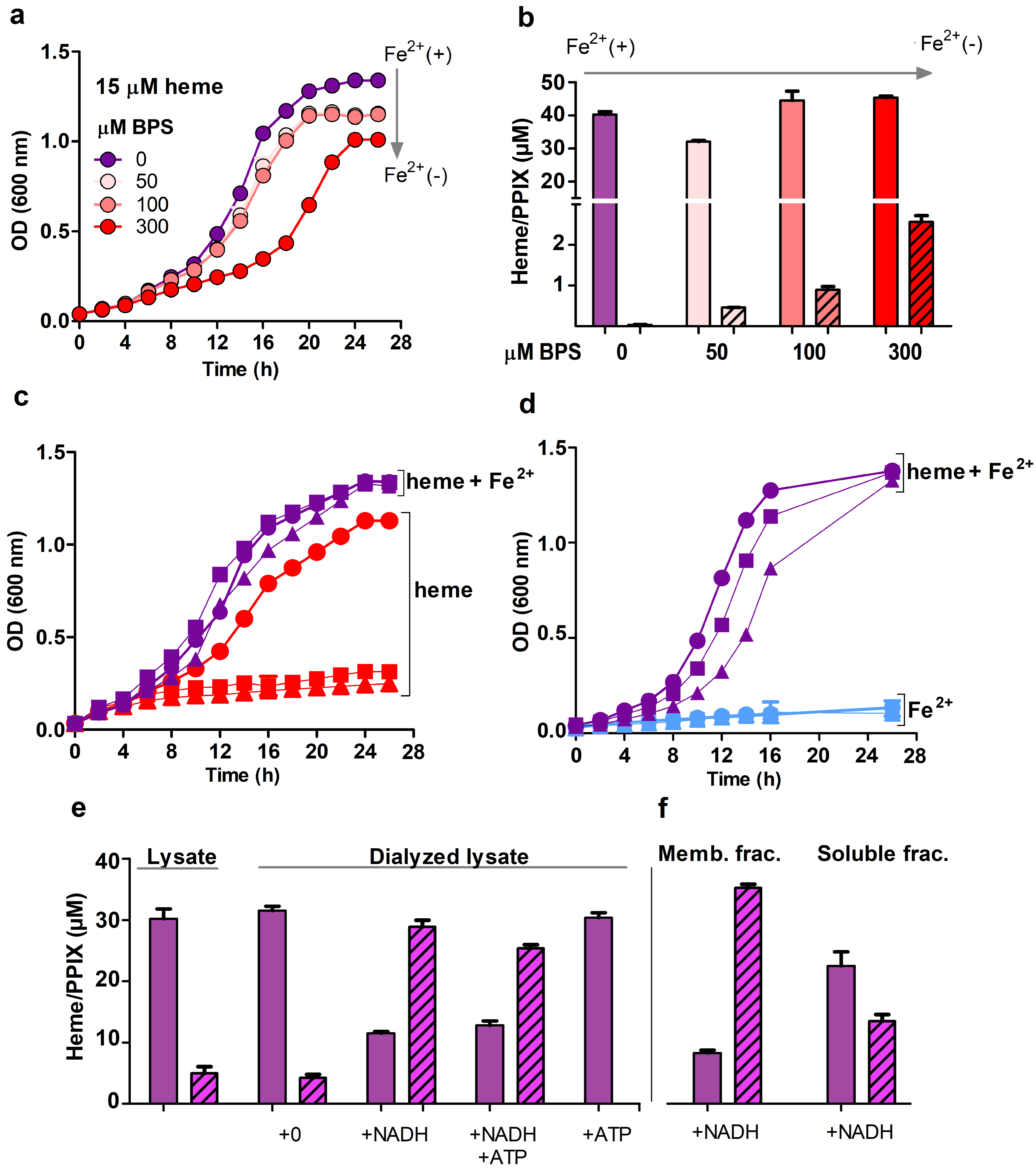

Supplement: Supplementary file 8 — Source data Fig. 2 [file 44318_2025_563_MOESM8_ESM.zip › Fig. 2/Fig 2.tif]

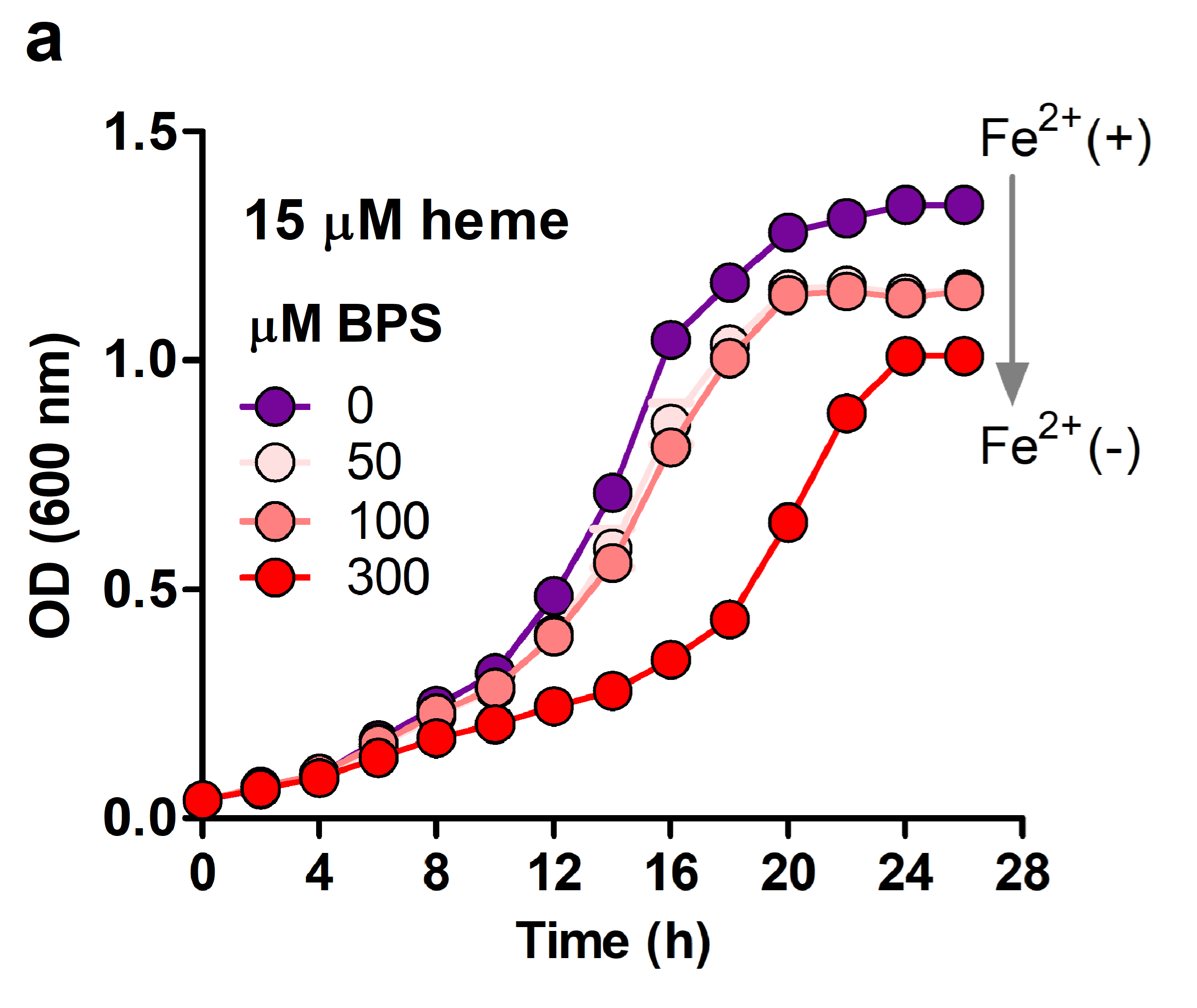

Supplement: Supplementary file 8 — Source data Fig. 2 [file 44318_2025_563_MOESM8_ESM.zip › Fig. 2/Fig 2a/Fig 2a.tif]

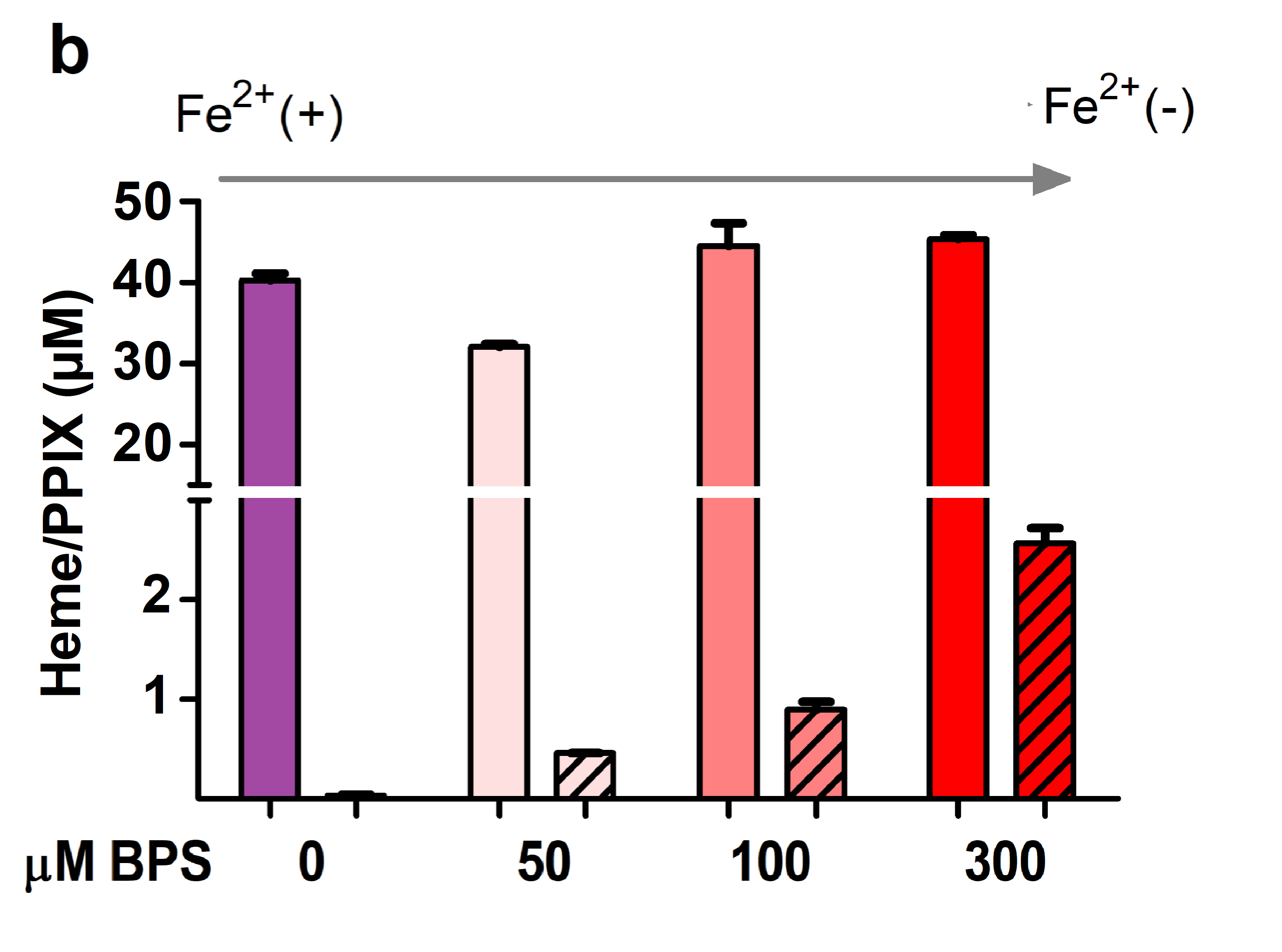

Supplement: Supplementary file 8 — Source data Fig. 2 [file 44318_2025_563_MOESM8_ESM.zip › Fig. 2/Fig 2b/Fig 2b.tif]

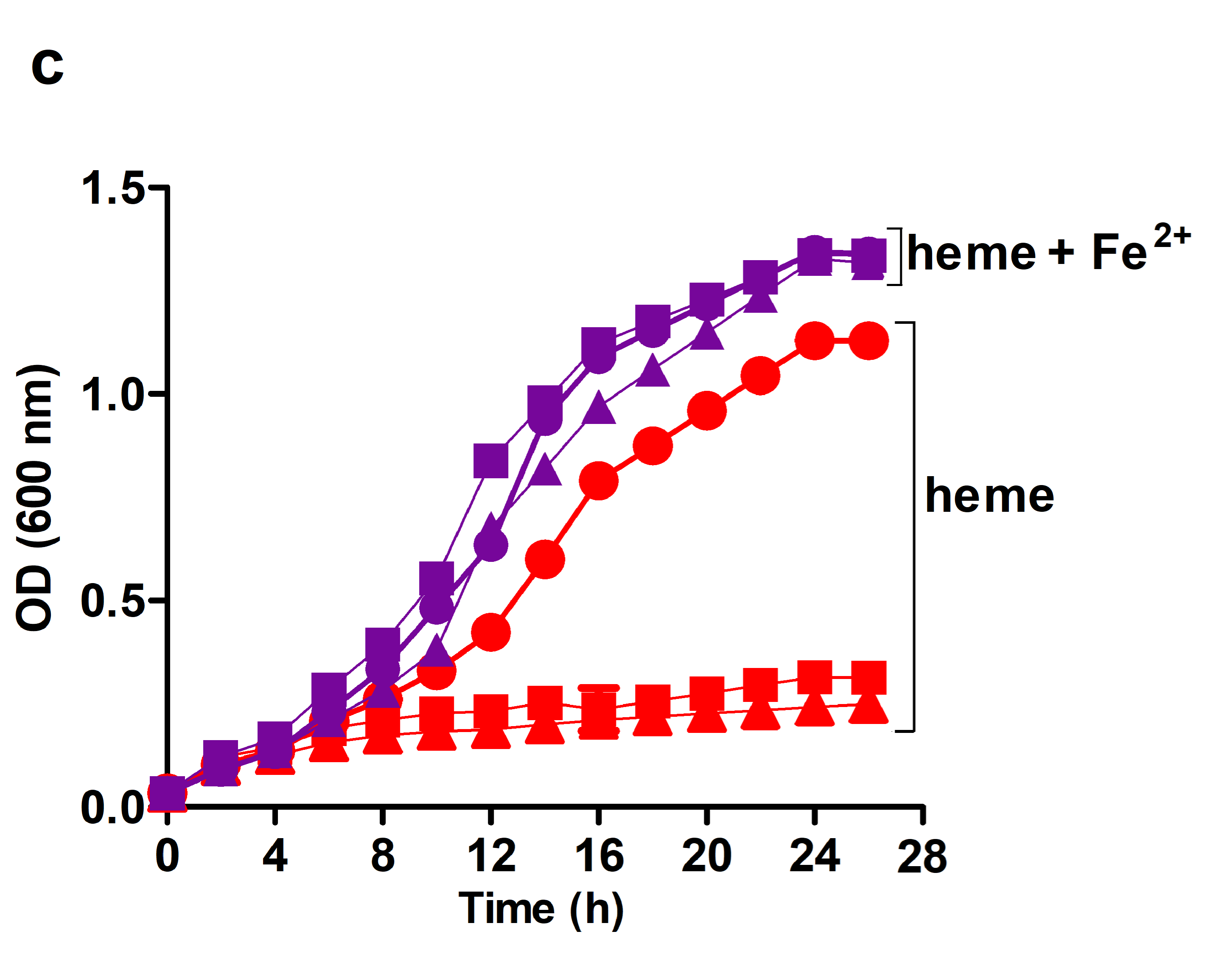

Supplement: Supplementary file 8 — Source data Fig. 2 [file 44318_2025_563_MOESM8_ESM.zip › Fig. 2/Fig 2c/Fig 2c.tif]

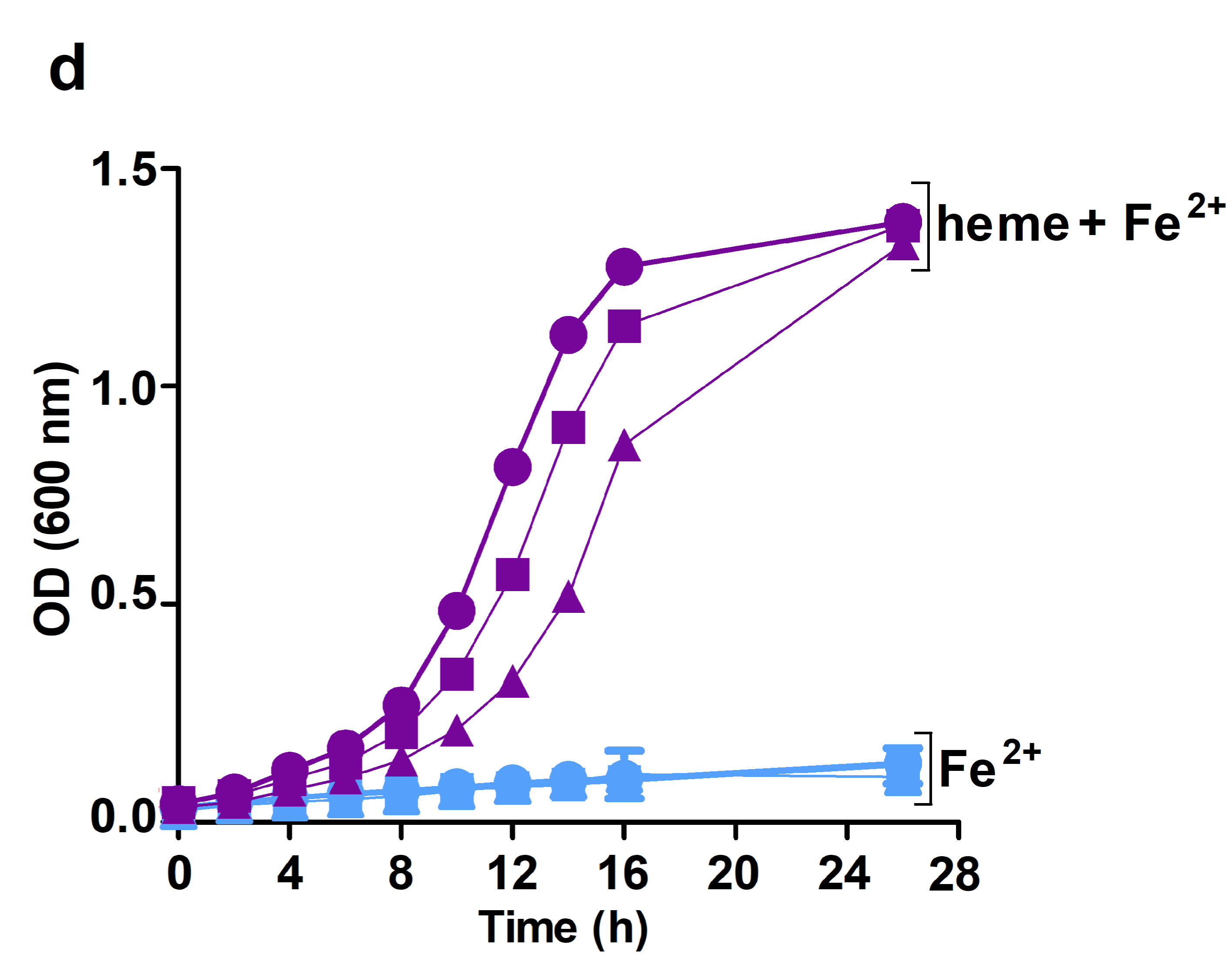

Supplement: Supplementary file 8 — Source data Fig. 2 [file 44318_2025_563_MOESM8_ESM.zip › Fig. 2/Fig 2d/Fig 2d.tif]

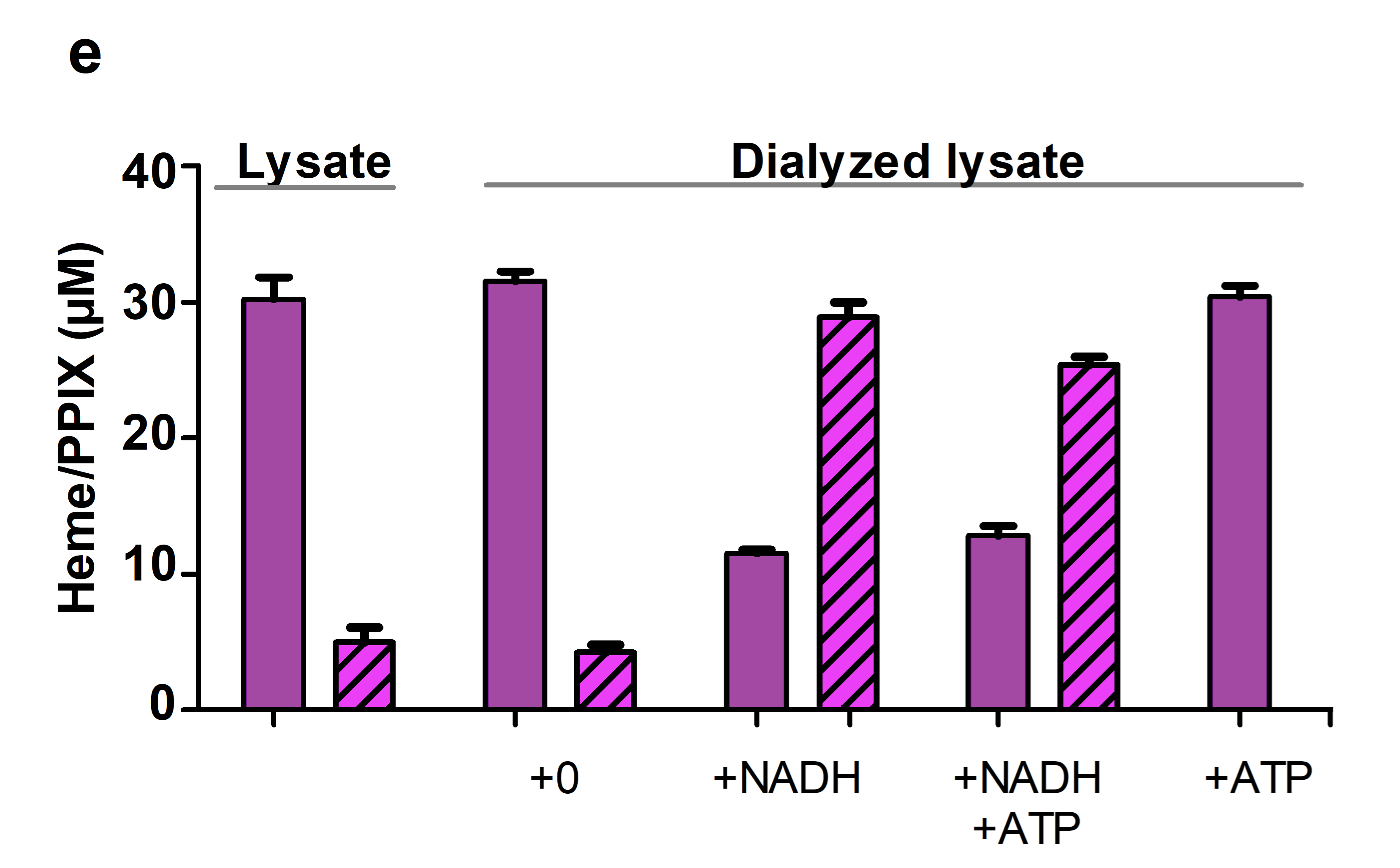

Supplement: Supplementary file 8 — Source data Fig. 2 [file 44318_2025_563_MOESM8_ESM.zip › Fig. 2/Fig 2e/Fig 2e.tif]

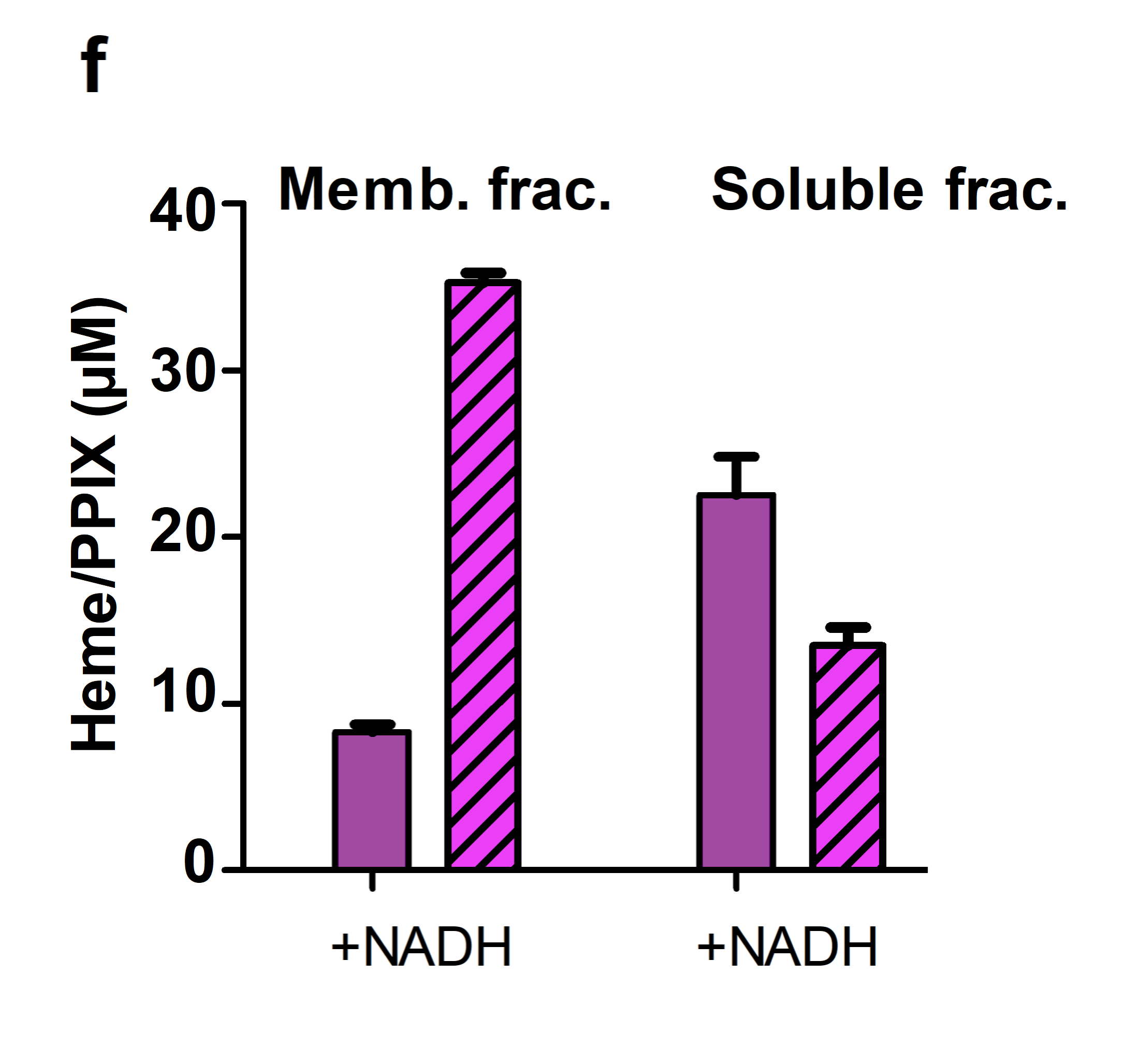

Supplement: Supplementary file 8 — Source data Fig. 2 [file 44318_2025_563_MOESM8_ESM.zip › Fig. 2/Fig 2f/Fig 2f.tif]

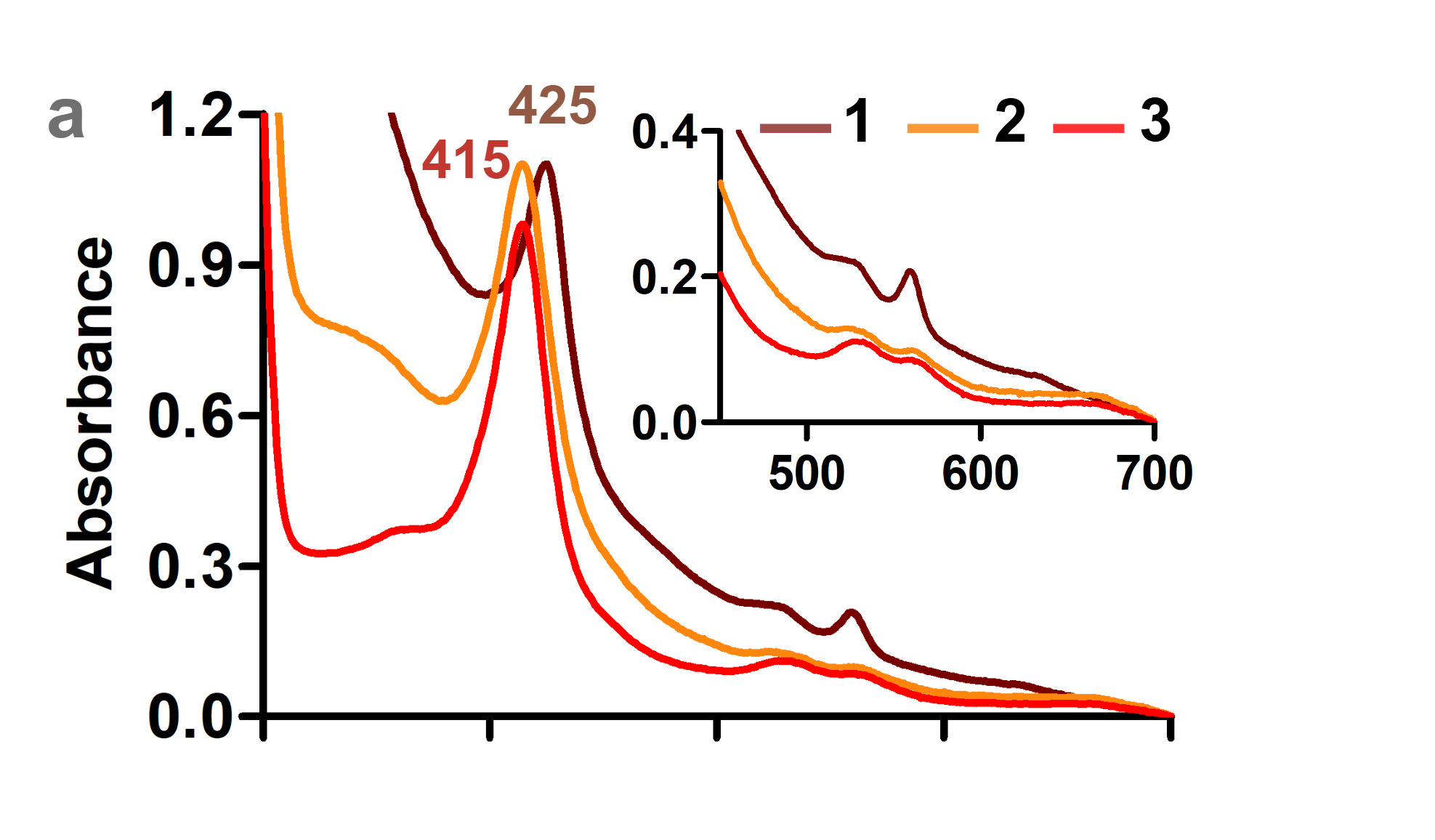

Supplement: Supplementary file 9 — Source data Fig. 3 [file 44318_2025_563_MOESM9_ESM.zip › Fig. 3/Fig 3a/Figure3a_EMBO.png]

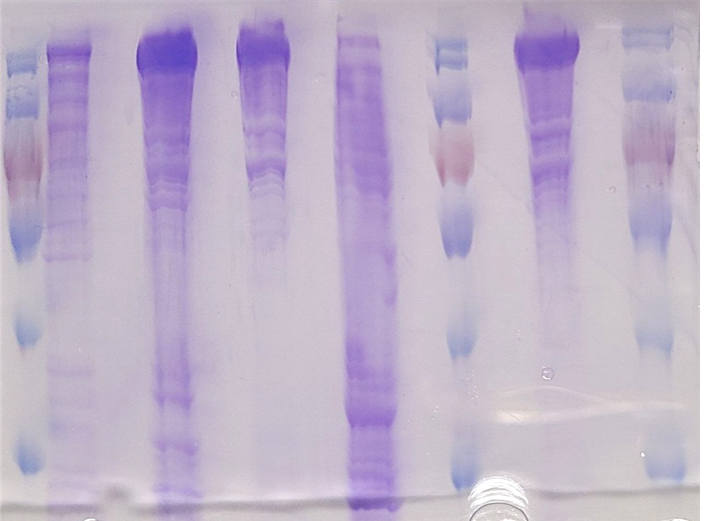

Supplement: Supplementary file 9 — Source data Fig. 3 [file 44318_2025_563_MOESM9_ESM.zip › Fig. 3/Fig 3b-Gel/Fig 3B source image.png]

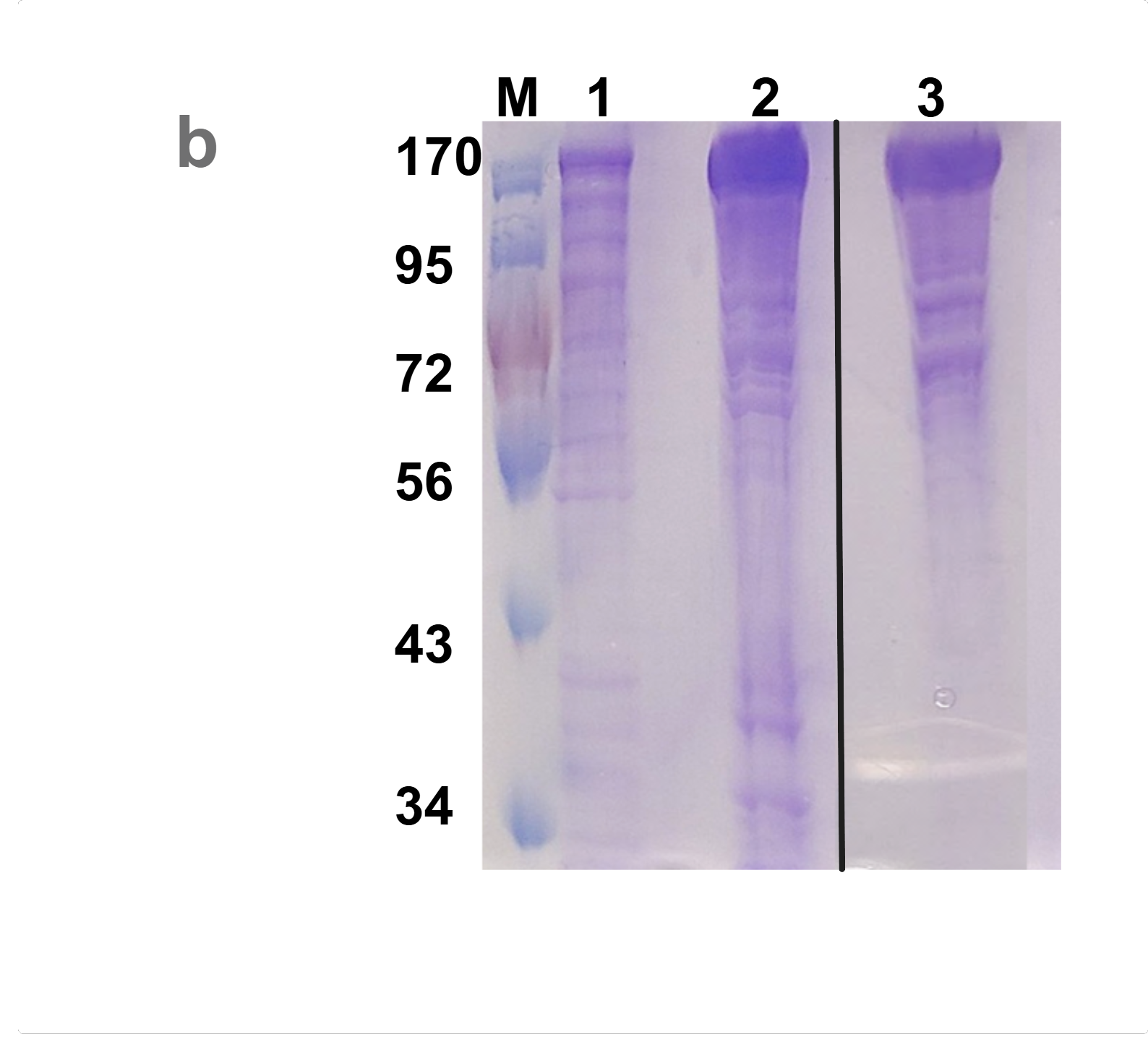

Supplement: Supplementary file 9 — Source data Fig. 3 [file 44318_2025_563_MOESM9_ESM.zip › Fig. 3/Fig 3b-Gel/Figure3b_EMBO.png]

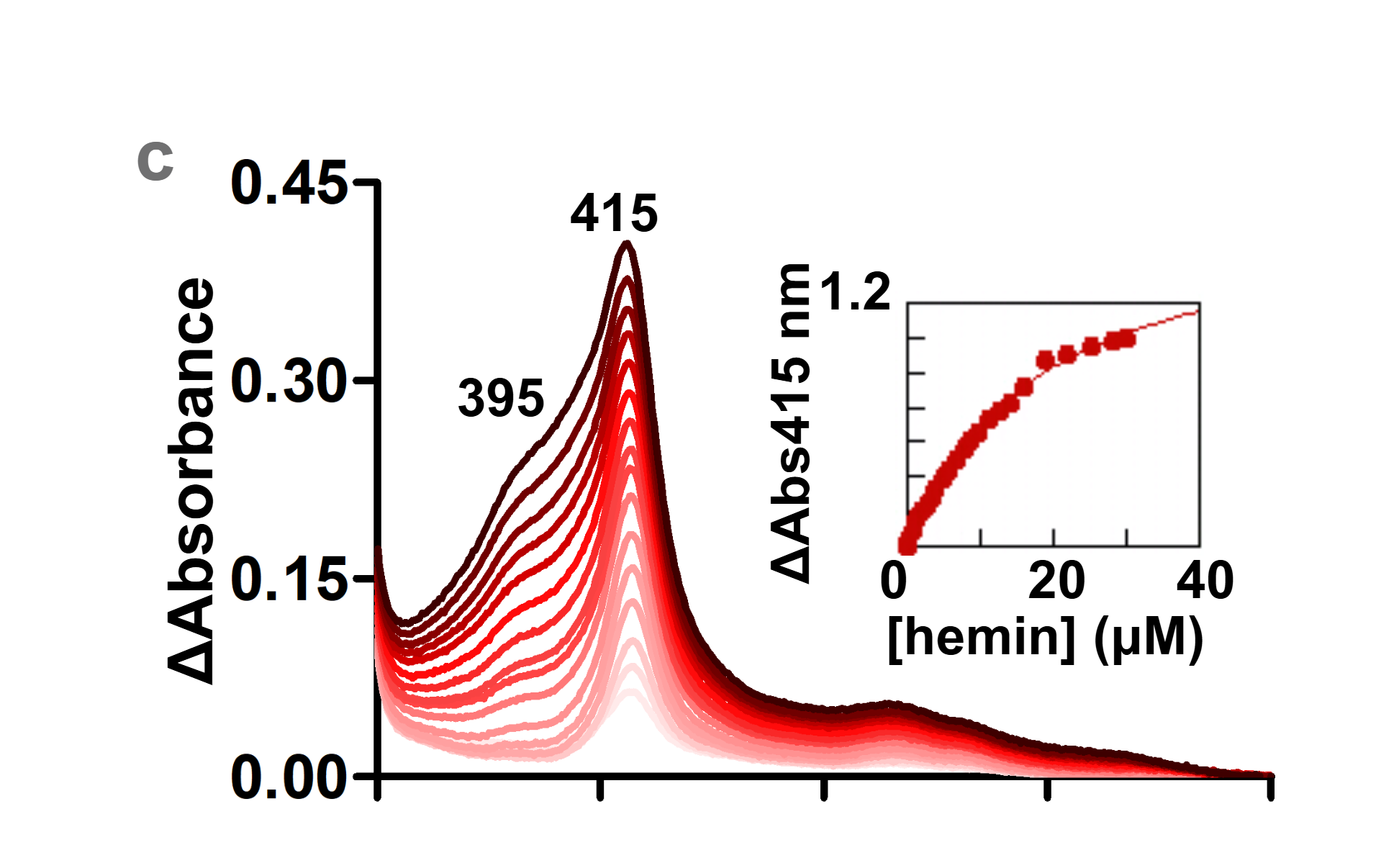

Supplement: Supplementary file 9 — Source data Fig. 3 [file 44318_2025_563_MOESM9_ESM.zip › Fig. 3/Fig 3c/Figure3c_EMBO.png]

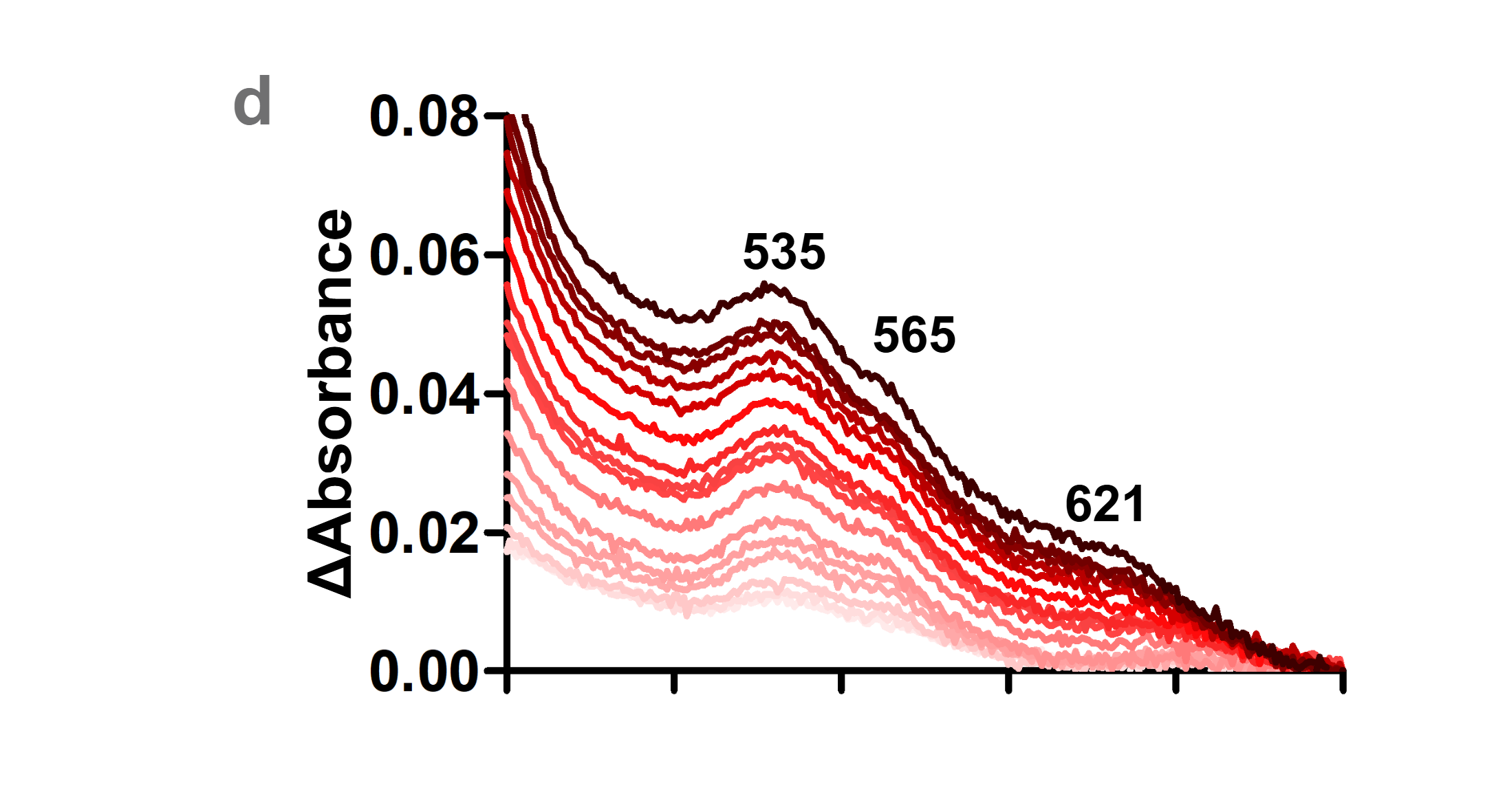

Supplement: Supplementary file 9 — Source data Fig. 3 [file 44318_2025_563_MOESM9_ESM.zip › Fig. 3/Fig 3d/Figure3d_EMBO.png]

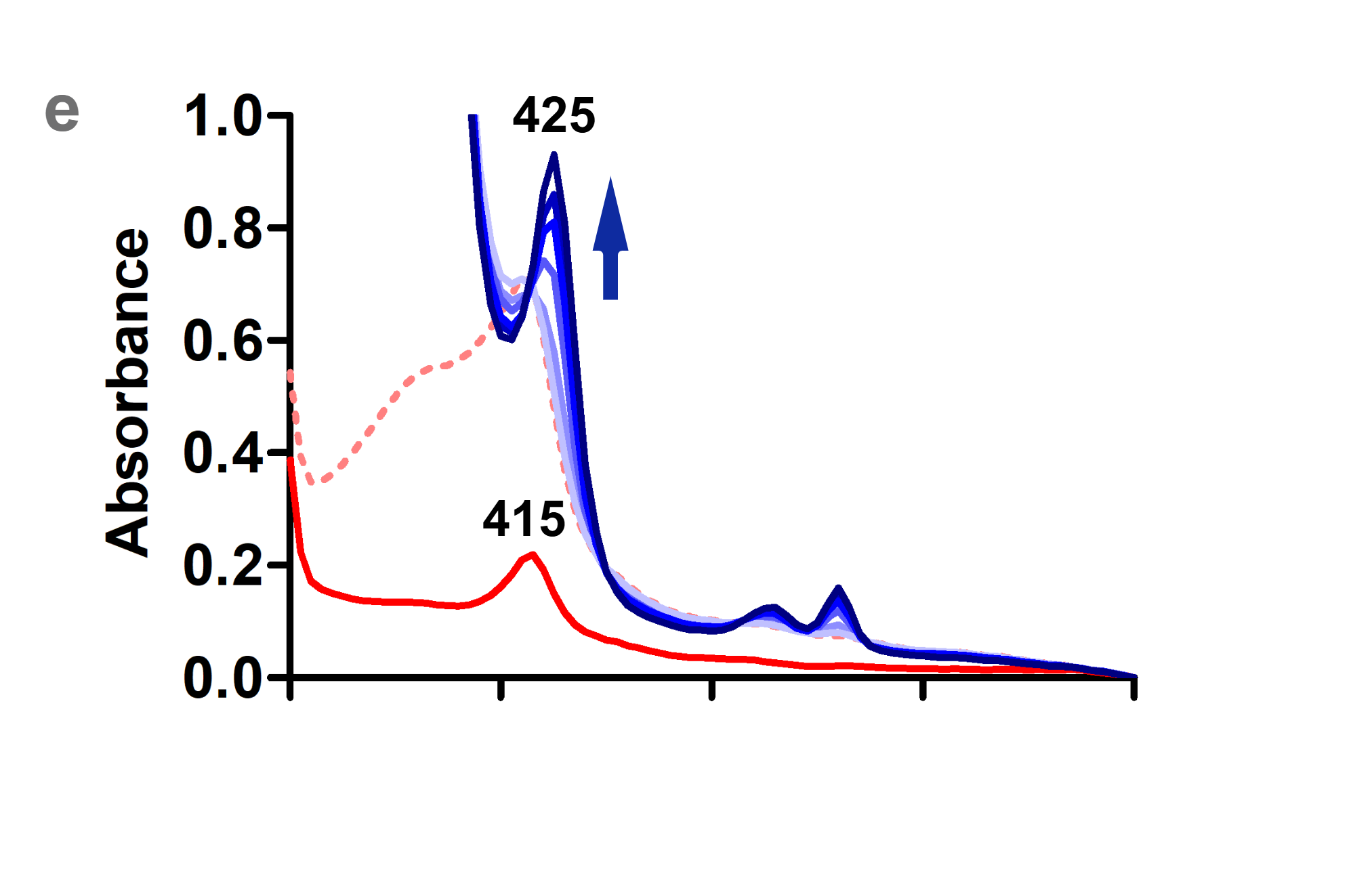

Supplement: Supplementary file 9 — Source data Fig. 3 [file 44318_2025_563_MOESM9_ESM.zip › Fig. 3/Fig 3e/Figure3e_EMBO.png]

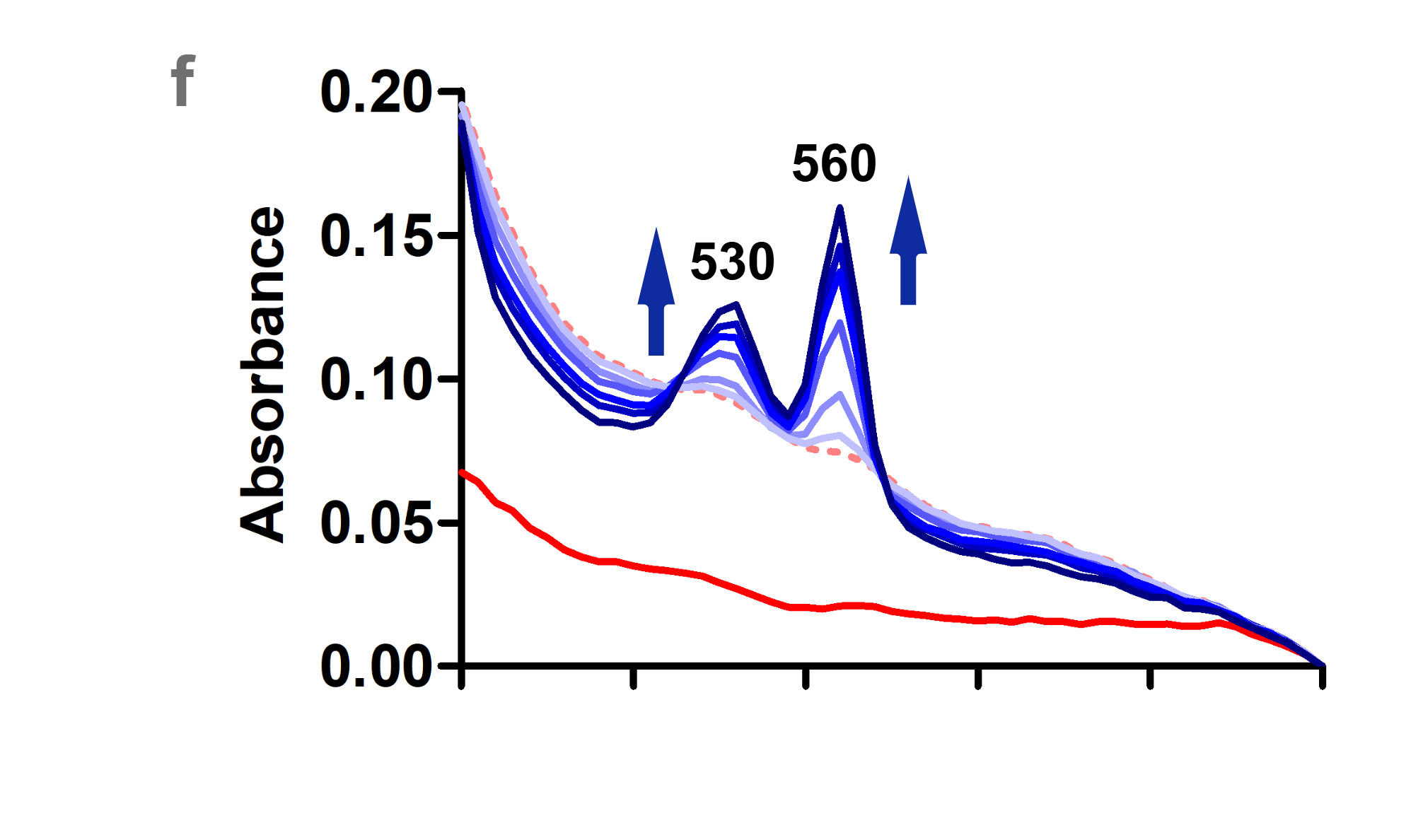

Supplement: Supplementary file 9 — Source data Fig. 3 [file 44318_2025_563_MOESM9_ESM.zip › Fig. 3/Fig 3f/Figure3f_EMBO.png]

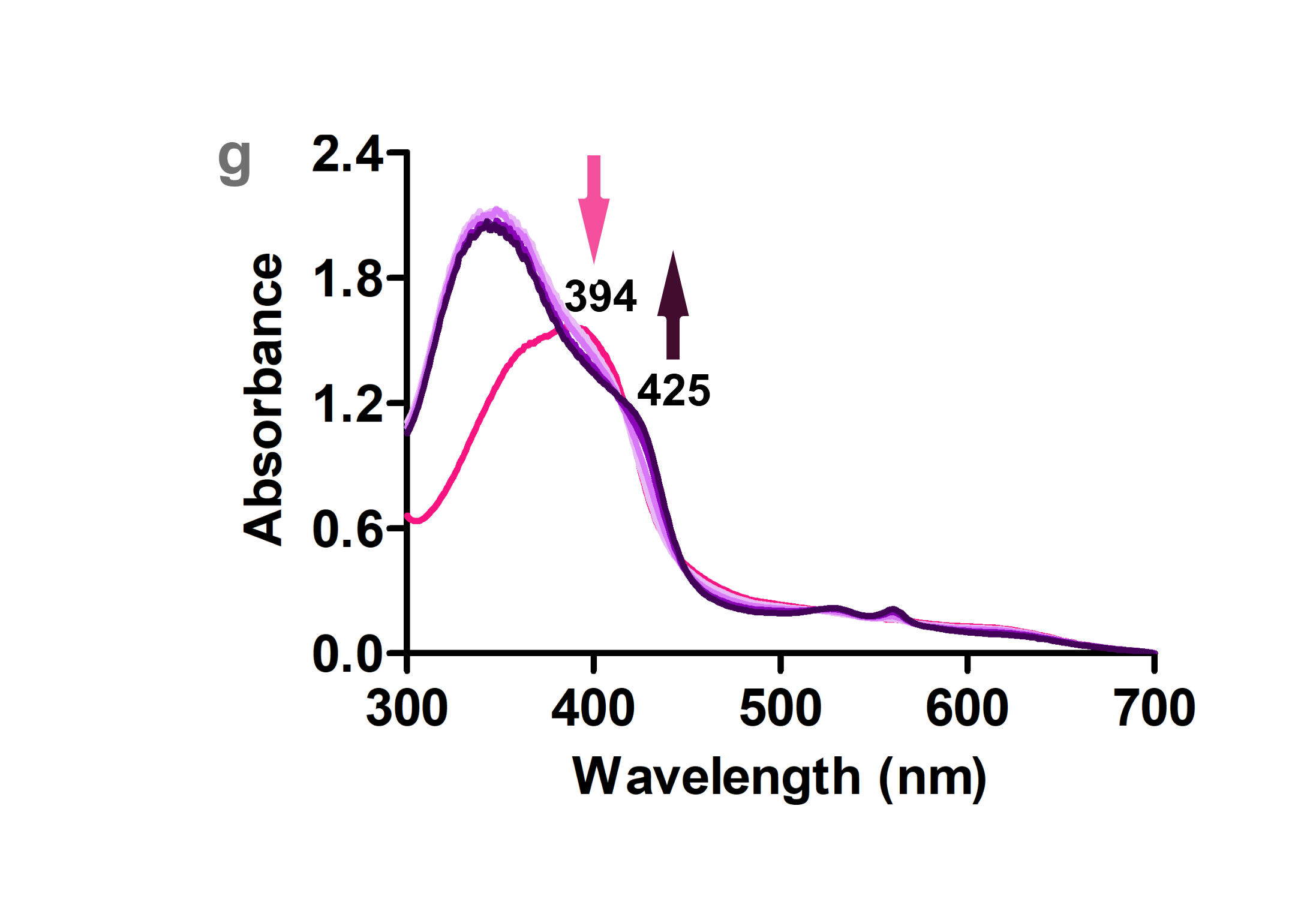

Supplement: Supplementary file 9 — Source data Fig. 3 [file 44318_2025_563_MOESM9_ESM.zip › Fig. 3/Fig 3g/Figure3g_EMBO.png]

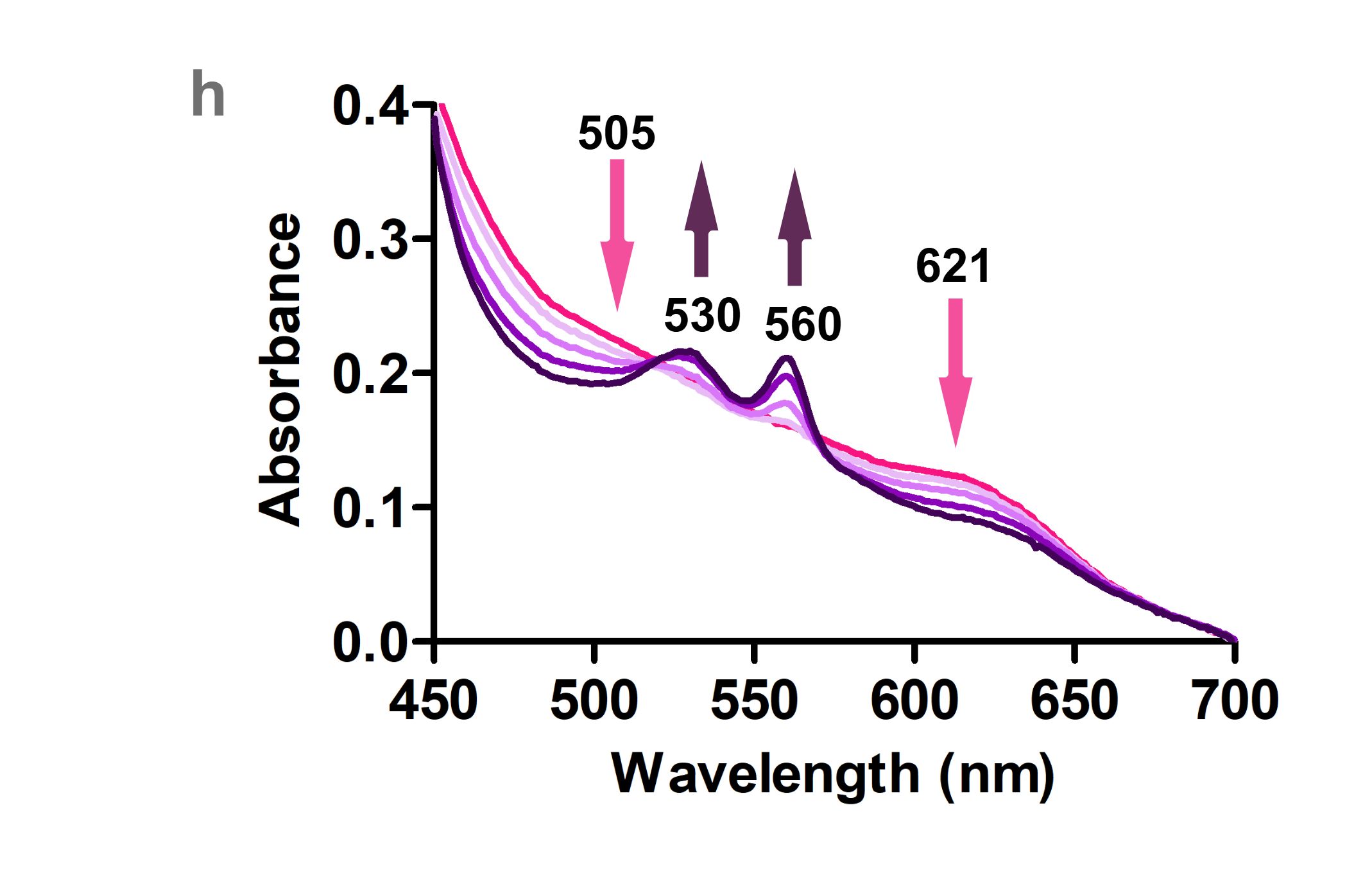

Supplement: Supplementary file 9 — Source data Fig. 3 [file 44318_2025_563_MOESM9_ESM.zip › Fig. 3/Fig 3h/Figure3h_EMBO.png]

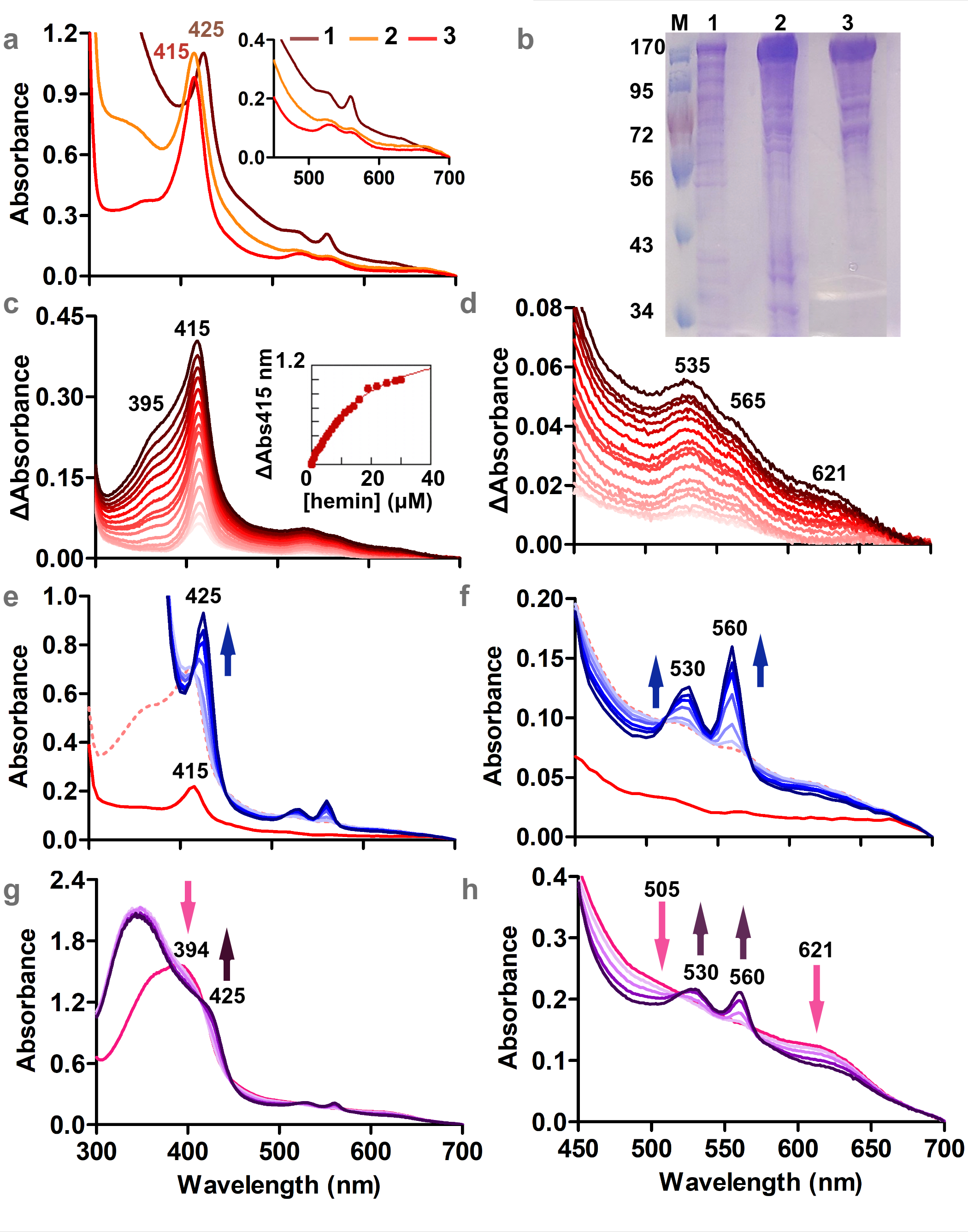

Supplement: Supplementary file 9 — Source data Fig. 3 [file 44318_2025_563_MOESM9_ESM.zip › Fig. 3/Fig. 3.jpeg]

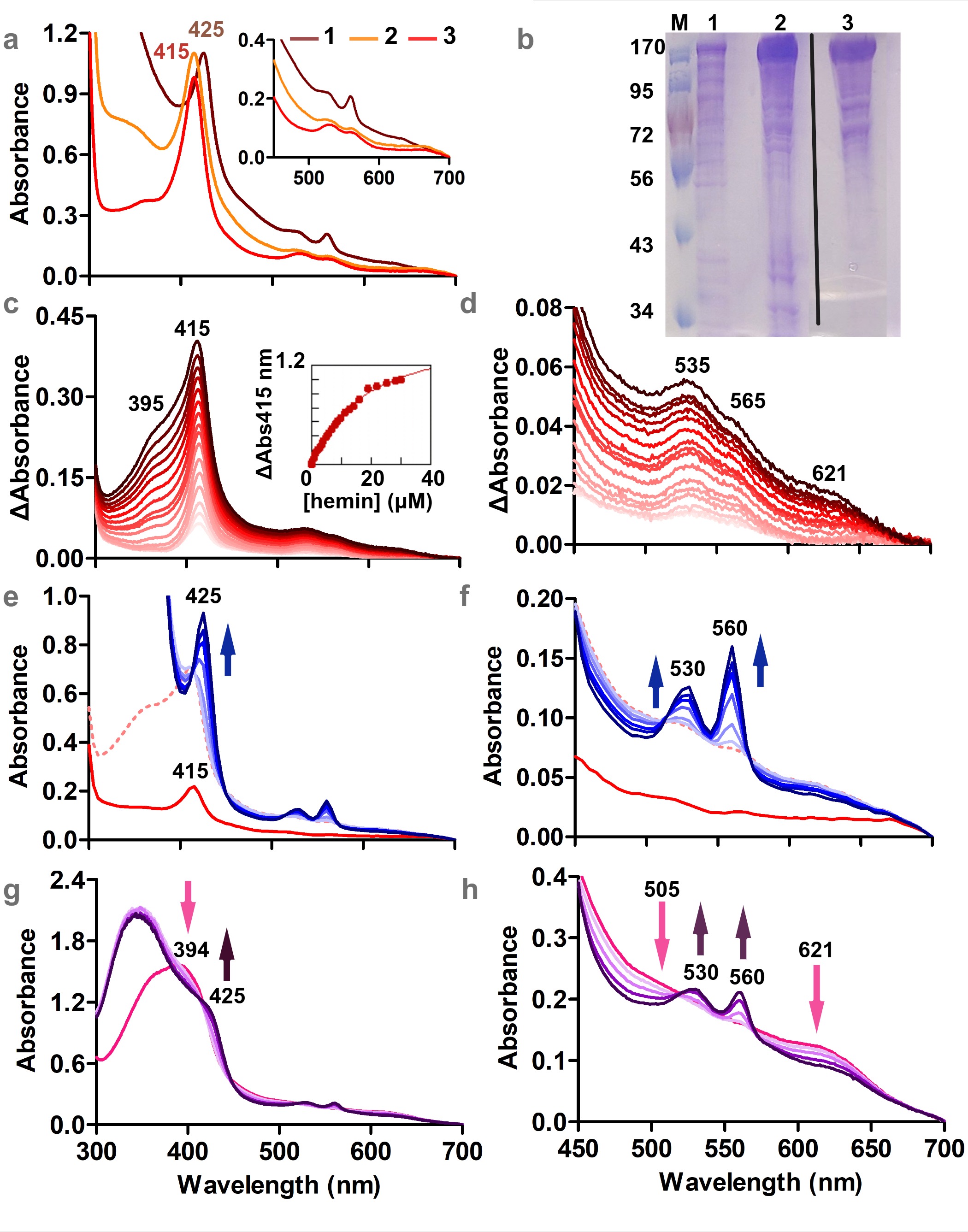

Supplement: Supplementary file 9 — Source data Fig. 3 [file 44318_2025_563_MOESM9_ESM.zip › Fig. 3/Fig. 3.JPG]

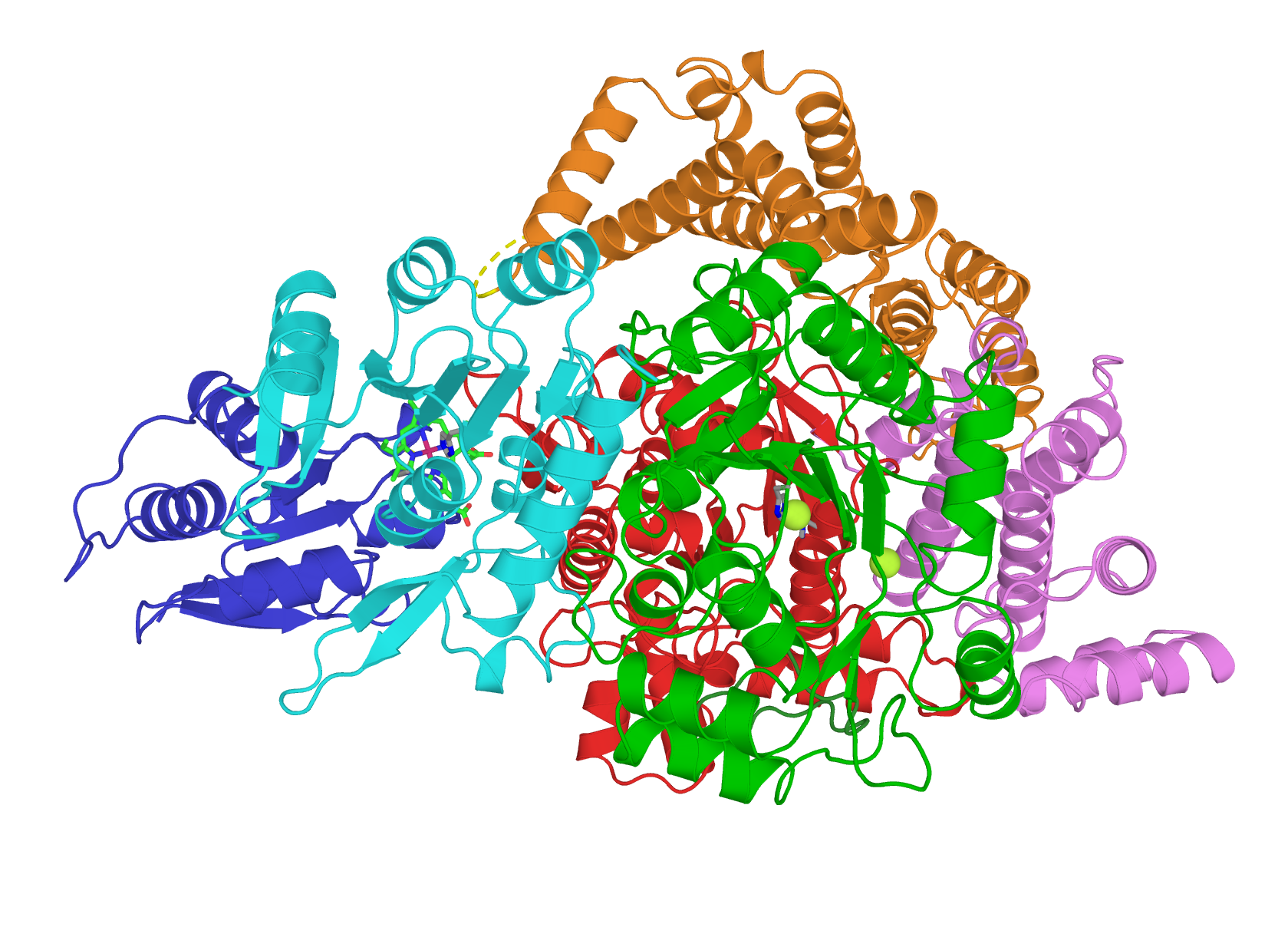

Supplement: Supplementary file 10 — Source data Fig. 4 [file 44318_2025_563_MOESM10_ESM.zip › Fig. 4/Fig 4b/Panel 4B.png]

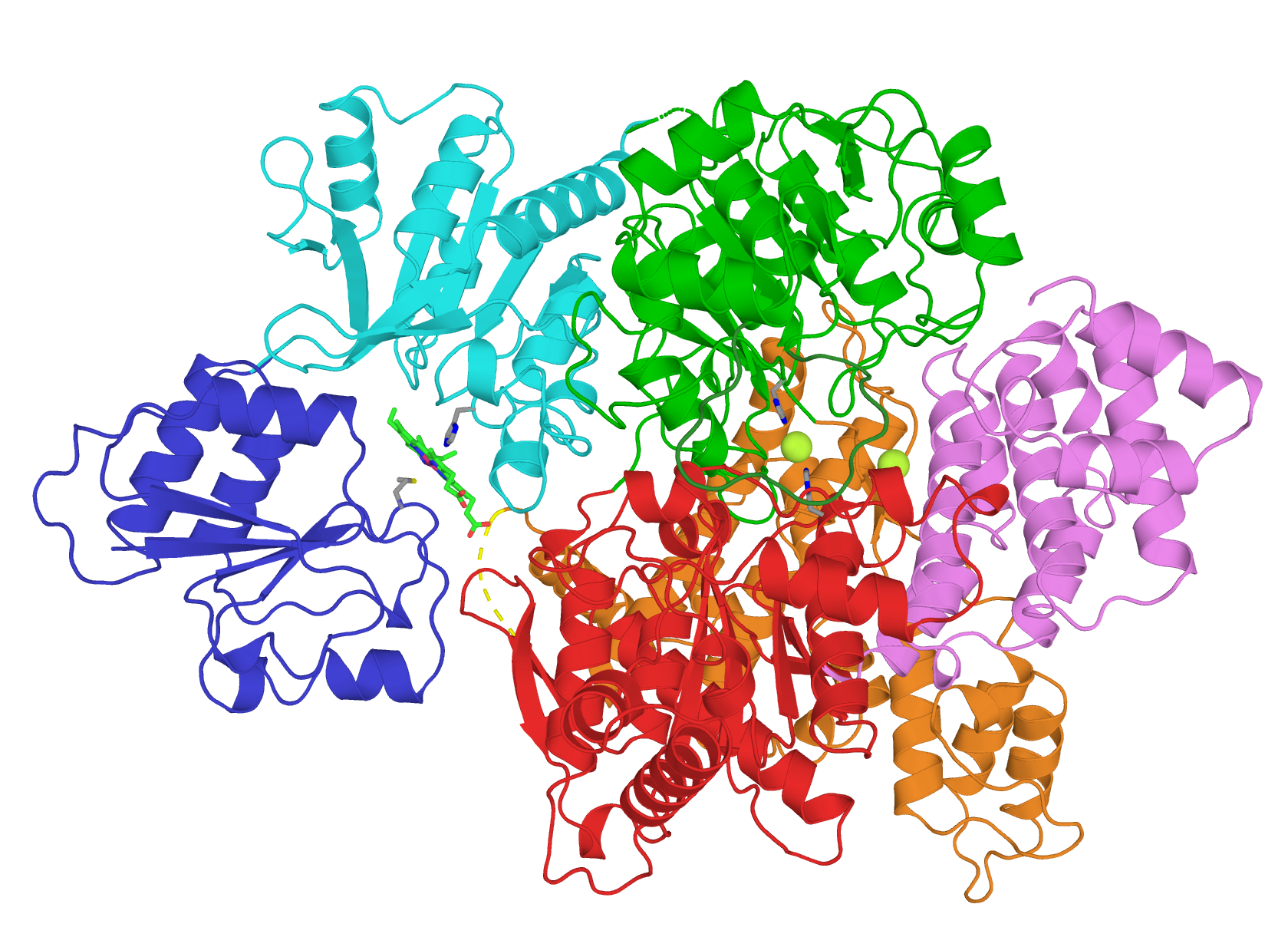

Supplement: Supplementary file 10 — Source data Fig. 4 [file 44318_2025_563_MOESM10_ESM.zip › Fig. 4/Fig 4c/Panel 4C.png]

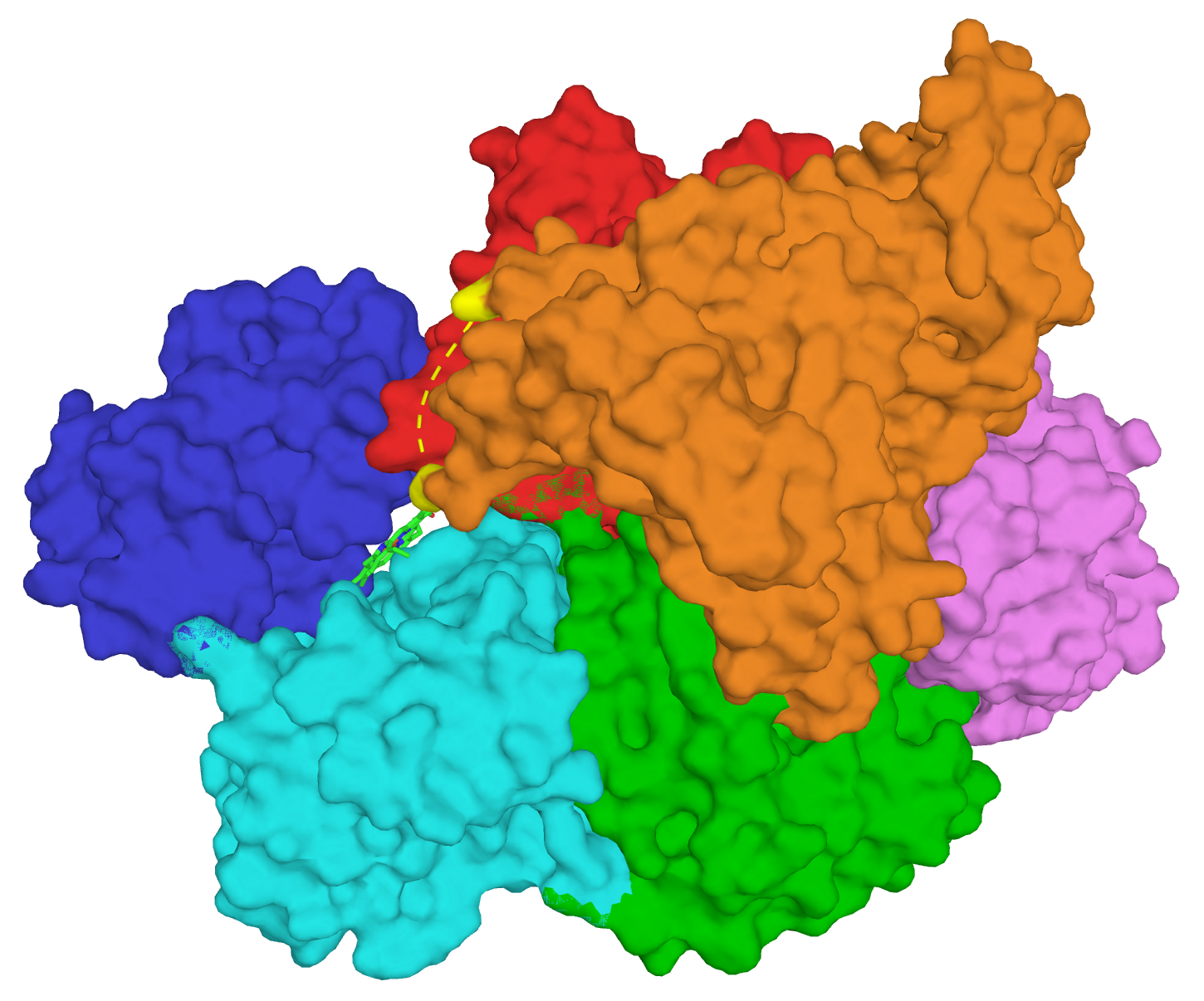

Supplement: Supplementary file 10 — Source data Fig. 4 [file 44318_2025_563_MOESM10_ESM.zip › Fig. 4/Fig 4d/Panel 4D.png]

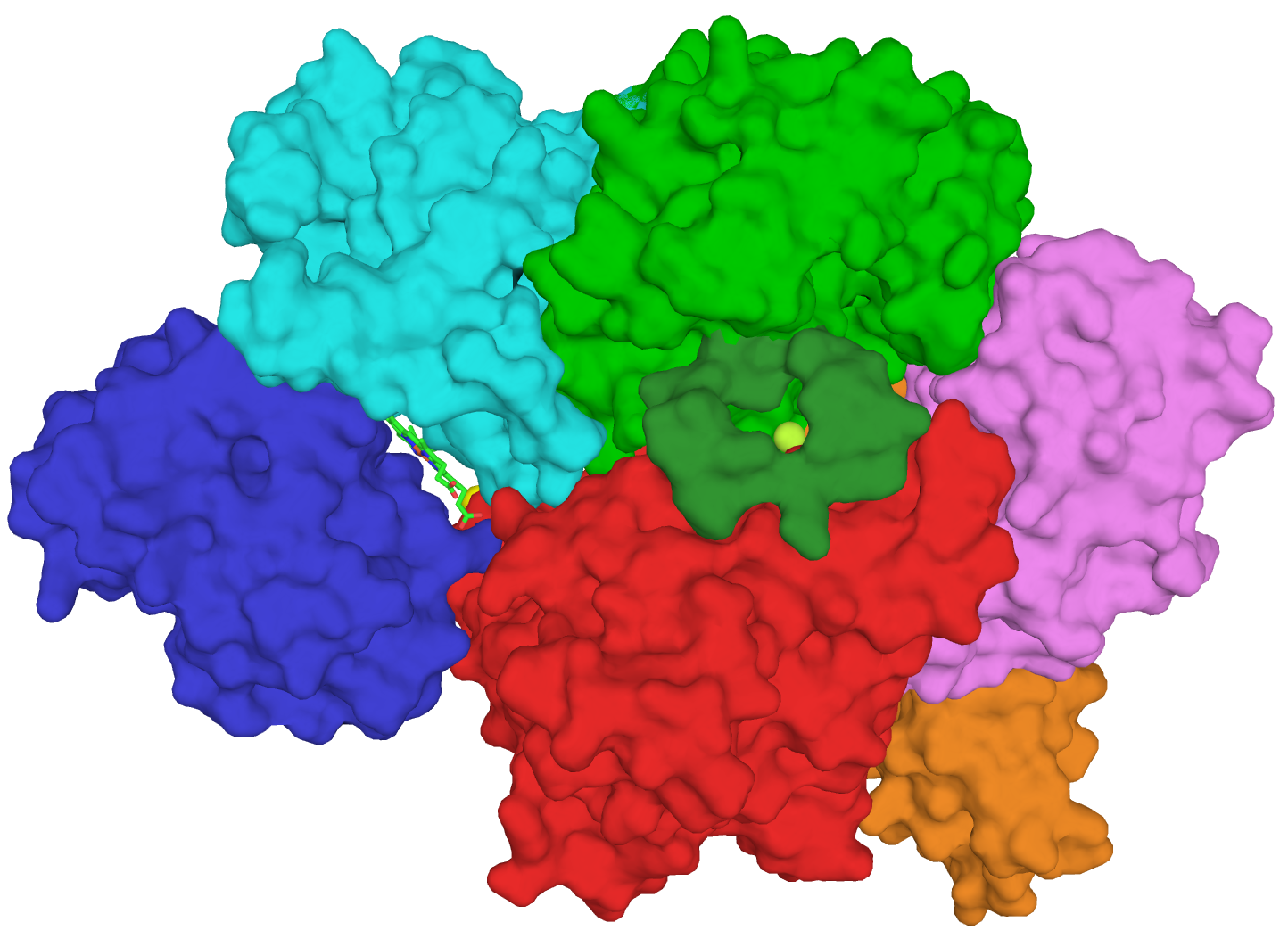

Supplement: Supplementary file 10 — Source data Fig. 4 [file 44318_2025_563_MOESM10_ESM.zip › Fig. 4/Fig 4e/Panel 4E.png]

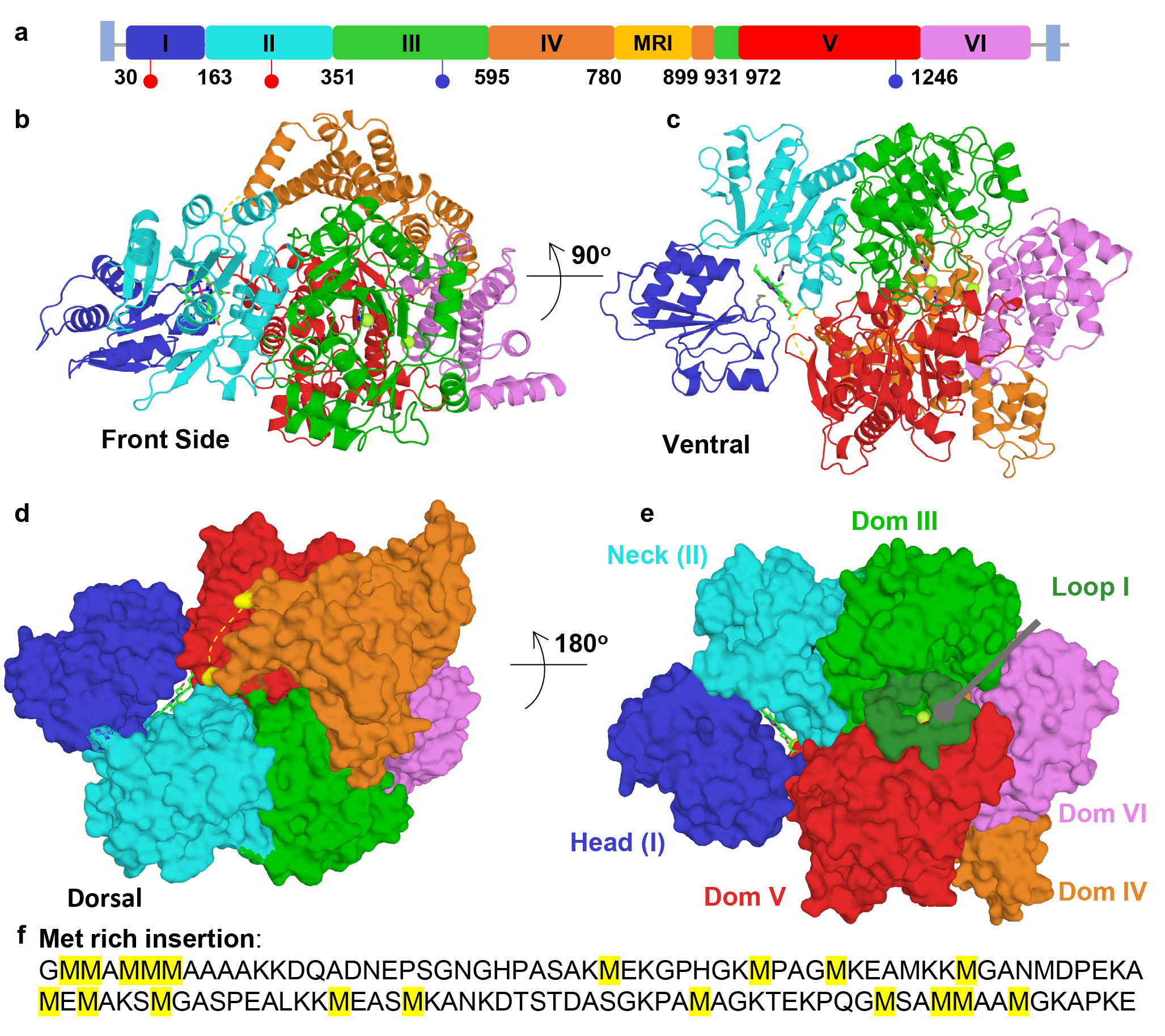

Supplement: Supplementary file 10 — Source data Fig. 4 [file 44318_2025_563_MOESM10_ESM.zip › Fig. 4/Fig_4_300dpi.png]

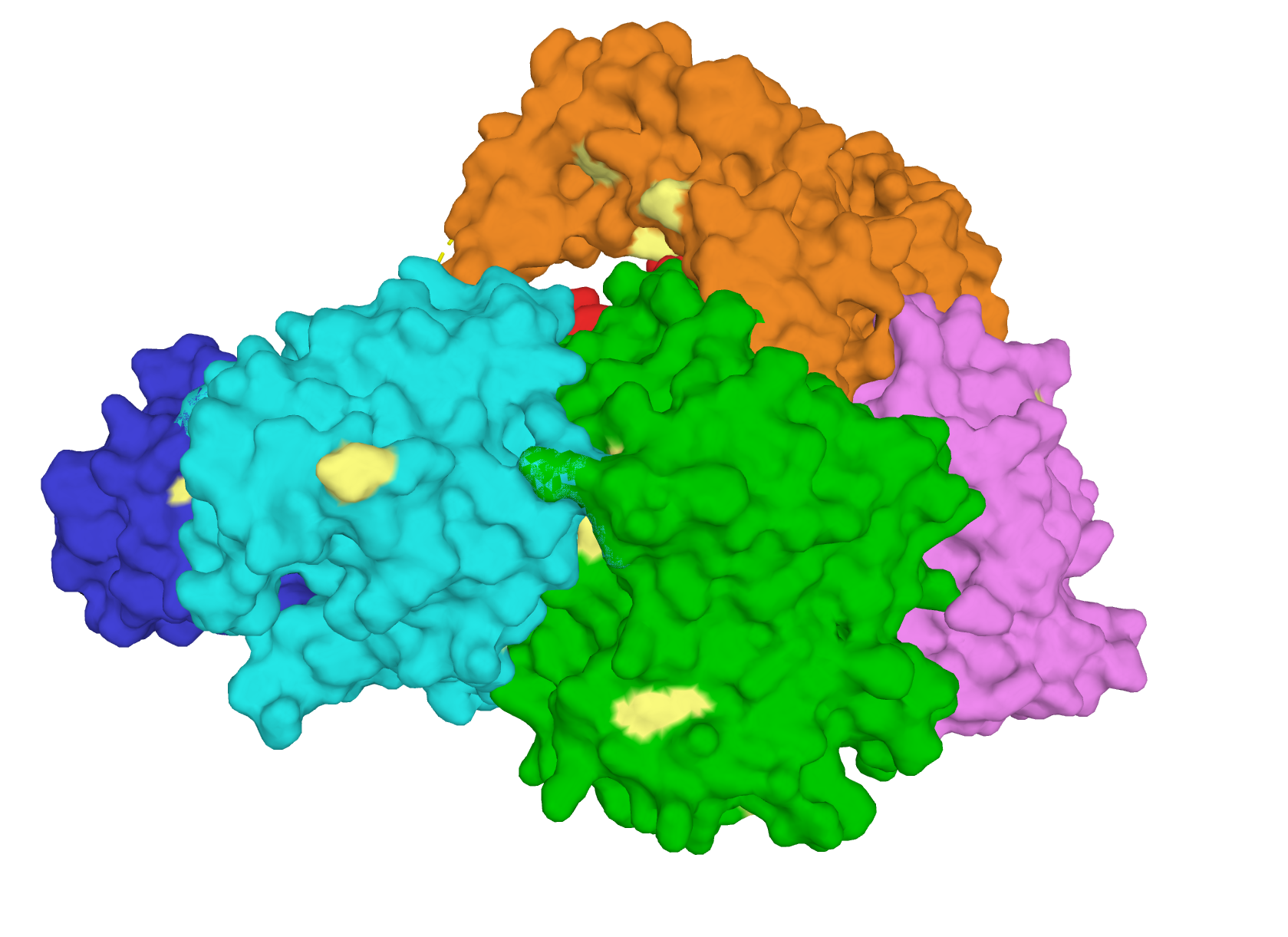

Supplement: Supplementary file 11 — Source data Fig. 5 [file 44318_2025_563_MOESM11_ESM.zip › Fig. 5/Fig 5a/Panel 5A.png]

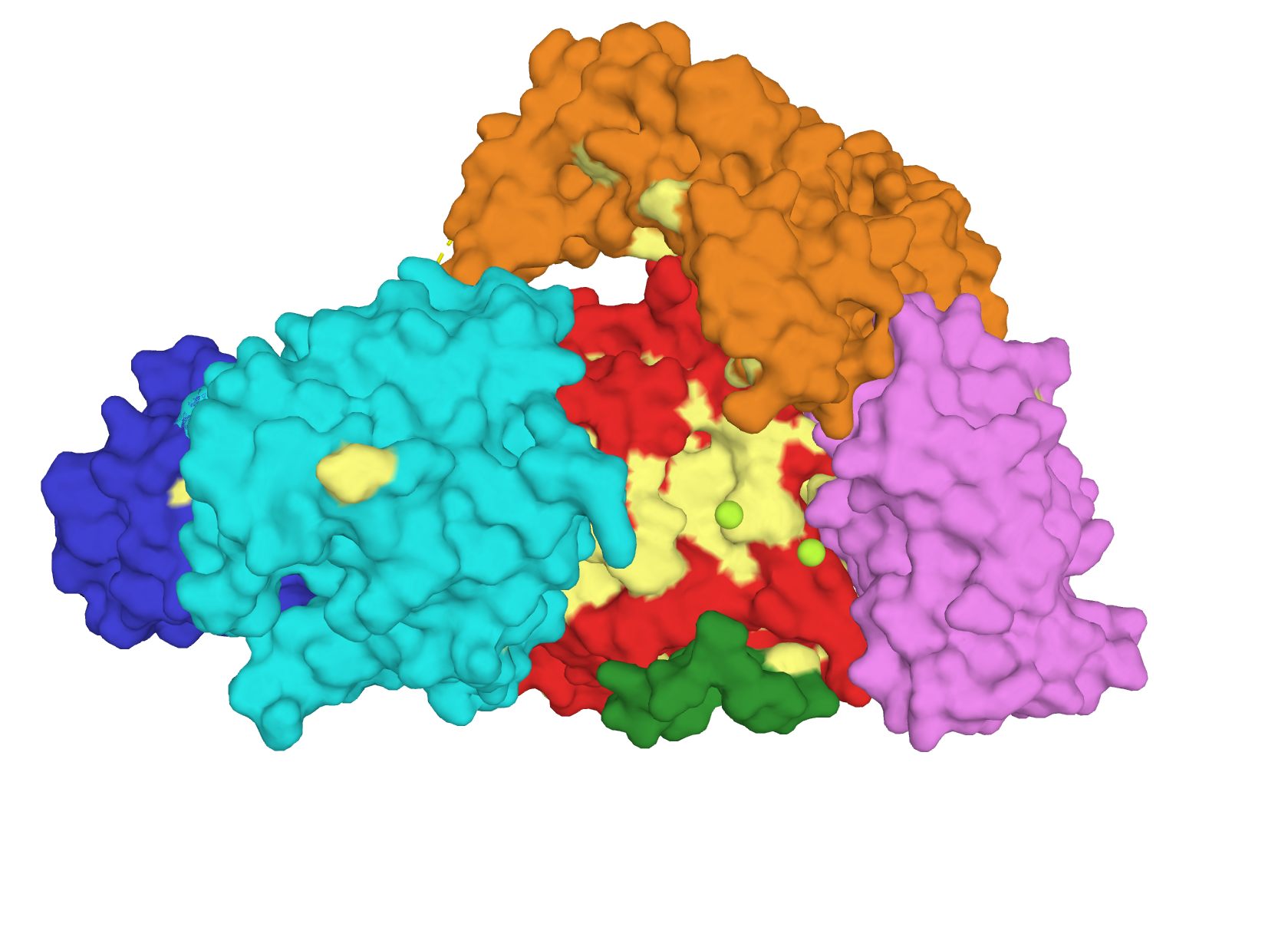

Supplement: Supplementary file 11 — Source data Fig. 5 [file 44318_2025_563_MOESM11_ESM.zip › Fig. 5/Fig 5b/Panel 5B.png]

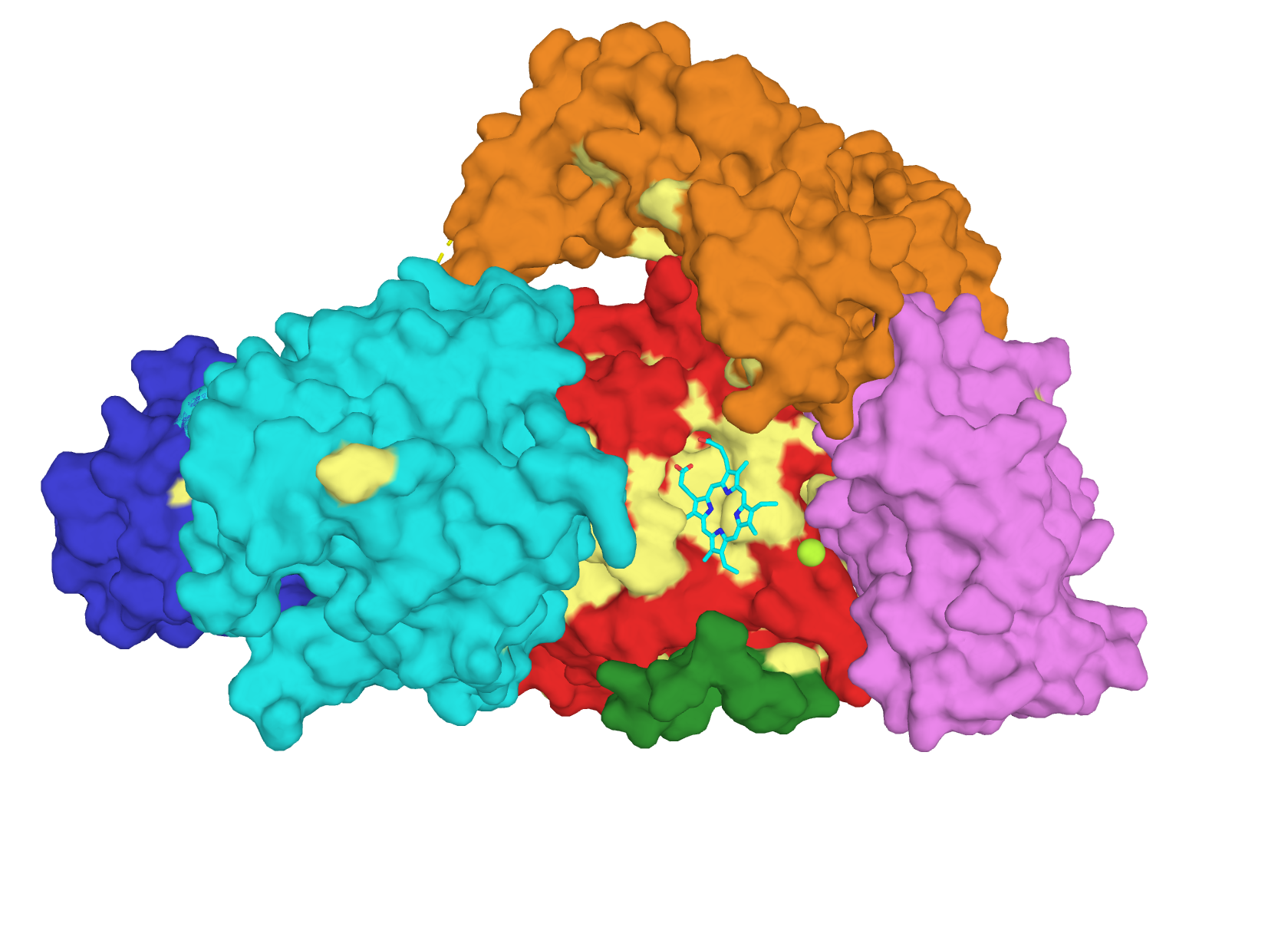

Supplement: Supplementary file 11 — Source data Fig. 5 [file 44318_2025_563_MOESM11_ESM.zip › Fig. 5/Fig 5c/Panel 5C.png]

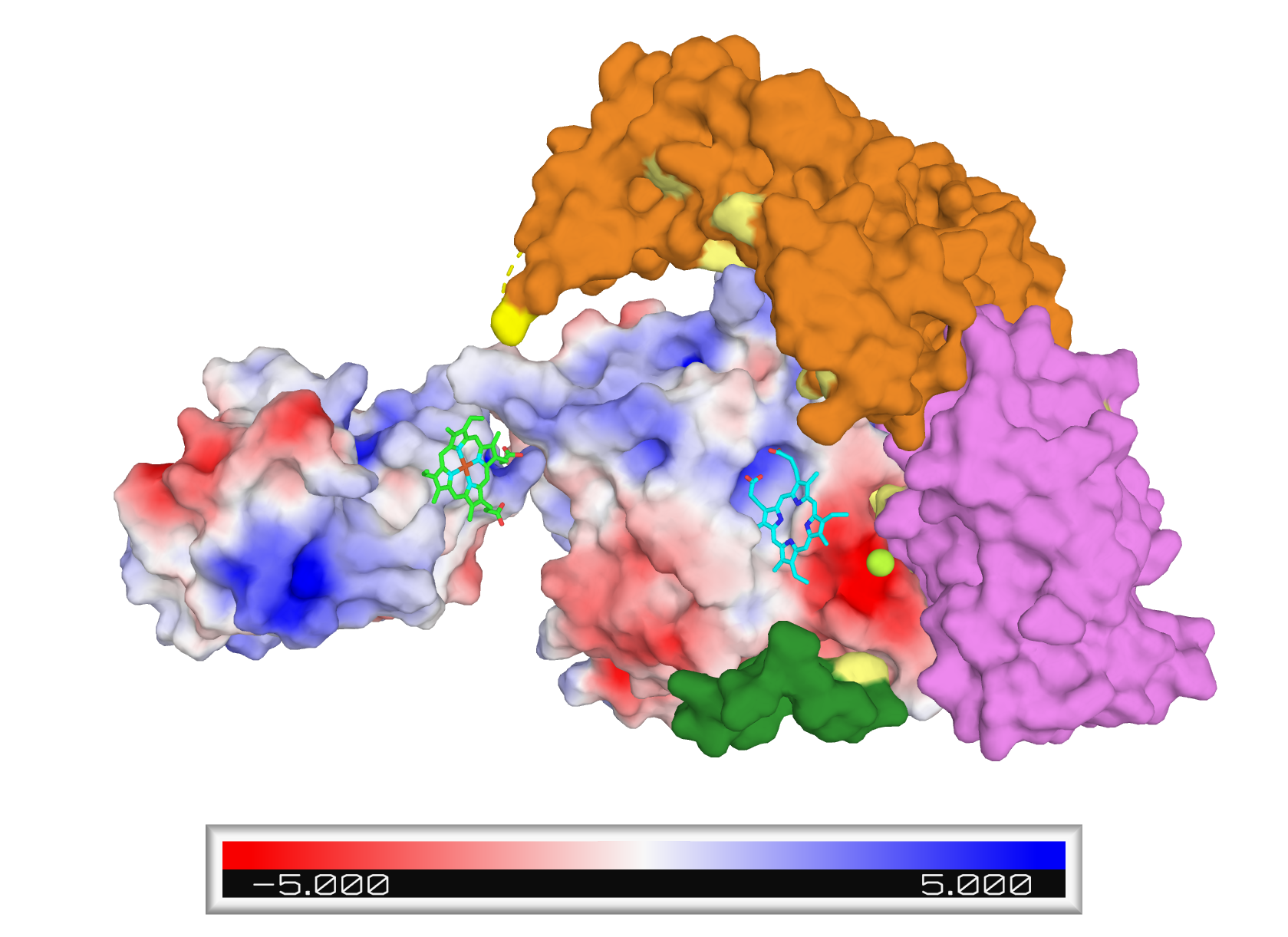

Supplement: Supplementary file 11 — Source data Fig. 5 [file 44318_2025_563_MOESM11_ESM.zip › Fig. 5/Fig 5d/Panel 5D.png]

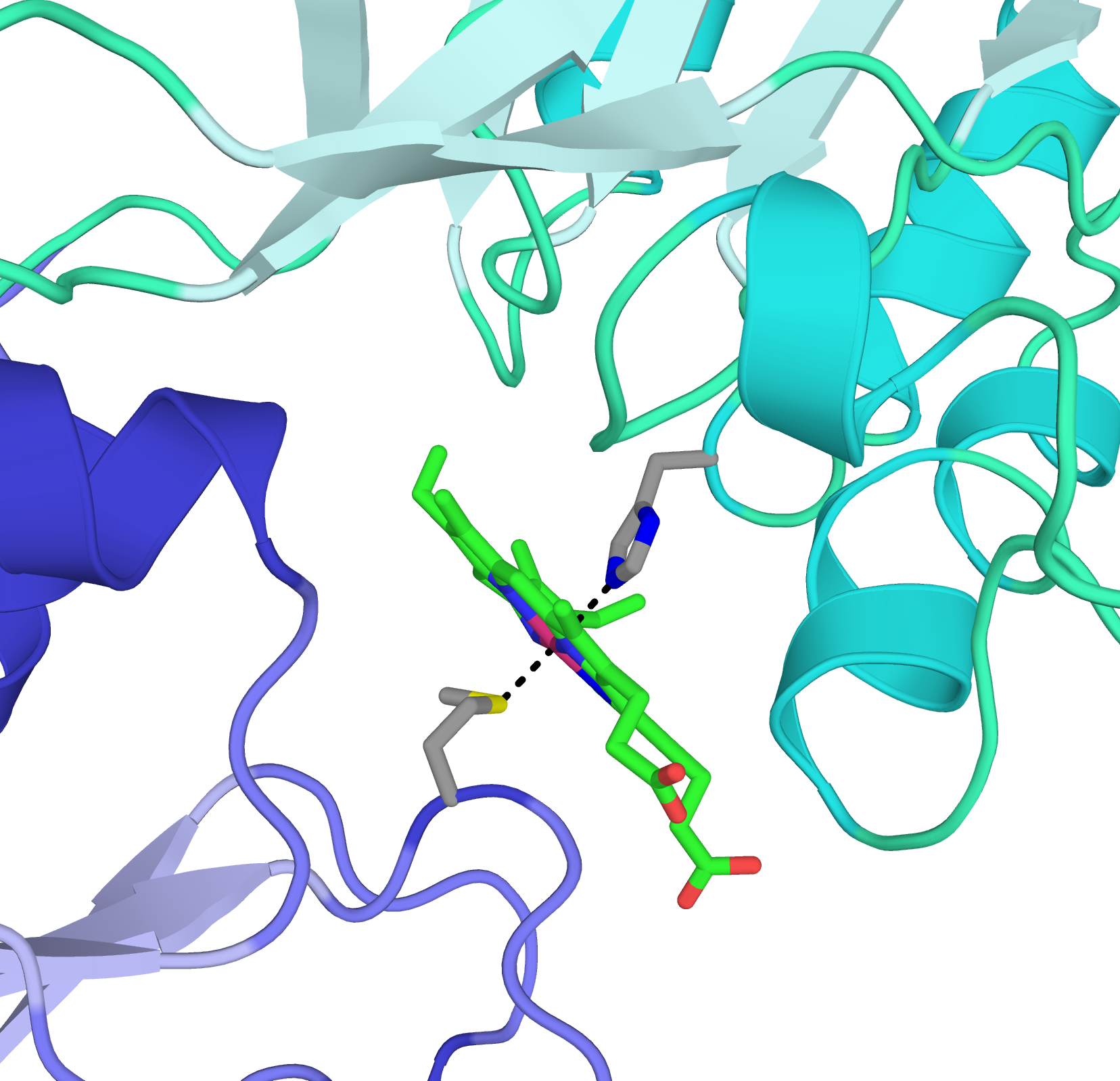

Supplement: Supplementary file 11 — Source data Fig. 5 [file 44318_2025_563_MOESM11_ESM.zip › Fig. 5/Fig 5e/Panel 5E.png]

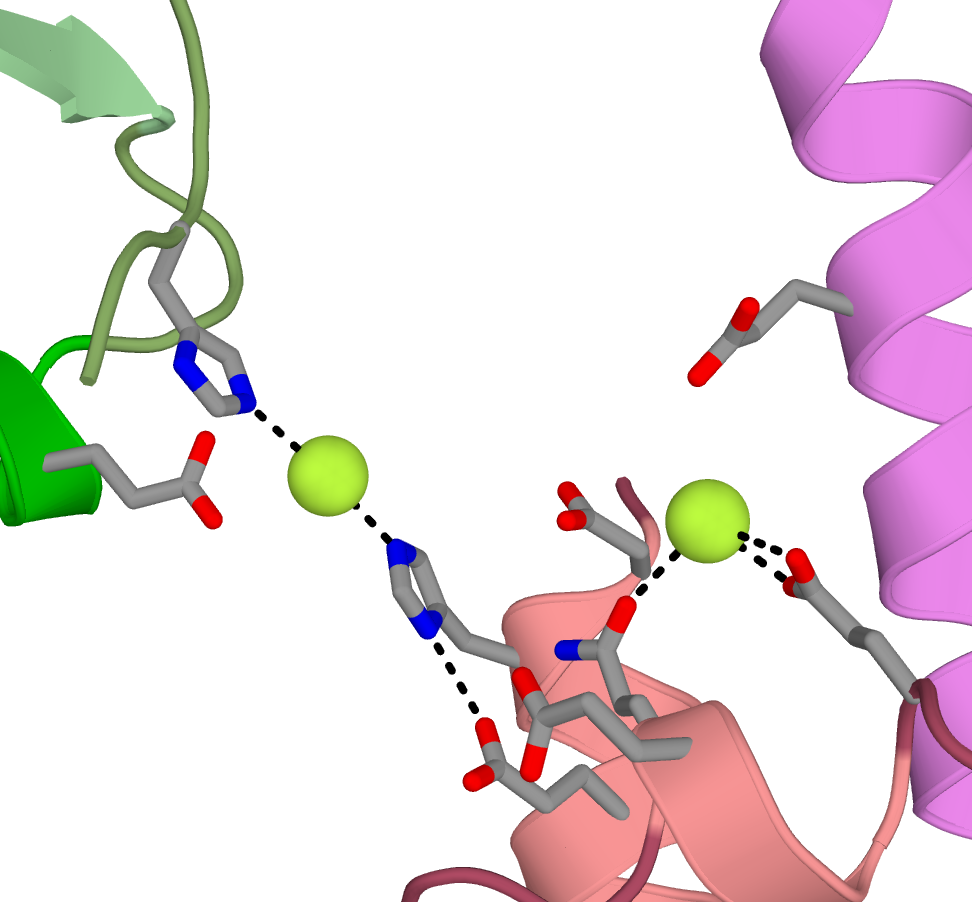

Supplement: Supplementary file 11 — Source data Fig. 5 [file 44318_2025_563_MOESM11_ESM.zip › Fig. 5/Fig 5f/Panel 5F.png]

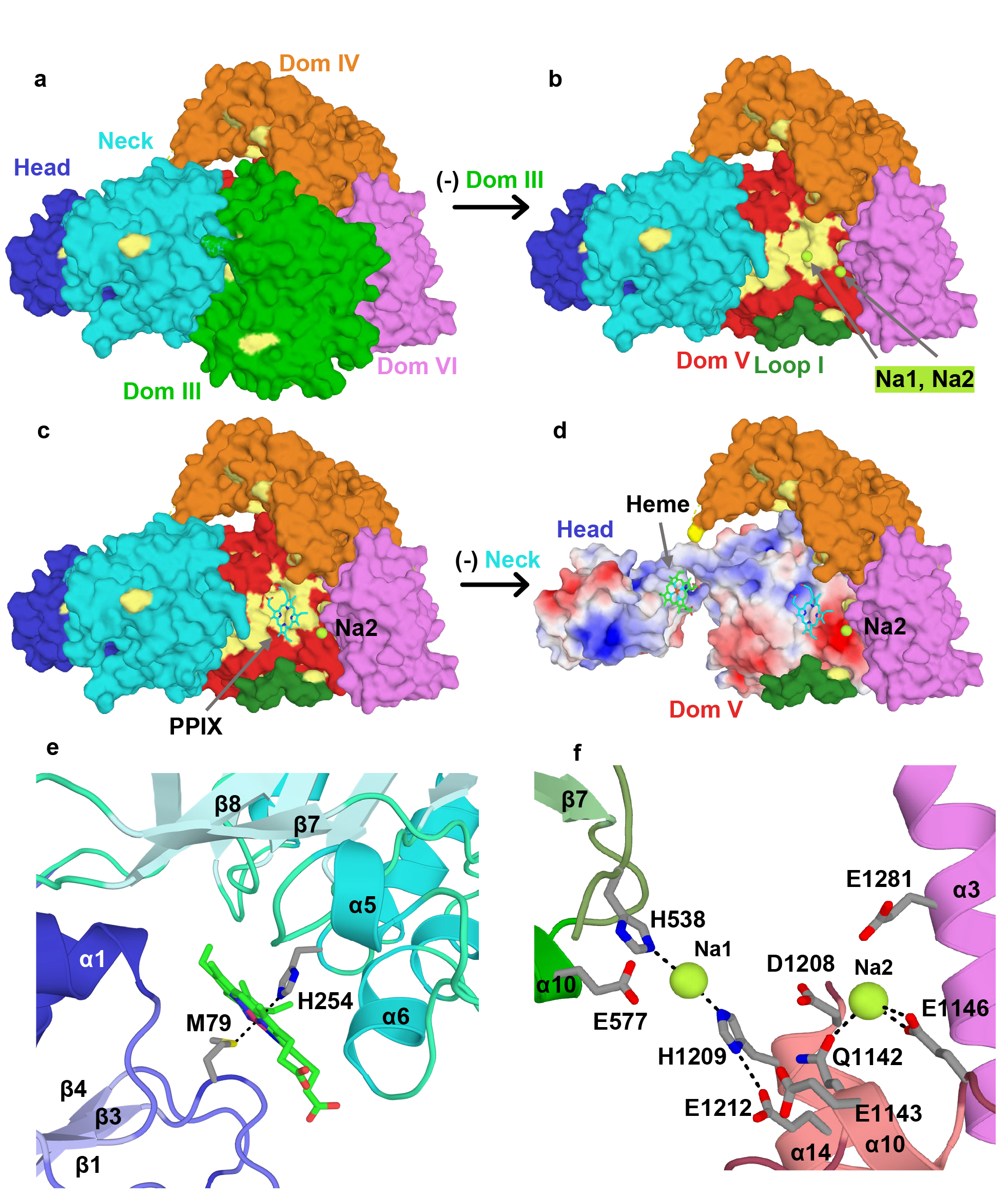

Supplement: Supplementary file 11 — Source data Fig. 5 [file 44318_2025_563_MOESM11_ESM.zip › Fig. 5/Fig_5_300dpi.png]

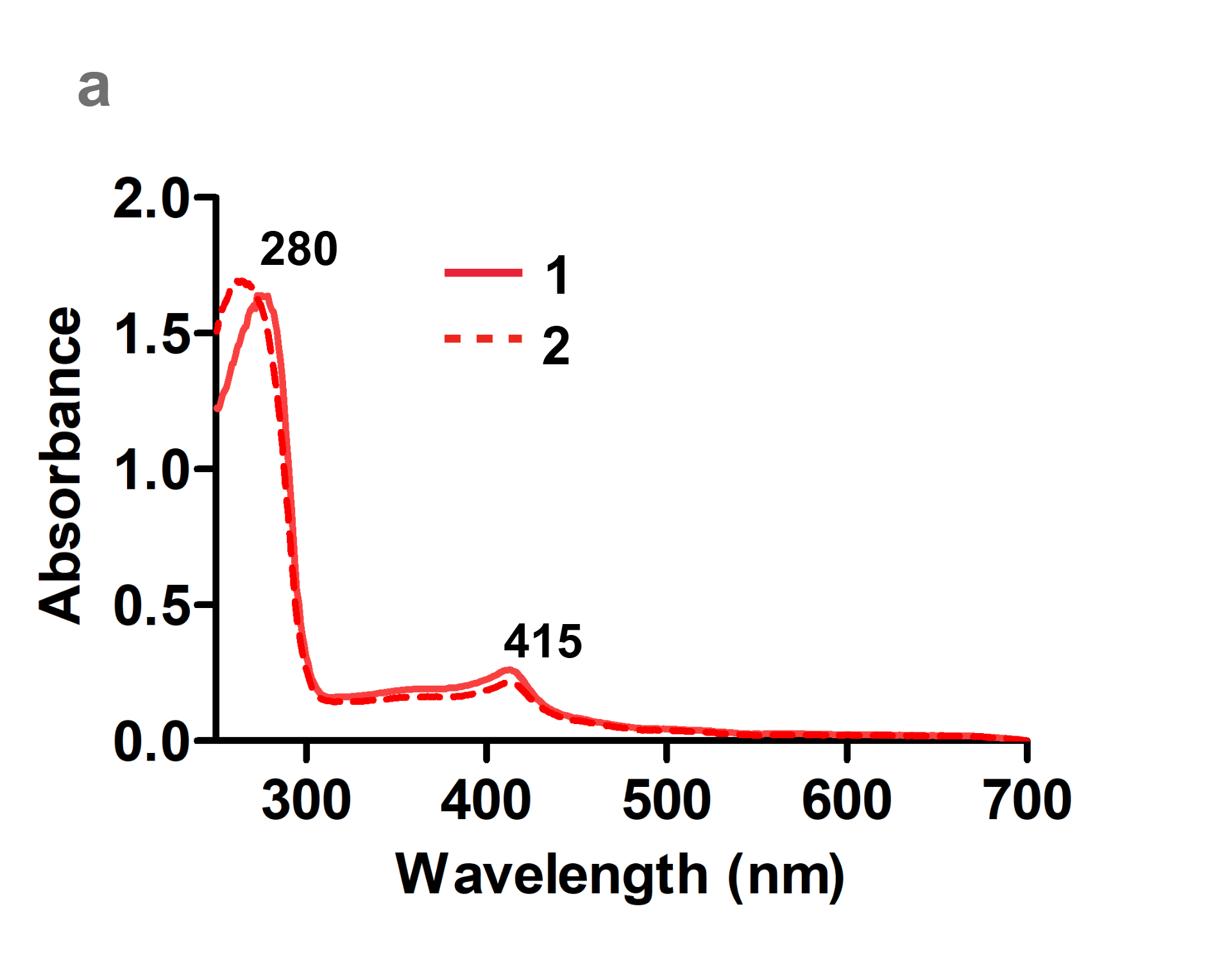

Supplement: Supplementary file 12 — Source data Fig. 6 [file 44318_2025_563_MOESM12_ESM.zip › Fig. 6/Fig 6a/Figure 6a_EMBO.png]

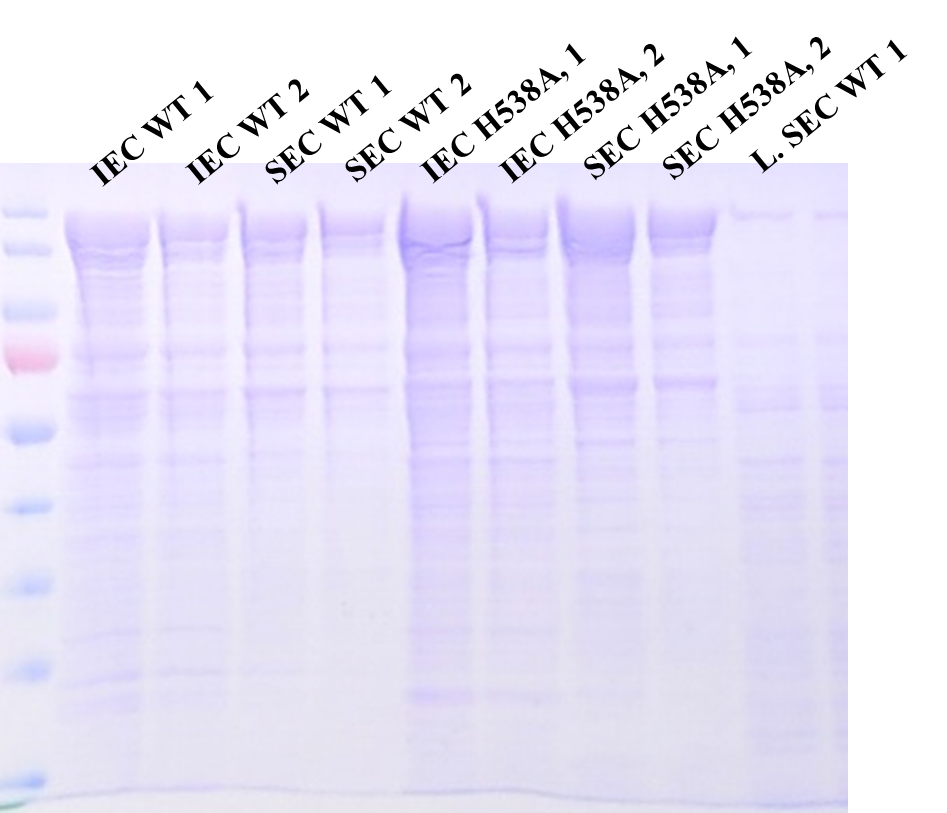

Supplement: Supplementary file 12 — Source data Fig. 6 [file 44318_2025_563_MOESM12_ESM.zip › Fig. 6/Fig 6b-Gel/Fig 6B source image 1.png]

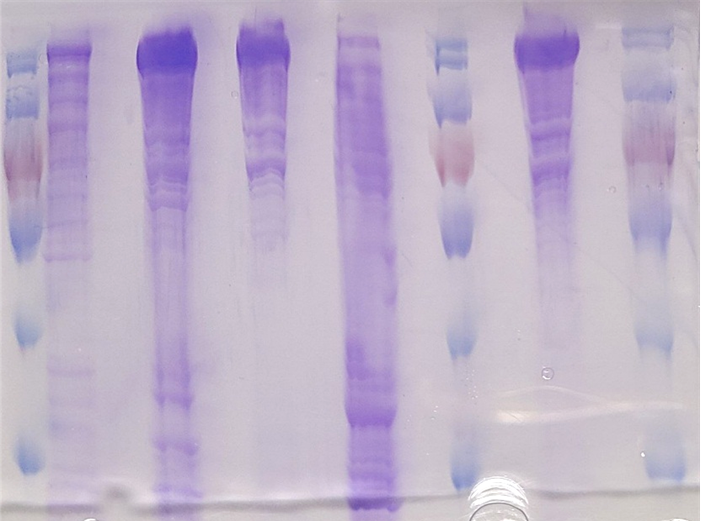

Supplement: Supplementary file 12 — Source data Fig. 6 [file 44318_2025_563_MOESM12_ESM.zip › Fig. 6/Fig 6b-Gel/Fig 6B source image2.png]

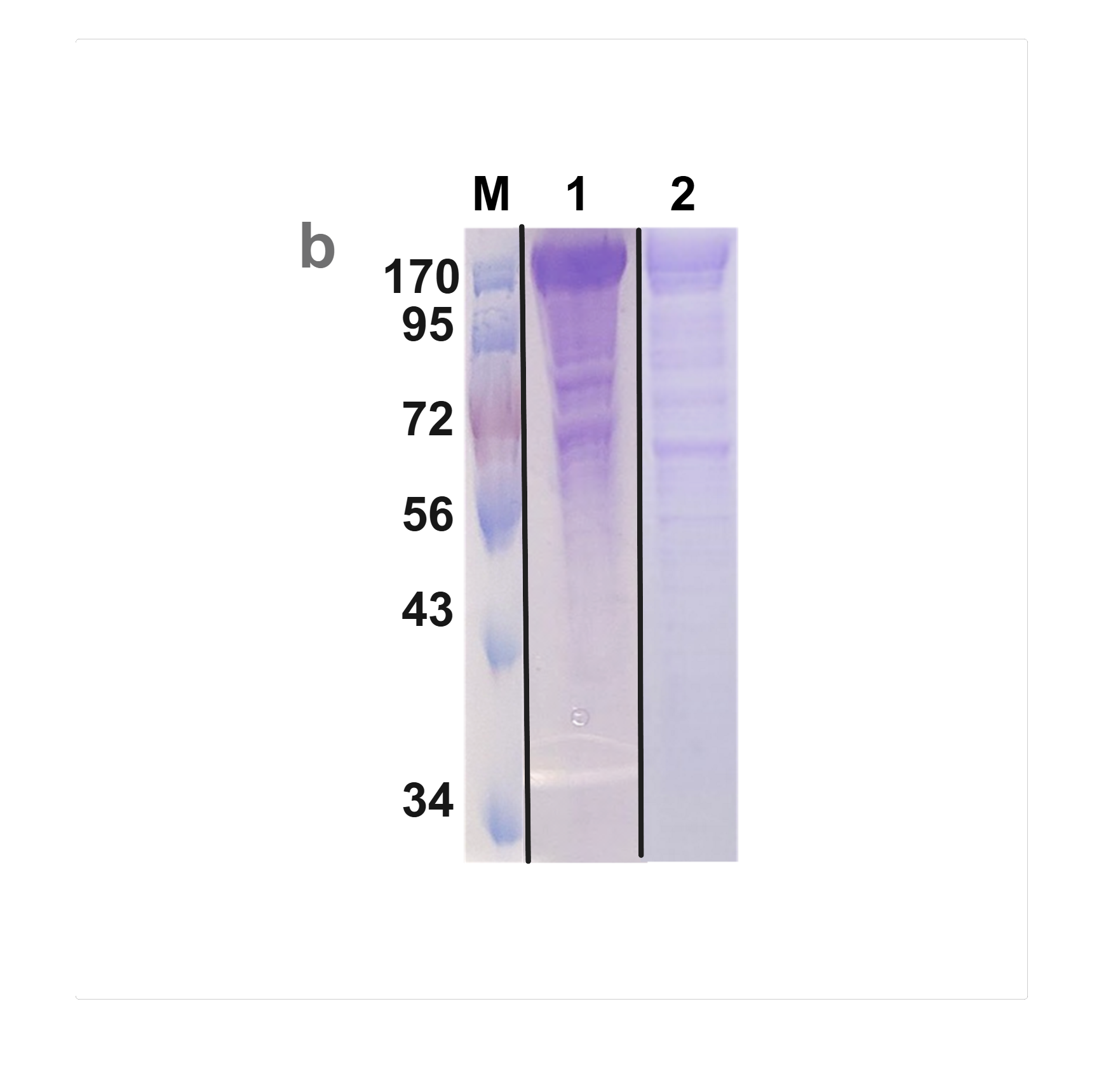

Supplement: Supplementary file 12 — Source data Fig. 6 [file 44318_2025_563_MOESM12_ESM.zip › Fig. 6/Fig 6b-Gel/Figure 6b_EMBO.png]

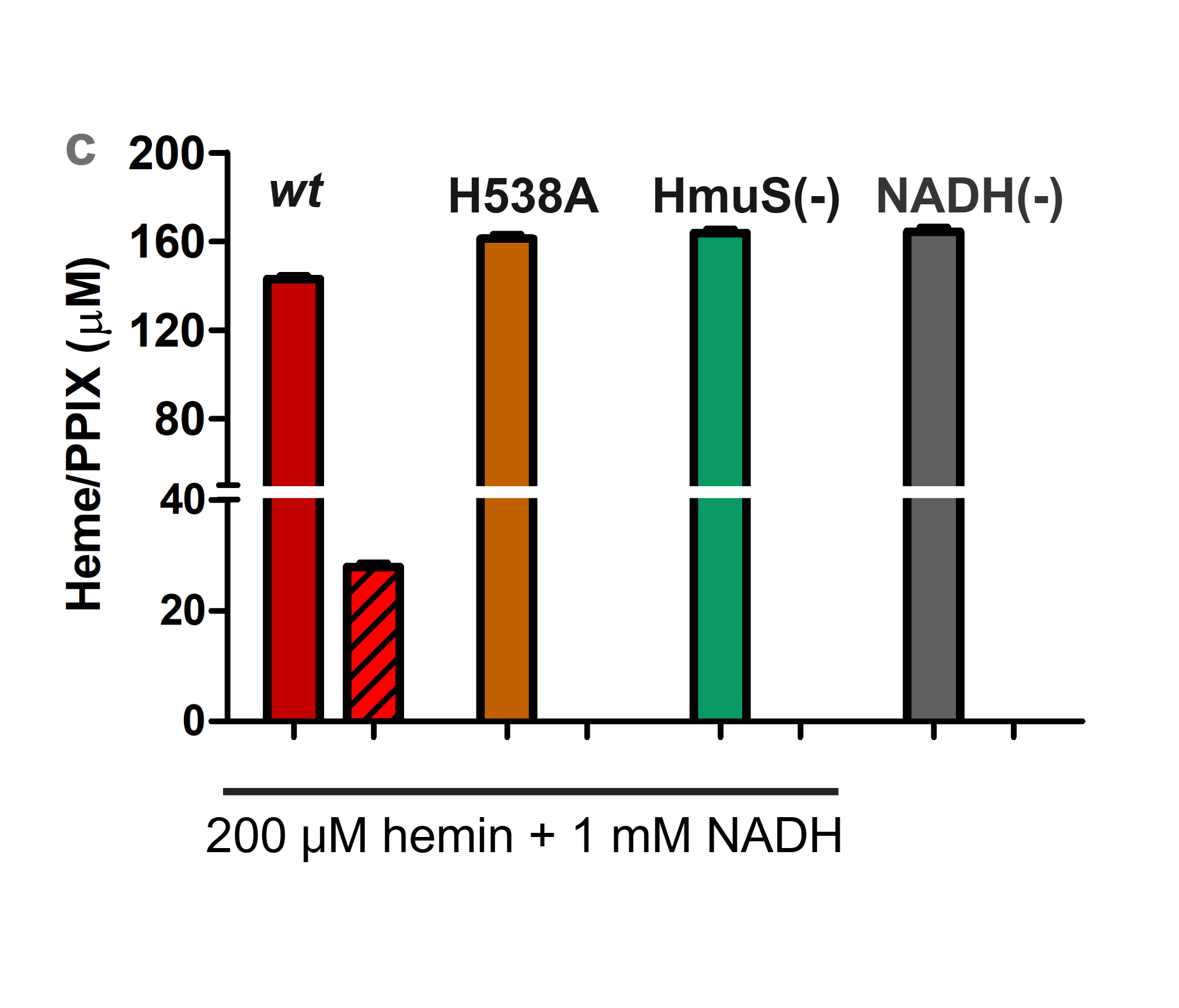

Supplement: Supplementary file 12 — Source data Fig. 6 [file 44318_2025_563_MOESM12_ESM.zip › Fig. 6/Fig 6c/Figure 6c_EMBO.png]

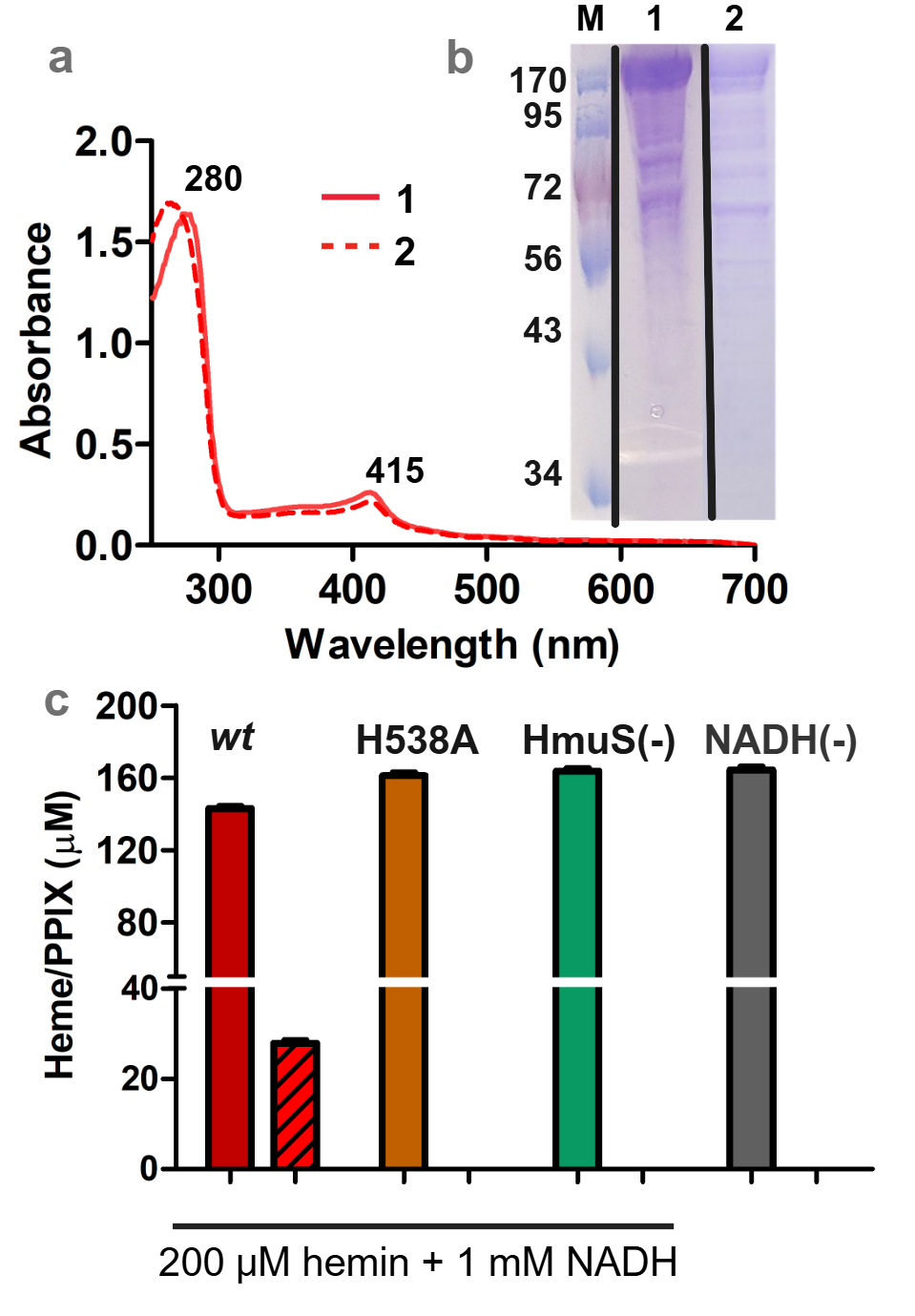

Supplement: Supplementary file 12 — Source data Fig. 6 [file 44318_2025_563_MOESM12_ESM.zip › Fig. 6/Fig6.png]

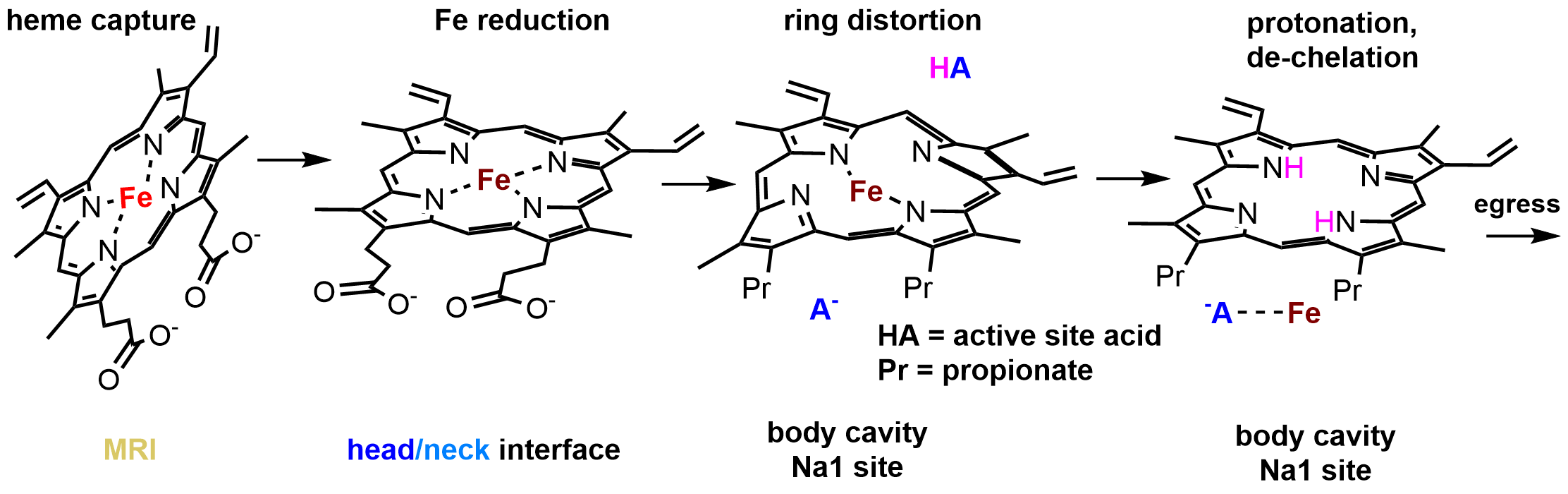

Supplement: Supplementary file 13 — Source data Fig. 7 [file 44318_2025_563_MOESM13_ESM.zip › Fig. 7/Figure 7.tif]

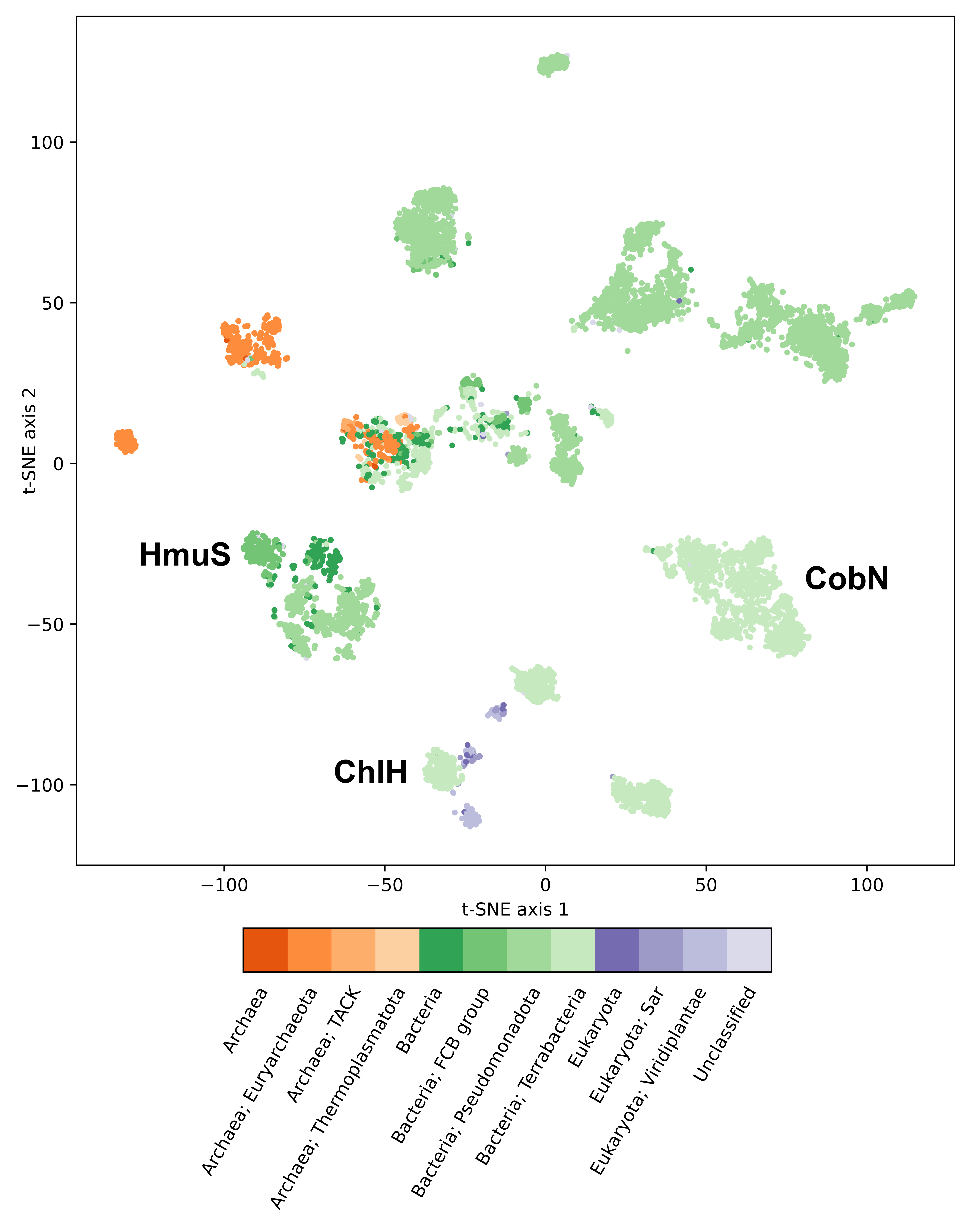

Supplement: Supplementary file 14 — Source data Fig. 8 [file 44318_2025_563_MOESM14_ESM.zip › Fig. 8/Figure_8_vertical.png]

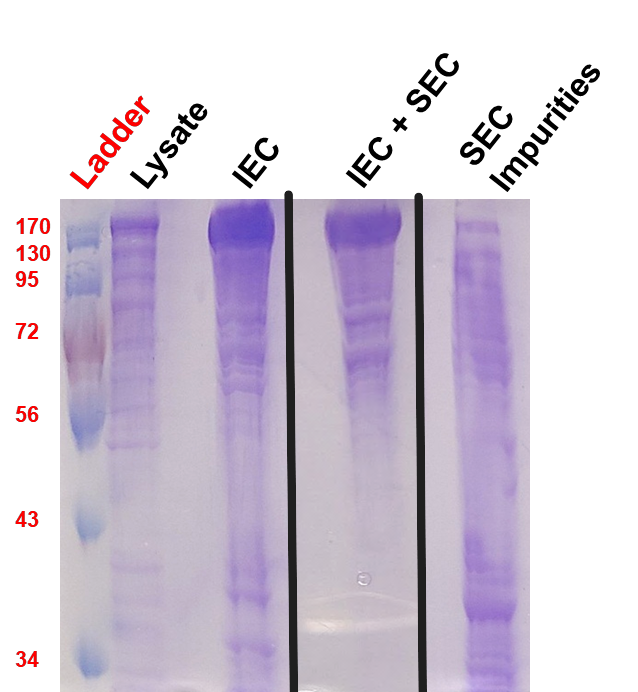

Supplement: Supplementary file 15 — Source data for Appendix Figure S8 [file 44318_2025_563_MOESM15_ESM.zip › Fig. S8/Fig S8 cropped image.png]

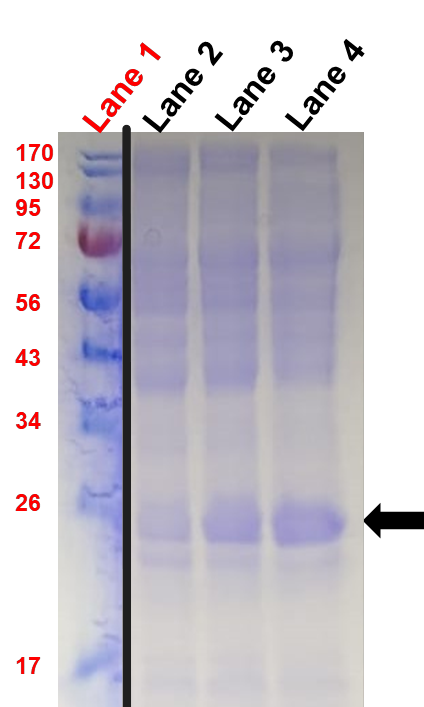

Supplement: Supplementary file 15 — Source data for Appendix Figure S8 [file 44318_2025_563_MOESM15_ESM.zip › Fig. S8/Fig S8D cropped image.png]

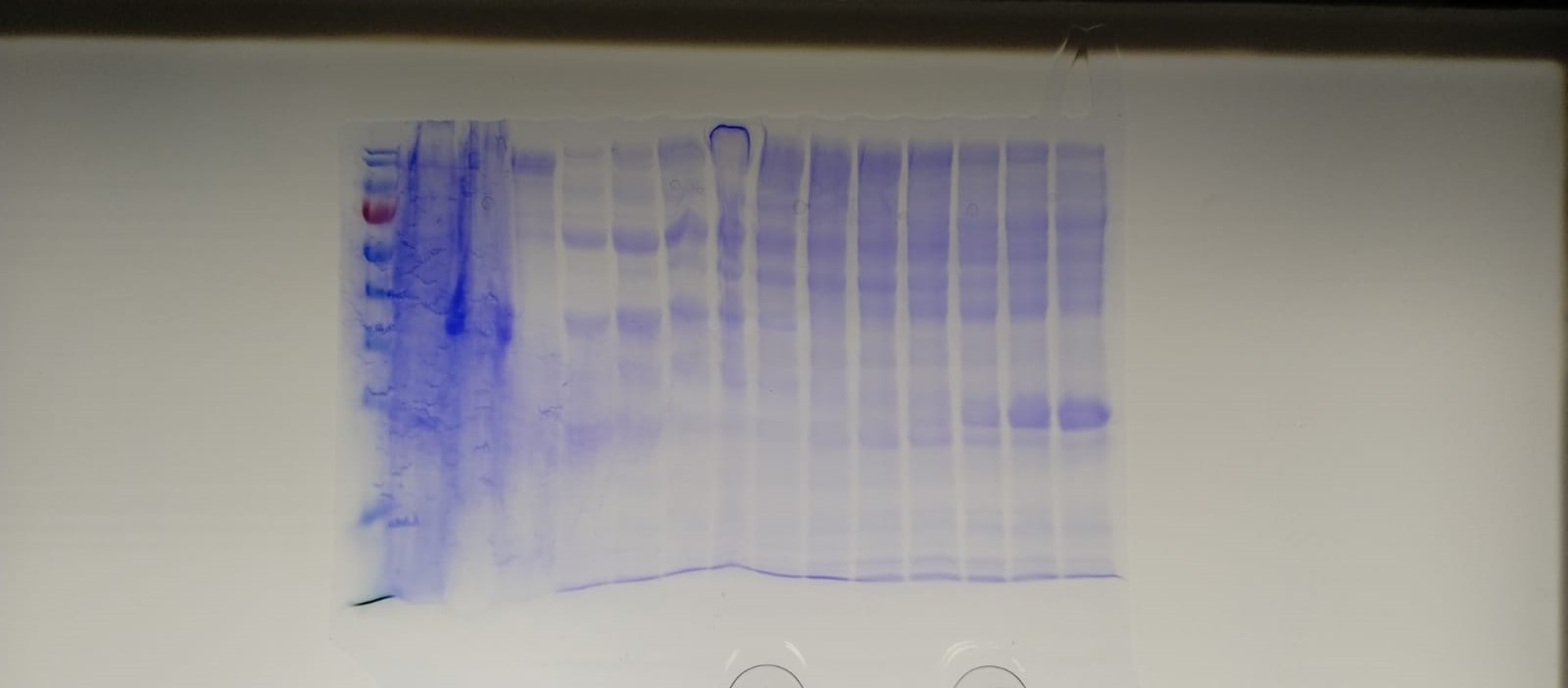

Supplement: Supplementary file 15 — Source data for Appendix Figure S8 [file 44318_2025_563_MOESM15_ESM.zip › Fig. S8/Fig S8D source image.jpg]
