# Supplementary material for: Commensal gut bacteria employ de-chelatase HmuS to harvest iron from heme
Source: EMBO J. 2025 Sep 12;44(21):6226–52. doi: 10.1038/s44318-025-00563-5 (PMC12583661; doi:10.1038/s44318-025-00563-5)
Supplement: Supplementary file 8 — Source data Fig. 2 [file 44318_2025_563_MOESM8_ESM.zip › Fig. 2/Fig 2a/README_Fig2a.docx]

Figure 2a consists of growth curves, plotted as optical density versus time. The raw data are included in the .csv file. Kaleidagraph was used to plot the averaged data points and extrapolate the growth curves.
