# Supplementary material for: Commensal gut bacteria employ de-chelatase HmuS to harvest iron from heme
Source: EMBO J. 2025 Sep 12;44(21):6226–52. doi: 10.1038/s44318-025-00563-5 (PMC12583661; doi:10.1038/s44318-025-00563-5)
Supplement: Supplementary file 8 — Source data Fig. 2 [file 44318_2025_563_MOESM8_ESM.zip › Fig. 2/Raw Data. Fig. 2.docx]

| **Time (h)** | **0 BPS** | | **0.05 mM BPS** | | **0.1 mM BPS** | | **0.3 mM BPS** | |
| --- | --- | --- | --- | --- | --- | --- | --- | --- |
|  | Average | SD | Average | SD | Average | SD | Average | SD |
| **0** | 0.04 | 0 | 0.04 | 0 | 0.04 | 0 | 0.04 | 0 |
| **2** | 0.0635 | 0.002121 | 0.068 | 0.005657 | 0.07 | 0.001414 | 0.0645 | 0.002121 |
| **4** | 0.0955 | 0.006364 | 0.0925 | 0.013435 | 0.0975 | 0.006364 | 0.089 | 0.008485 |
| **6** | 0.171 | 0.004243 | 0.15825 | 0.019445 | 0.164 | 0.003536 | 0.1325 | 0.008485 |
| **8** | 0.2465 | 0.002121 | 0.224 | 0.025456 | 0.2305 | 0.000707 | 0.176 | 0.008485 |
| **10** | 0.3175 | 0.003536 | 0.2825 | 0.033234 | 0.286 | 0.002828 | 0.206 | 0.008485 |
| **12** | 0.4855 | 0.000707 | 0.4025 | 0.038891 | 0.3975 | 0.003536 | 0.245 | 0.014142 |
| **14** | 0.7115 | 0.016263 | 0.59 | 0.042426 | 0.5585 | 0.006364 | 0.279 | 0.012728 |
| **16** | 1.045 | 0.005657 | 0.862 | 0.045255 | 0.8105 | 0.013435 | 0.3465 | 0.026163 |
| **18** | 1.17 | 0.011314 | 1.035 | 0.035355 | 1.005 | 0.007071 | 0.4345 | 0.024749 |
| **20** | 1.28 | 0 | 1.1555 | 0.012021 | 1.1435 | 0.010607 | 0.647 | 0.009899 |
| **22** | 1.31 | 0 | 1.164 | 0 | 1.151 | 0 | 0.885 | 0.021213 |
| **24** | 1.34 | 0 | 1.147 | 0 | 1.136 | 0 | 1.01 | 0.028284 |
| **26** | 1.34 | 0 | 1.1555 | 0.012021 | 1.151 | 0 | 1.01 | 0.028284 |

**Figure 2a.** Growth curve, A_600nm_

**Figure 2b.** Heme and PPIX quantification, HPLC analysis

|  | **0 BPS** | | **0.05 mM BPS** | | **0.1 mM BPS** | | **0.3 mM BPS** | |
| --- | --- | --- | --- | --- | --- | --- | --- | --- |
|  | Average | SD | Average | SD | Average | SD | Average | SD |
| **Heme (µM)** | 40.2634 | 0.89232633 | 32.063745 | 0.364365 | 44.5191 | 2.774277 | 45.376455 | 0.476243 |
| **PPIX (µM)** | 0.034751 | 0.0057554 | 0.4603405 | 0.00087 | 0.8968022 | 0.078554 | 2.565432 | 0.154282 |

**Figure 2c.** Growth curve (wild type and *hmuS* mutants), A_600nm_

|  |  | | **Haem + Fe^2+^** | |  | | |  | | **Haem** | |  | |
| --- | --- | --- | --- | --- | --- | --- | --- | --- | --- | --- | --- | --- | --- |
| **Time (h)** | ***hmuS* (-) BT0494** | | ***hmuS* (-) BT0495** | | ***Wt*** | | | ***hmuS* (-) BT0494** | | ***hmuS* (-) BT0495** | | ***Wt*** | |
|  | Average | SD | Average | SD | Average | SD |  | Average | SD | Average | SD | Average | SD |
| **0** | 0.035 | 0 | 0.035 | 0 | 0.035 | 0 |  | 0.035 | 0.000 | 0.035 | 0.000 | 0.035 | 0.000 |
| **2** | 0.122 | 0 | 0.092 | 0 | 0.091 | 0 |  | 0.12 | 0.003 | 0.0985 | 0.008 | 0.103 | 0.001 |
| **4** | 0.167 | 0 | 0.135 | 0 | 0.134 | 0 |  | 0.1455 | 0.005 | 0.1255 | 0.012 | 0.1365 | 0.001 |
| **6** | 0.285 | 0 | 0.211 | 0 | 0.231 | 0 |  | 0.19 | 0.003 | 0.157 | 0.008 | 0.2075 | 0.001 |
| **8** | 0.394 | 0 | 0.282 | 0 | 0.334 | 0 |  | 0.2105 | 0.005 | 0.1735 | 0.009 | 0.2605 | 0.005 |
| **10** | 0.555 | 0 | 0.382 | 0 | 0.483 | 0 |  | 0.2265 | 0.004 | 0.1835 | 0.009 | 0.331 | 0.001 |
| **12** | 0.8385 | 0 | 0.676 | 0 | 0.63475 | 0 |  | 0.233 | 0.000 | 0.18675 | 0.002 | 0.42325 | 0.031 |
| **14** | 0.98 | 0 | 0.82 | 0 | 0.94 | 0 |  | 0.253 | 0.000 | 0.2 | 0.000 | 0.6 | 0.014 |
| **16** | 1.122 | 0 | 0.97 | 0 | 1.09 | 0 |  | 0.237 | 0.052 | 0.2105 | 0.015 | 0.79 | 0.003 |
| **18** | 1.17575 | 0 | 1.0595 | 0 | 1.15375 | 0 |  | 0.2565 | 0.037 | 0.2185 | 0.012 | 0.874875 | 0.001 |
| **20** | 1.2295 | 0 | 1.149 | 0 | 1.2175 | 0 |  | 0.276 | 0.023 | 0.2265 | 0.009 | 0.95975 | 0.000 |
| **22** | 1.28325 | 0 | 1.2385 | 0 | 1.28125 | 0 |  | 0.2955 | 0.008 | 0.2345 | 0.006 | 1.044625 | 0.002 |
| **24** | 1.337 | 0 | 1.328 | 0 | 1.345 | 0 |  | 0.315 | 0.007 | 0.2425 | 0.004 | 1.1295 | 0.004 |
| **26** | 1.3355 | 0 | 1.317 | 0 | 1.34 | 0 |  | 0.314 | 0.008 | 0.2495 | 0.008 | 1.1295 | 0.004 |

**Figure 2d.** Growth curve (wild type and *hmuS* mutants) serial passages - inoculum, A_600nm_

|  |  | | **Haem + Fe^2+^** | |  | | |  | | **Fe^2+^** | |  | |
| --- | --- | --- | --- | --- | --- | --- | --- | --- | --- | --- | --- | --- | --- |
| **Time (h)** | ***hmuS* (-) BT0494** | | ***hmuS* (-) BT0495** | | ***Wt*** | |  | ***hmuS* (-) BT0494** | | ***hmuS* (-) BT0495** | | ***Wt*** | |
|  | Average | SD | Average | SD | Average | SD |  | Average | SD | Average | SD | Average | SD |
| **0** | 0.045 | 0.0000 | 0.0345 | 0.0035 | 0.042 | 0.0071 |  | 0.034667 | 0.0021 | 0.026667 | 0.0025 | 0.033 | 0.0040 |
| **2** | 0.0615 | 0.0049 | 0.046 | 0.0042 | 0.068 | 0.0071 |  | 0.047 | 0.0010 | 0.039333 | 0.0025 | 0.046667 | 0.0015 |
| **4** | 0.09875 | 0.0025 | 0.074 | 0.0035 | 0.11875 | 0.0095 |  | 0.054333 | 0.0013 | 0.046 | 0.0023 | 0.056167 | 0.0006 |
| **6** | 0.136 | 0.0000 | 0.102 | 0.0028 | 0.1695 | 0.0120 |  | 0.061667 | 0.0015 | 0.052667 | 0.0038 | 0.065667 | 0.0006 |
| **8** | 0.207 | 0.0000 | 0.1425 | 0.0035 | 0.268 | 0.0113 |  | 0.067 | 0.0035 | 0.060333 | 0.0084 | 0.072667 | 0.0021 |
| **10** | 0.34 | 0.0141 | 0.2115 | 0.0035 | 0.485 | 0.0212 |  | 0.074333 | 0.0059 | 0.071333 | 0.0153 | 0.081333 | 0.0023 |
| **12** | 0.57 | 0.0141 | 0.325 | 0.0071 | 0.815 | 0.0212 |  | 0.08 | 0.0087 | 0.083 | 0.0260 | 0.089667 | 0.0029 |
| **14** | 0.906 | 0.0226 | 0.5195 | 0.0163 | 1.118 | 0.0042 |  | 0.087333 | 0.0127 | 0.095 | 0.0349 | 0.096667 | 0.0031 |
| **16** | 1.14 | 0.0057 | 0.867 | 0.0255 | 1.2745 | 0.0078 |  | 0.093667 | 0.0142 | 0.109 | 0.0546 | 0.100333 | 0.0032 |
| **26** | 1.3695 | 0.0035 | 1.327 | 0.0156 | 1.377 | 0.0156 |  | 0.129333 | 0.0405 | 0.104 | 0.0028 | 0.135 | 0.0026 |

**Figure 2e.** Heme and PPIX quantification, HPLC analysis. Reaction of *B. theta* cell lysate supplemented with ATP and NADH

| **Lysate** | | | **Dialyzed lysate** | | **Dialyzed lysate + NADH** | | **Dialyzed lysate + ATP/NADH** | | **Dialyzed lysate + ATP** | |
| --- | --- | --- | --- | --- | --- | --- | --- | --- | --- | --- |
|  | Average | SD | Average | SD | Average | SD | Average | SD | Average | SD |
| **Heme (µM)** | 29.84366 | 1.482093 | 31.47674 | 0.582126 | 11.47139 | 0.238962 | 12.90059 | 0.594971 | 30.23626 | 0.710058 |
| **PPIX (µM)** | 5.236034 | 0.800628 | 4.591893 | 0.309348 | 28.98057 | 0.78362 | 26.36537 | 0.264013 | 0 | 0 |

**Figure 2f.** Heme and PPIX quantification, HPLC analysis. Reaction of *B. theta* cell lysate fractions

|  | **Membrane fraction** | | **Soluble fraction** | |
| --- | --- | --- | --- | --- |
|  | Average | SD | Average | SD |
| **Heme (µM)** | 8.268725 | 0.38521 | 22.47204 | 1.893227 |
| **PPIX (µM)** | 35.62301 | 0.241868 | 12.80948 | 1.529216 |
