# Supplementary material for: Commensal gut bacteria employ de-chelatase HmuS to harvest iron from heme
Source: EMBO J. 2025 Sep 12;44(21):6226–52. doi: 10.1038/s44318-025-00563-5 (PMC12583661; doi:10.1038/s44318-025-00563-5)
Supplement: Supplementary file 9 — Source data Fig. 3 [file 44318_2025_563_MOESM9_ESM.zip › Fig. 3/Fig 3a/README_Fig3a.docx]

Figure 3a shows 3 UV/visible absorbance spectra numbered 1-3. Kaleidagraph was used to plot the data, which are representative of the spectra we routinely measure for these protein fractions.
