# Supplementary material for: Commensal gut bacteria employ de-chelatase HmuS to harvest iron from heme
Source: EMBO J. 2025 Sep 12;44(21):6226–52. doi: 10.1038/s44318-025-00563-5 (PMC12583661; doi:10.1038/s44318-025-00563-5)
Supplement: Supplementary file 9 — Source data Fig. 3 [file 44318_2025_563_MOESM9_ESM.zip › Fig. 3/Fig 3b-Gel/README_Fig3b.docx]

Figure 3b shows an SDS PAGE image that has been cropped to emphasize a molecular weight size marker and the same three protein fractions (1-3) for which spectra are provided in Figure 3a. Aside from cropping, the image is unaltered. It was taken using a cell phone camera and a gel placed on a visible light table.


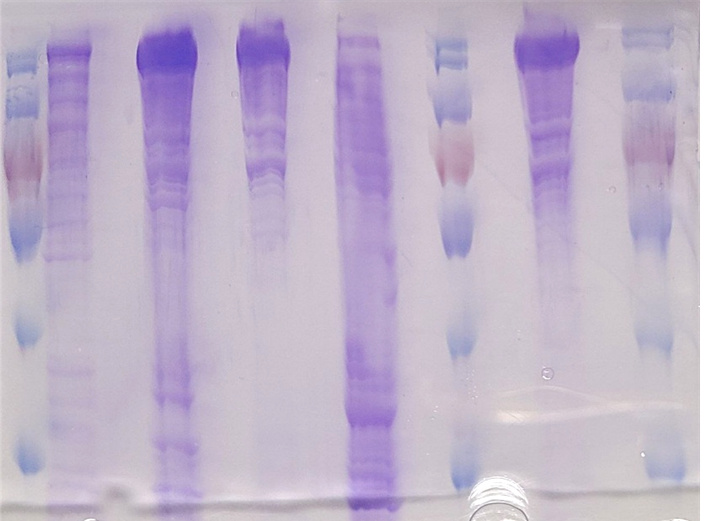


Lanes 1, 2, and 7 were combined on the image below:


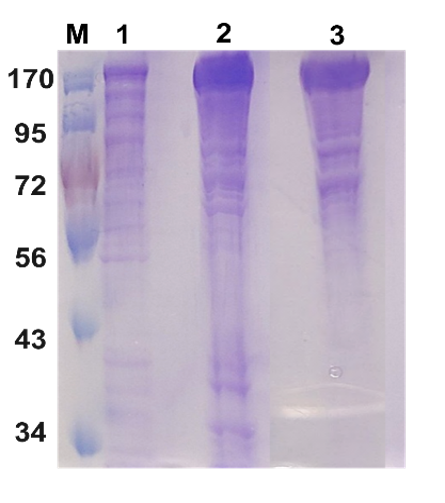


A black line has been inserted between lanes marked 2 and 3 to indicate the location of the discontinuity.
