# Supplementary material for: Commensal gut bacteria employ de-chelatase HmuS to harvest iron from heme
Source: EMBO J. 2025 Sep 12;44(21):6226–52. doi: 10.1038/s44318-025-00563-5 (PMC12583661; doi:10.1038/s44318-025-00563-5)
Supplement: Supplementary file 9 — Source data Fig. 3 [file 44318_2025_563_MOESM9_ESM.zip › Fig. 3/Fig 3d/README_Fig3d.docx]

Figure 3d shows UV/visible absorbance spectra for the HmuS protein as it is titrated with increasing amounts of hemin solution. Each spectrum was measured relative to an identical cuvette containing buffer into which the equivalent amount of heme was added. This is a standard procedure for heme-protein titrations. Data columns are labeled according to the number of stoichiometric equivalents of heme that have been added to the protein (6 micromolar). Data were plotted using Kaleidagraph. These are the same data as in Figure 3c, but the plot is windowed onto the low energy bands.
