# Supplementary material for: Commensal gut bacteria employ de-chelatase HmuS to harvest iron from heme
Source: EMBO J. 2025 Sep 12;44(21):6226–52. doi: 10.1038/s44318-025-00563-5 (PMC12583661; doi:10.1038/s44318-025-00563-5)
Supplement: Supplementary file 9 — Source data Fig. 3 [file 44318_2025_563_MOESM9_ESM.zip › Fig. 3/Fig 3g/README_Fig3g.docx]

Figure 3g shows UV/visible absorbance spectra for the HmuS protein (6 micromolar), to which a stoichiometric excess ferric heme was subsequently added. This was followed by several rounds of concentration and resuspension in fresh buffer to remove loosely bound heme. Reductant (NADH) was added anaerobically, and spectra were measured at the given time points. Data were plotted using Kaleidagraph.
