# Supplementary material for: Commensal gut bacteria employ de-chelatase HmuS to harvest iron from heme
Source: EMBO J. 2025 Sep 12;44(21):6226–52. doi: 10.1038/s44318-025-00563-5 (PMC12583661; doi:10.1038/s44318-025-00563-5)
Supplement: Supplementary file 10 — Source data Fig. 4 [file 44318_2025_563_MOESM10_ESM.zip › Fig. 4/Fig_4.pptx]

## Slide 1
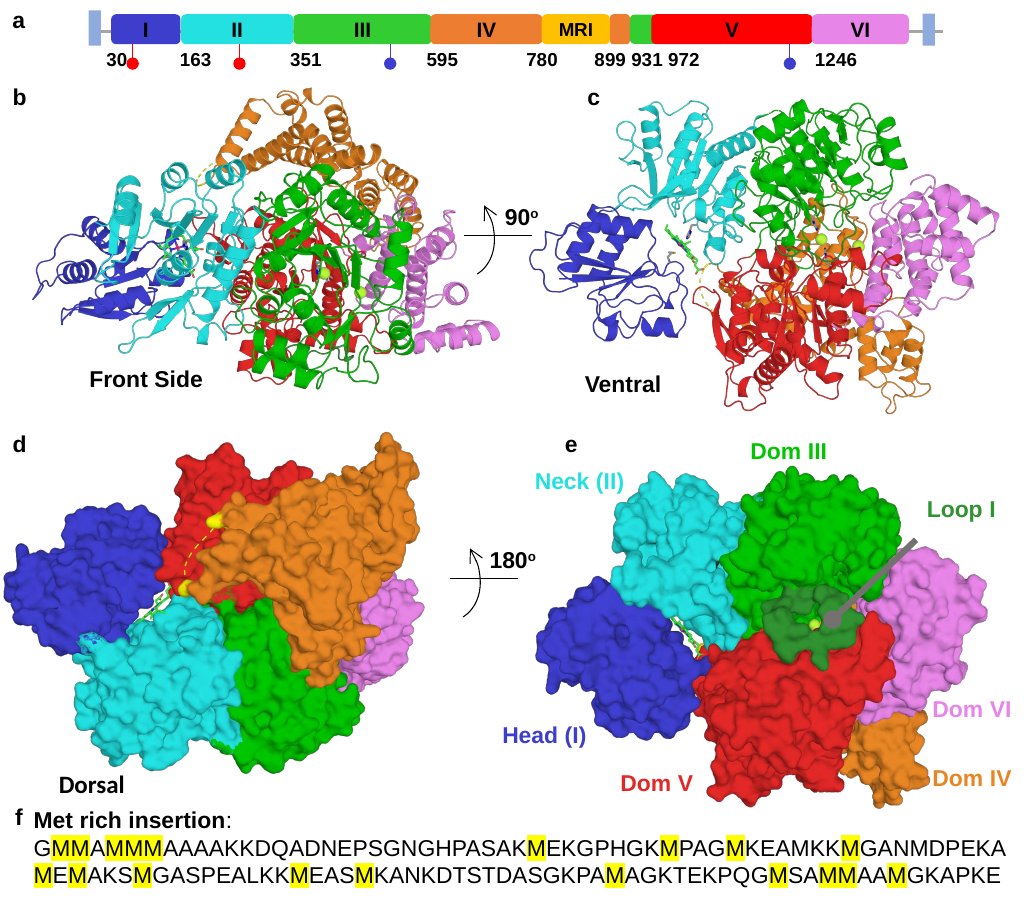

a
V
VI
I
II
III
IV
MRI
30 163 351 595 780 899 931 972 1246
b
c
90o
Front Side
Ventral
d
e
Dom III
Neck (II)
Loop I
Dom VI
Head (I)
Dom IV
Dom V
180o
Dorsal
f
Met rich insertion: GMMAMMMAAAAKKDQADNEPSGNGHPASAKMEKGPHGKMPAGMKEAMKKMGANMDPEKAMEMAKSMGASPEALKKMEASMKANKDTSTDASGKPAMAGKTEKPQGMSAMMAAMGKAPKE
