# Supplementary material for: Commensal gut bacteria employ de-chelatase HmuS to harvest iron from heme
Source: EMBO J. 2025 Sep 12;44(21):6226–52. doi: 10.1038/s44318-025-00563-5 (PMC12583661; doi:10.1038/s44318-025-00563-5)
Supplement: Supplementary file 11 — Source data Fig. 5 [file 44318_2025_563_MOESM11_ESM.zip › Fig. 5/Fig_5.pptx]

## Slide 1
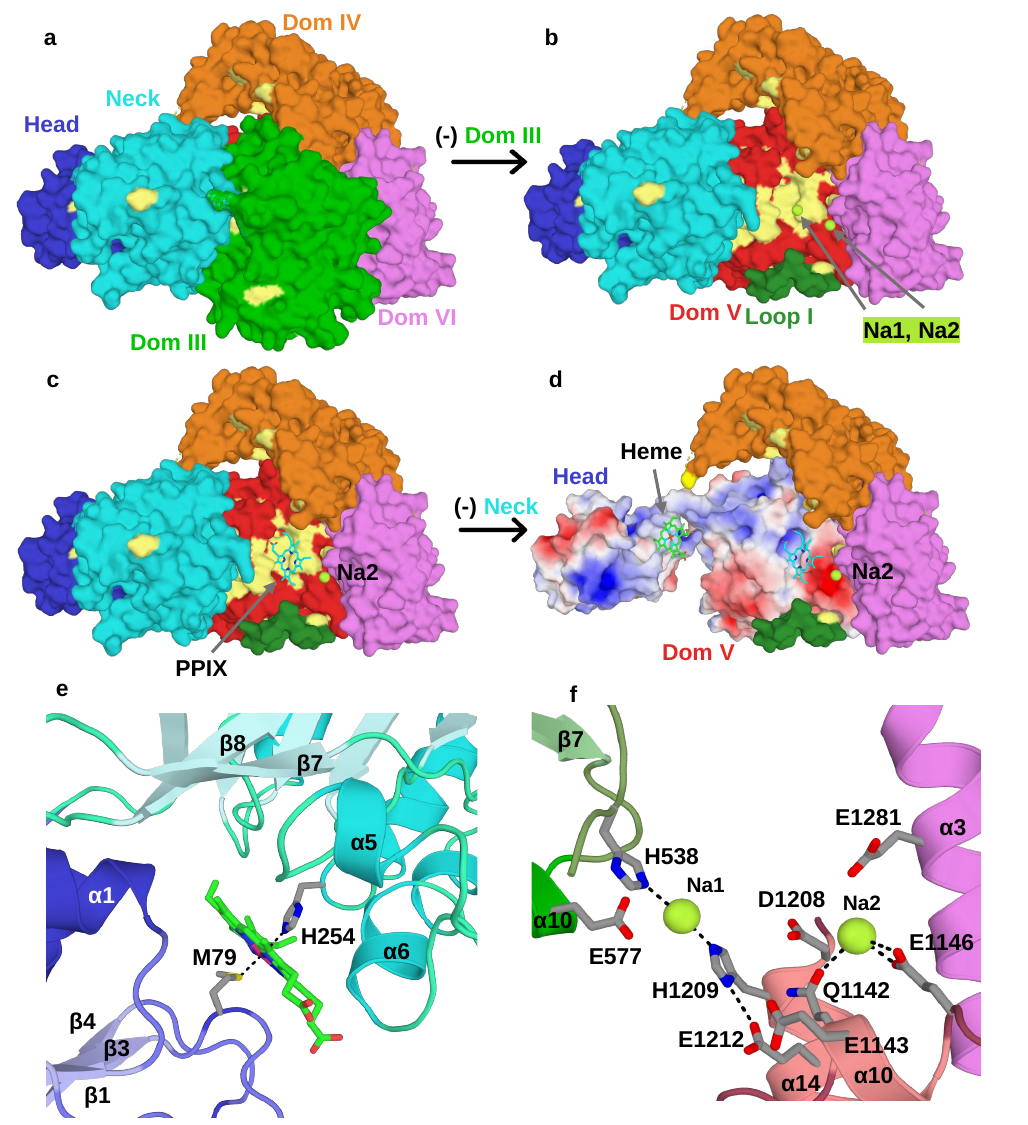

Dom IV
a
b
Neck
Head
(-) Dom III
Dom V
Loop I
Dom VI
Na1, Na2
Dom III
c
d
Heme
Head
(-) Neck
Na2
Na2
Dom V
PPIX
e
f
β7
E1281
α3
H538
D1208
α10
E1146
E577
H1209
Q1142
E1212
E1143
α10
α14
Na1
Na2
α1
H254
M79
β4
β3
β1
β8
β7
α5
α6
