# Supplementary material for: Commensal gut bacteria employ de-chelatase HmuS to harvest iron from heme
Source: EMBO J. 2025 Sep 12;44(21):6226–52. doi: 10.1038/s44318-025-00563-5 (PMC12583661; doi:10.1038/s44318-025-00563-5)
Supplement: Supplementary file 11 — Source data Fig. 5 [file 44318_2025_563_MOESM11_ESM.zip › Fig. 5/README_Fig5.docx]

The complete figure is provided in the top level of the directory as a single high-resolution image and as a modifiable PowerPoint montage.

Panels a-f in this figure were generated from a structural model fit to cryo electron microscopy structural data. The data and model have been deposited in the PDB as described in the text and provided in the folder for Figure 4. The individual panel images are provided in subfolders.

Text labels and arrows were added to individual panels using PowerPoint and imported into BioRender for further adjustment before rendering the entire figure montage as a single image.
