# Supplementary material for: Commensal gut bacteria employ de-chelatase HmuS to harvest iron from heme
Source: EMBO J. 2025 Sep 12;44(21):6226–52. doi: 10.1038/s44318-025-00563-5 (PMC12583661; doi:10.1038/s44318-025-00563-5)
Supplement: Supplementary file 12 — Source data Fig. 6 [file 44318_2025_563_MOESM12_ESM.zip › Fig. 6/Fig 6a/README_Fig6a.docx]

Figure 6a shows 2 UV/visible absorbance spectra numbered 1-2. Kaleidagraph was used to plot the data, which are representative of the spectra we routinely measure for these protein fractions.
