# Supplementary material for: Commensal gut bacteria employ de-chelatase HmuS to harvest iron from heme
Source: EMBO J. 2025 Sep 12;44(21):6226–52. doi: 10.1038/s44318-025-00563-5 (PMC12583661; doi:10.1038/s44318-025-00563-5)
Supplement: Supplementary file 12 — Source data Fig. 6 [file 44318_2025_563_MOESM12_ESM.zip › Fig. 6/Fig 6b-Gel/README_Fig6b.docx]

Figure 6b shows an SDS PAGE image from the two original images below that have been cropped to emphasize a molecular weight size marker and the same protein fractions (1-2) for which spectra are provided in Figure 6a.

Lanes 1 and 7 from the first source image are used as lanes “M” and “1” in the final image shown at the bottom. (This source image was also used in Figure 3B.) The second-to-last labeled lane from Source Image 2 is shown as “Lane 2” in the final image.

Source image 1:


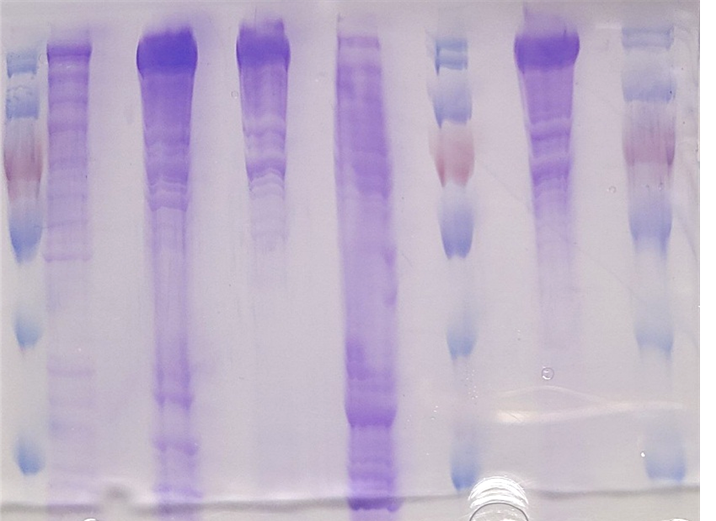


Source Image 2:


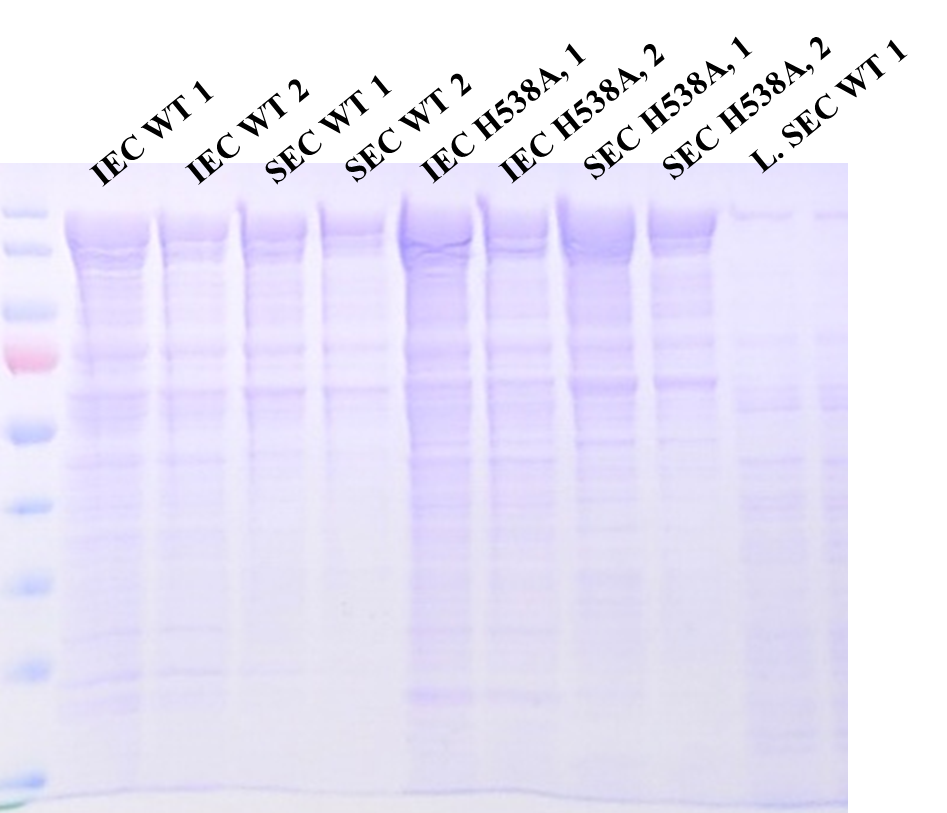


Final image:


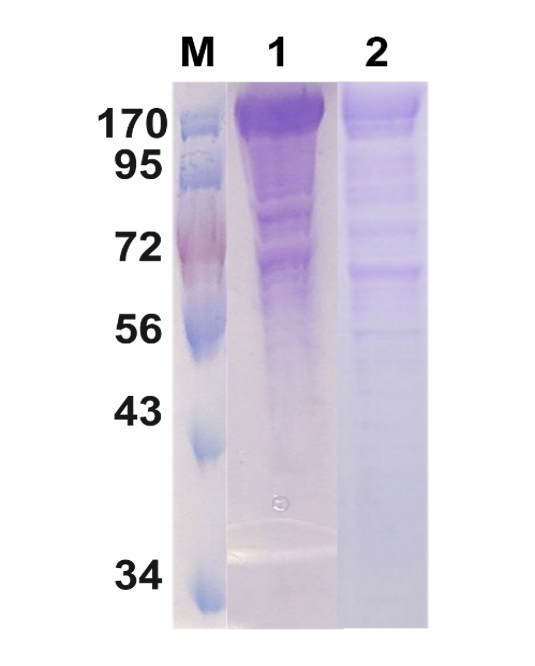


Aside from cropping, the images are unaltered. They were taken using a cell phone camera and two gels placed on a visible light table.

Black lines have been inserted to indicate the interfaces between the gel pieces.
