# Supplementary material for: Commensal gut bacteria employ de-chelatase HmuS to harvest iron from heme
Source: EMBO J. 2025 Sep 12;44(21):6226–52. doi: 10.1038/s44318-025-00563-5 (PMC12583661; doi:10.1038/s44318-025-00563-5)
Supplement: Supplementary file 12 — Source data Fig. 6 [file 44318_2025_563_MOESM12_ESM.zip › Fig. 6/Fig 6c/README_Fig6c.docx]

Bar graphs plotted using Kaleidagraph show averaged amounts of metabolites measured via HPLC relative to a standard curve, from a given reaction mixture. Error bars represent +/- standard deviation. Individual data points are given in the .csv file.
