# Supplementary material for: Commensal gut bacteria employ de-chelatase HmuS to harvest iron from heme
Source: EMBO J. 2025 Sep 12;44(21):6226–52. doi: 10.1038/s44318-025-00563-5 (PMC12583661; doi:10.1038/s44318-025-00563-5)
Supplement: Supplementary file 13 — Source data Fig. 7 [file 44318_2025_563_MOESM13_ESM.zip › Fig. 7/README_Fig7.docx]

This figure was drawn using ChemDraw 8.0. The original ChemDraw file and a copy saved as a .tif file are included here.
