# Supplementary material for: Commensal gut bacteria employ de-chelatase HmuS to harvest iron from heme
Source: EMBO J. 2025 Sep 12;44(21):6226–52. doi: 10.1038/s44318-025-00563-5 (PMC12583661; doi:10.1038/s44318-025-00563-5)
Supplement: Supplementary file 14 — Source data Fig. 8 [file 44318_2025_563_MOESM14_ESM.zip › Fig. 8/README_Fig8.docx]

Figure 8 depicts the sequence homologs of HmuS, including members of the HmuS, CobN, and ChlH families. The results are colored by taxonomic group. The plot was generated using t-SNE to create a 2D grouping of sequences, which cluster according to their degree of relatedness.

**Supplementary Document S5** is an HTML file that contains the data from Figure 8 but in an actively interrogatable form. Hovering a mouse pointer over each dot will show its BLAST-determined sequence identity to *B. theta* HmuS, followed by UniRef annotation of that sequence. This was included as a supplementary document with the submitted manuscript. A copy of Document S5 is also included in this subfolder.
