# Supplementary material for: Commensal gut bacteria employ de-chelatase HmuS to harvest iron from heme
Source: EMBO J. 2025 Sep 12;44(21):6226–52. doi: 10.1038/s44318-025-00563-5 (PMC12583661; doi:10.1038/s44318-025-00563-5)

Source image for Appendix Figure S8A:


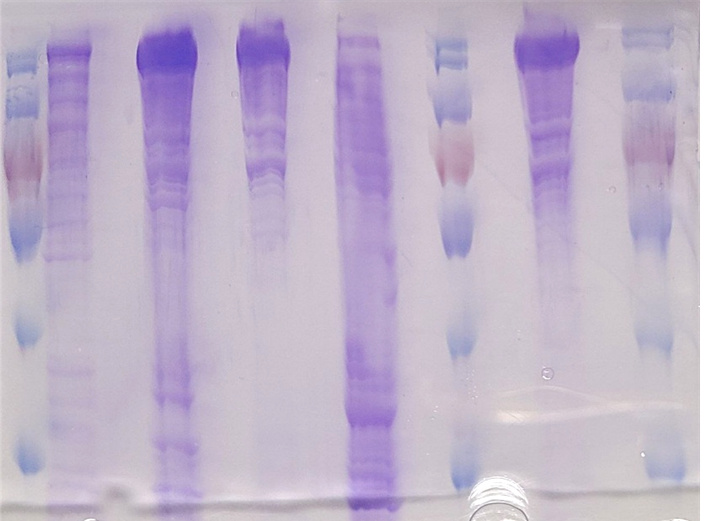


The same source image was used in Figures 3B and 6B to show the purity of the HmuS protein used in the study.

Lane 1 (marker), 2 (CFL), 3 (IEC), 5 (L. SEC) and 7 (SEC) were used in making Appendix Figure S8A. Black lines have been inserted to indicate the positions of the interfaces between gel pieces:


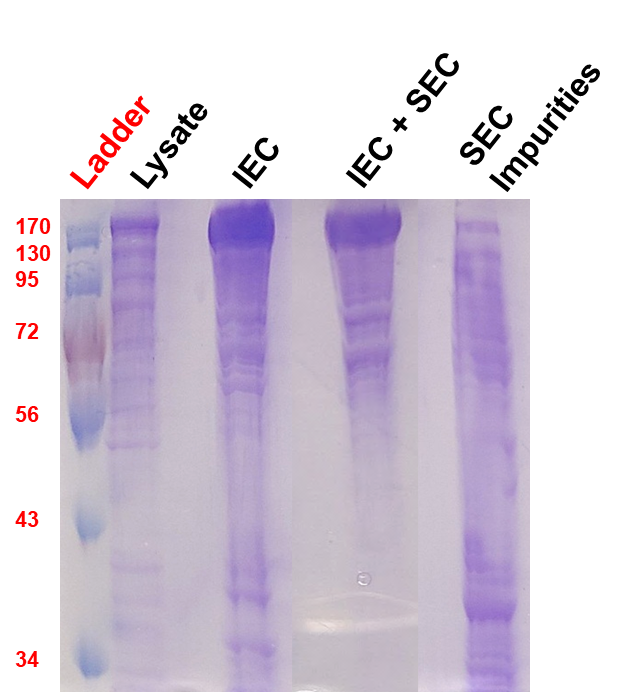


Source image for Appendix Figure S8D:


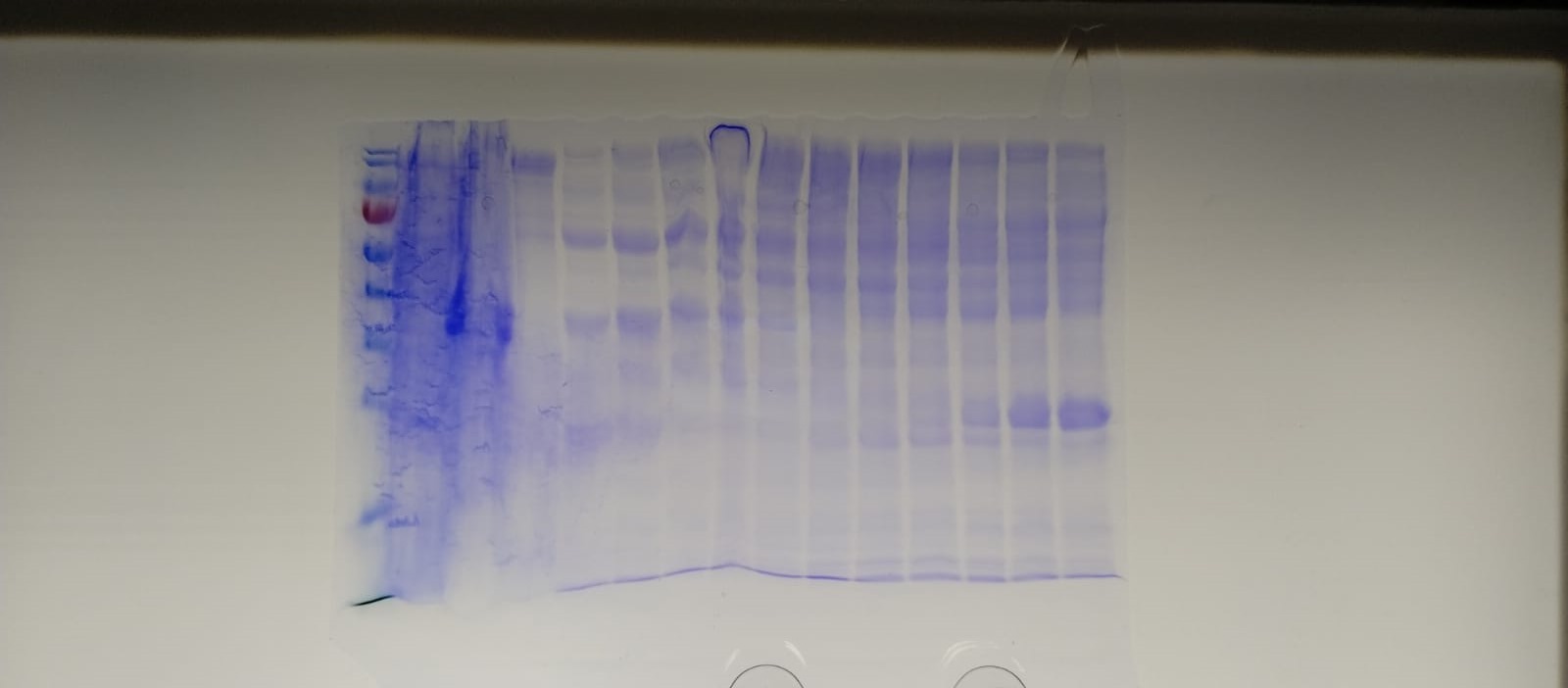


Lane 1 (marker) and the final 3 lanes were combined to give the final image. A black line has been inserted to indicate the position of the interface between the two gel pieces:


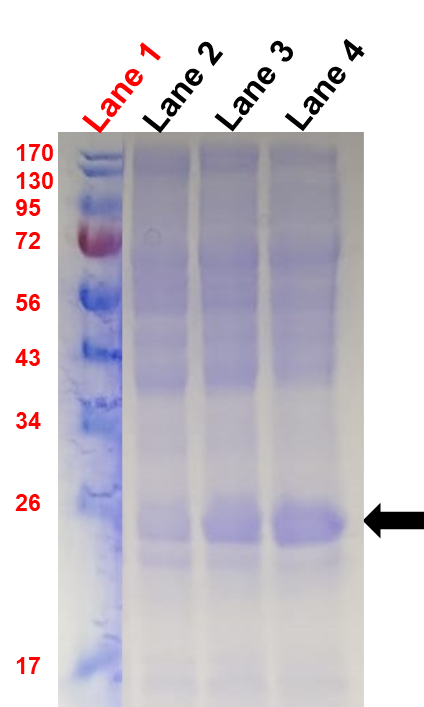

Supplement: Supplementary file 15 — Source data for Appendix Figure S8 [file 44318_2025_563_MOESM15_ESM.zip › Fig. S8/README FigS8.docx]
