# Supplementary material for: Commensal gut bacteria employ de-chelatase HmuS to harvest iron from heme
Source: EMBO J. 2025 Sep 12;44(21):6226–52. doi: 10.1038/s44318-025-00563-5 (PMC12583661; doi:10.1038/s44318-025-00563-5)
Supplement: Supplementary file 16 — Expanded View Figures [file 44318_2025_563_MOESM16_ESM.pdf]

Expanded View Figures

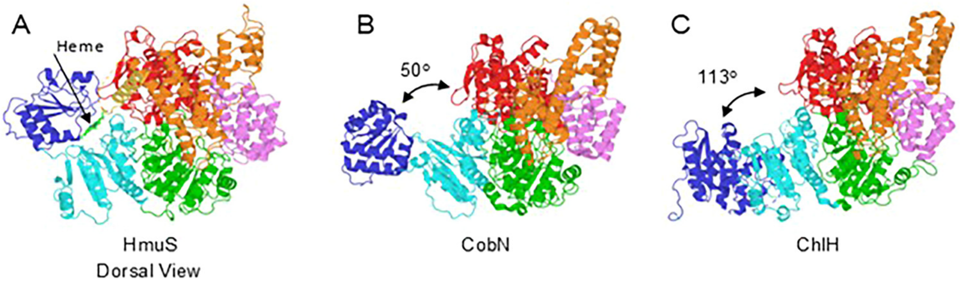

Figure EV1. Head domain conformations.

(**A**) HmuS is colored as in Fig. 4, with the head domain in blue, the neck in cyan, and domains III–VI in green, orange, red and purple. The orientation is a "dorsal" view, i.e., looking down on the domain IV "backbone", with the membrane roughly in the plane of the paper underneath HmuS, and heme bound at the head/neck interface. The first helix of the methionine-rich insertion (MRI) is shown in yellow, with the dashed line indicating a rough position for the 120 disordered residues of the MRI, potentially in position to interact with the heme-binding site at the neck/head interface. (**B**) CobN shown in an equivalent orientation. Domains II-VI superpose well on HmuS, but the head domain is rotated away from the neck domain by 50°. (**C**) ChlH in the same relative orientation, showing even greater movement of the head domain relative to the neck and body domains. Superposition of the HmuS head domain on the ChlH head domain requires a 113° rotation. The differing orientations of the head domain in CobN and ChlH suggest the HmuS head domain may also sample open conformations in the absence of heme.

© The Author(s)

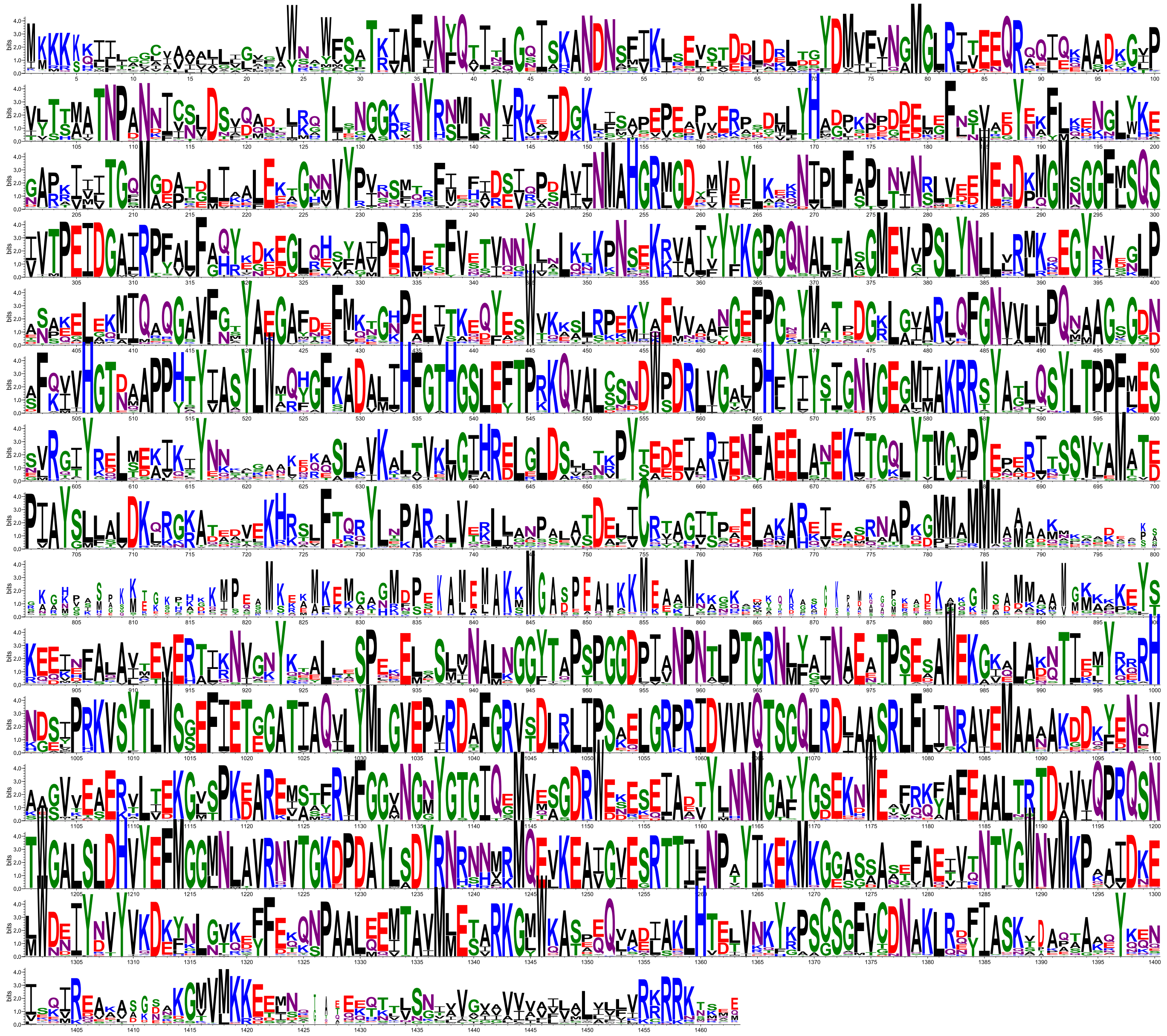

Figure EV2. Sequence logo representation illustrating conservation among operonic HmuS sequences.

As we included only operonic HmuS sequences with at least 50% identity and 95% coverage to *B theta* HmuS, this logo is most representative of organisms related to *B. theta*. See also Dataset EV4.

© The Author(s)

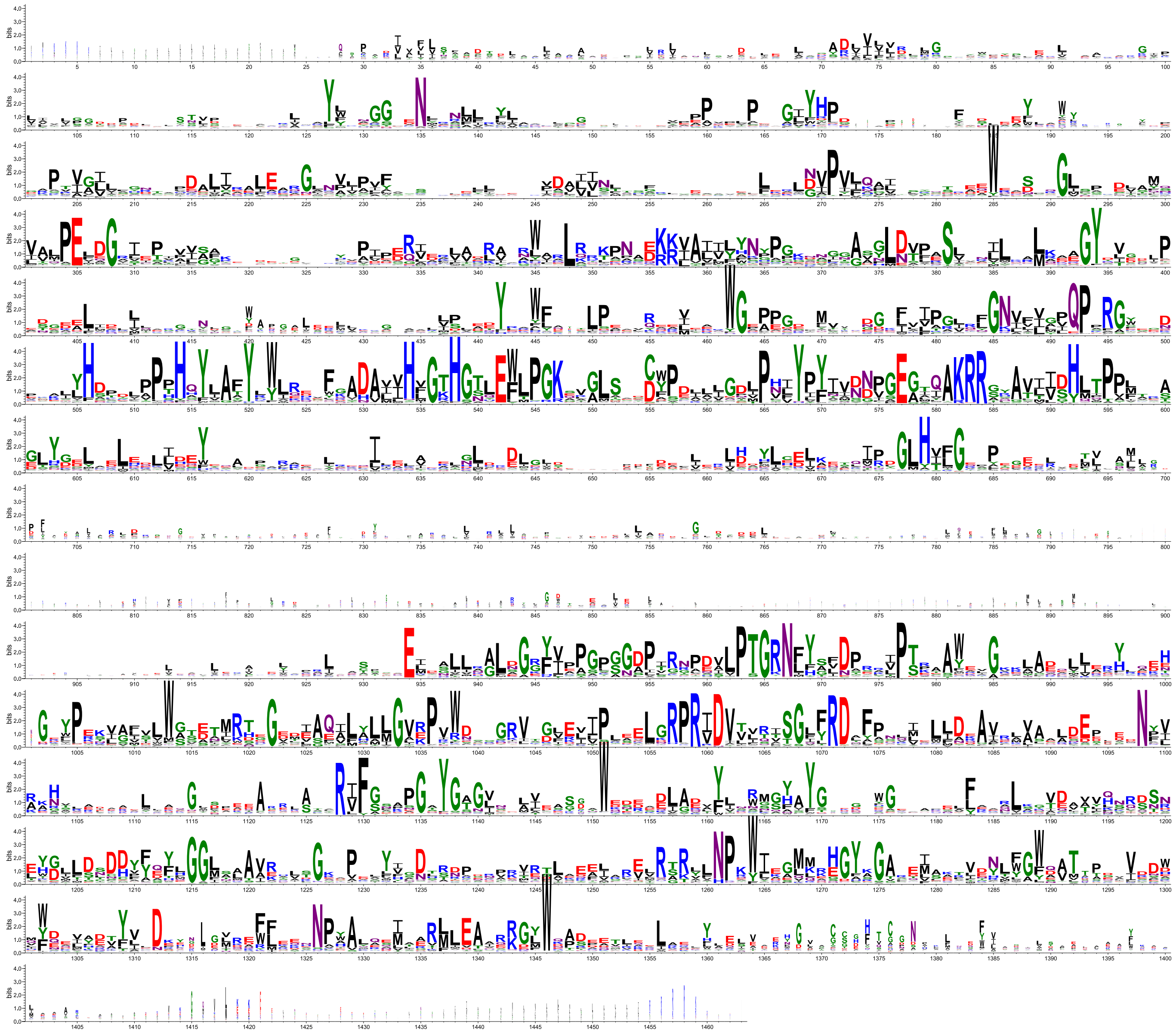

Figure EV3. Sequence logo representation illustrating conservation among type 1 chelatase sequences.

As the MRI is not found in chelatase sequences other than HmuS, the middle part of the sequence logo has almost no conservation.

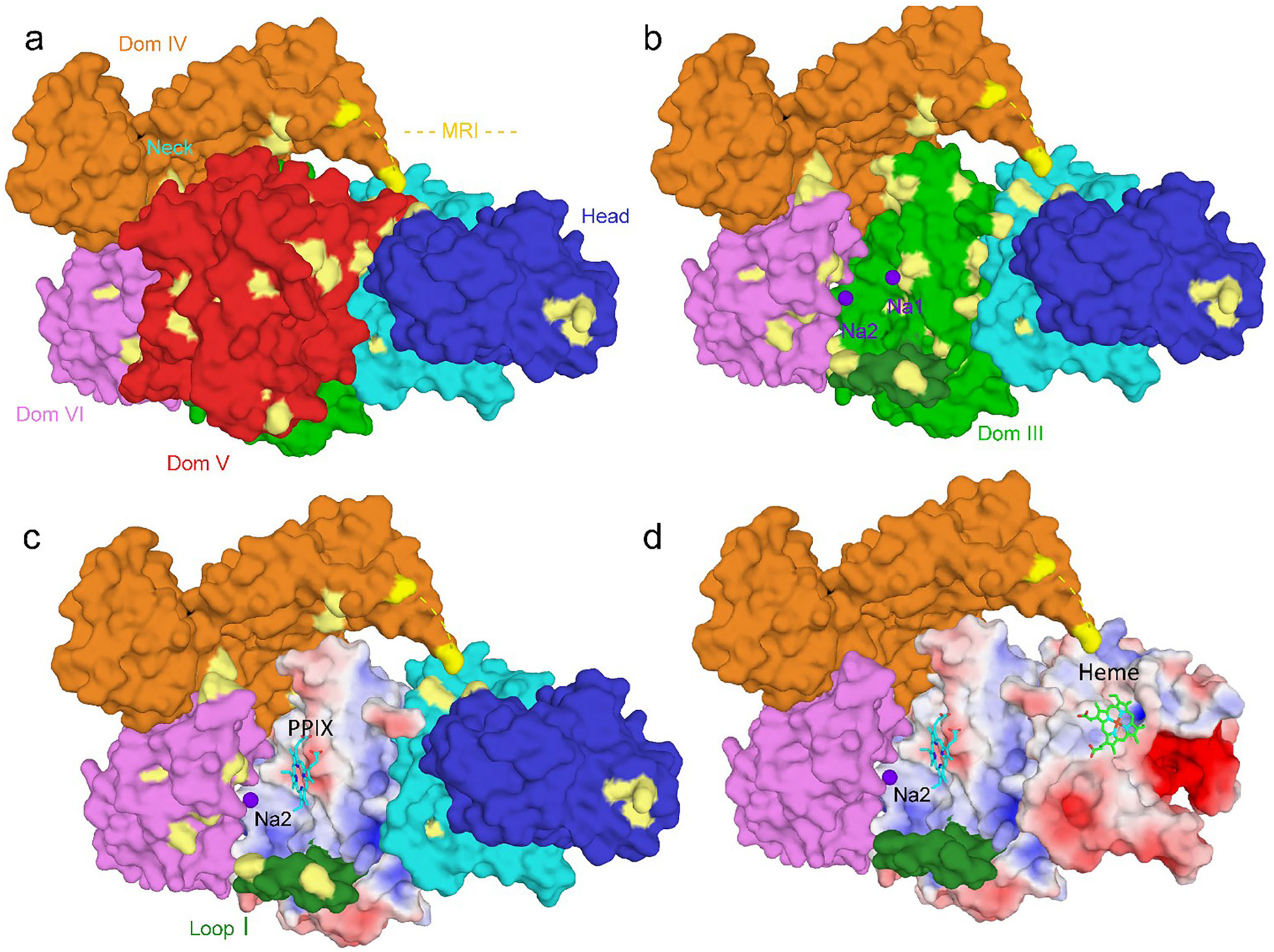

Figure EV4. Ligand binding sites, backside view.

(**A**) HmuS is shown from the “backside”, a 180° rotation about the vertical axis relative to Fig. 6. Domains are colored as in Figs. 5 and 6, with strictly conserved residues in pale yellow. The absence of the MRI is indicated by the yellow dashed line and yellow anchor points on domain IV (orange). (**B**) Domain V has been removed, revealing the central cleft with 2 bound Na ions (purple), Na1 and Na2, and Loop I at the bottom of the structure. Na1 is coordinated by strictly conserved His-1209, which lies in the center of the exposed domain III face. The domain III face of the central cleft is less conserved than the domain V face. (**C**) The docked protoporphyrin IX (PPIX) and Na2 are shown, along with the electrostatic surface domain III (+/− 5kT/e). (**D**) Domain I (blue) is also removed, revealing the heme-binding site at the Head/Neck interface. The heme and PPIX molecules are separated by nearly 30 Å. Electrostatic surfaces are also shown for domain II.

 © The Author(s)

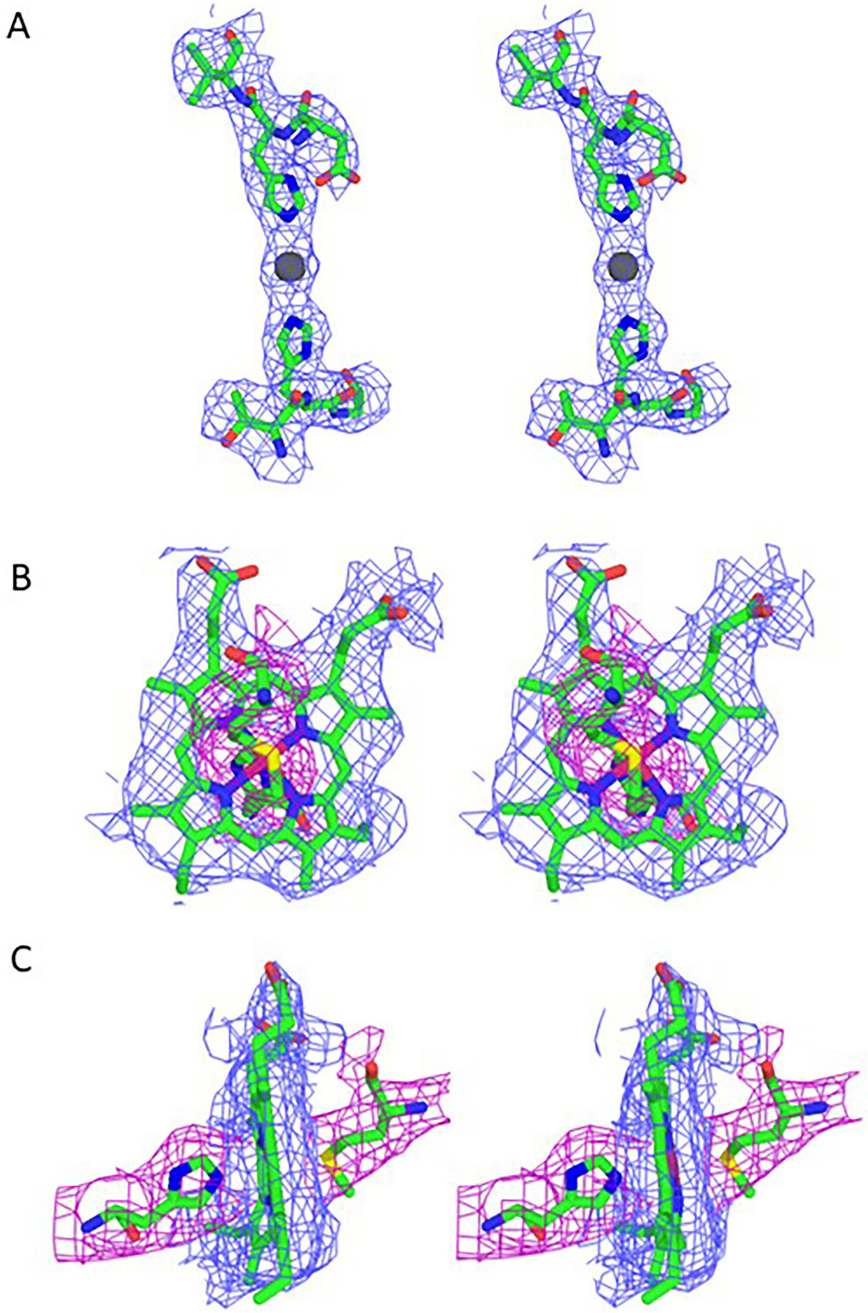

Figure EV5. Potential density maps.

(**A**) Stereo figure showing the potential density for Na1 and the coordinating histidine residues. Asp1208-His1209-Val1210 are positioned above Na1 while Gly537-His538-Thr539 are below. (**B**) Stereo figure showing the potential density for Met79, His254 and the heme group modeled at the interface of the head and neck domains. The isonet contoured around Met79 and His254 is in magenta, while that for the heme group is in blue. (**C**) As in (**B**), but rotated 90 degrees about the vertical axis to view the heme group edge on.
